# Mucoadhesive nanofibers for ocular drug delivery: mechanisms, design strategies, and applications

**DOI:** 10.1007/s13346-025-01894-w

**Published:** 2025-06-25

**Authors:** Nimeet Desai, Helen E. Colley, Yamini Krishna, Lucy A. Bosworth, Victoria R. Kearns

**Affiliations:** 1 Department of Eye and Vision Science, Institute of Life Course and Medical Sciences, University of Liverpool, Liverpool, L7 8TX UK; 2 School of Clinical Dentistry, University of Sheffield, Sheffield, S10 2TA UK; 3 Liverpool Clinical Laboratories, National Specialist Ophthalmic Pathology Service, University Hospitals of Liverpool Group, Liverpool, L7 8YE UK

**Keywords:** Nanofibers, Ocular drug delivery, Mucoadhesion, Electrospinning, Mucosa, Ocular surface

## Abstract

Delivering drugs effectively to the ocular surface is challenging due to rapid clearance mechanisms, including blinking, tear turnover, and protective barriers of the conjunctival and corneal epithelium. As a result, conventional options such as eye drops often fail to provide sustained therapeutic effects and require frequent dosing, leading to reduced patient compliance. Mucoadhesive nanofiber systems offer a promising solution by enhancing drug retention and enabling controlled release at the ocular surface. These nanofibers, produced primarily through electrospinning, provide a high surface area, tunable mechanical properties, and compatibility with mucoadhesive polymers, collectively improving drug bioavailability, extending residence times, and minimizing systemic side effects. This review comprehensively explores the fundamentals of mucoadhesion, including the structural and compositional characteristics of ocular mucosal surfaces and the molecular interactions essential for optimized drug delivery. It examines advanced strategies for incorporating mucoadhesive features into nanofibers, such as polymer blending, surface modification, and molecular imprinting, and assesses their influence on therapeutic outcomes. Finally, recent advancements and their potential for clinical translation are discussed. By presenting a thorough analysis of current techniques and emerging innovations, this review aims to guide researchers in developing next-generation mucoadhesive nanofiber platforms that improve therapeutic efficacy and patient compliance in ocular drug delivery.

**Graphical Abstract:**

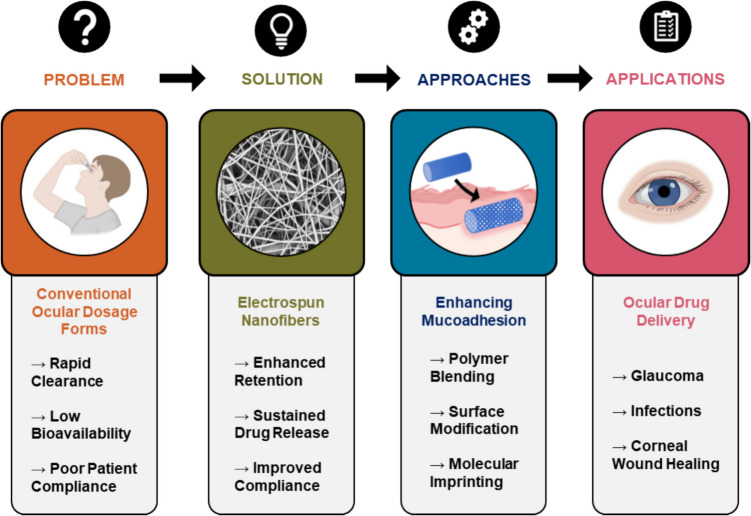

## Introduction

In recent years, pharmaceutical research has undergone a significant shift. Traditionally focused on discovering molecules with improved pharmacokinetics and pharmacodynamics, the field now increasingly emphasizes advanced drug delivery systems enabled by multifunctional biomaterials [1]. These systems address biological and physicochemical barriers to enhance bioavailability, sustain therapeutic levels, and improve patient compliance [2]. The emphasis has moved toward controlled and sustained release to reduce side effects and achieve targeted, patient-centered therapy [3]. Ocular drug delivery remains particularly challenging due to the eye’s unique anatomy and physiology. The conjunctiva, cornea, and tear film protect ocular function but also limit drug absorption [4]. While the epithelium restricts hydrophilic drug uptake, the stroma hinders lipophilic molecules. Additionally, tear turnover, nasolacrimal drainage, and blinking rapidly clear drugs, lowering bioavailability [5]. Conventional approaches like eye drops and ointments offer only short-term exposure and require frequent dosing [6, 7]. Many therapeutics are further limited by poor compatibility with ocular surface delivery and enzymatic degradation in the tear film [8]. These limitations highlight the need for delivery systems that improve ocular retention and therapeutic efficiency.

Recent advances in nanotechnology and biomaterials have introduced nanofiber-based platforms as promising solutions for ocular drug delivery. Nanofibers, typically fabricated via electrospinning, offer high versatility and robustness. This technique uses an electric field to draw ultrafine fibers from a polymer solution or melt, yielding structures with high surface area-to-volume ratios that support enhanced drug loading [9]. Their porous architecture accommodates both hydrophilic and lipophilic drugs [10]. Nanofibers are also mechanically stable and suitable for ocular conditions [11]. Through optimized polymer blends and electrospinning parameters, nanofibers can be tuned for controlled drug release, from immediate to sustained delivery for acute or chronic conditions [12]. Their large surface area ensures close contact with the ocular mucosa, increasing absorption and local drug concentration [13]. A key advancement is the integration of mucoadhesive properties into nanofiber systems. Mucoadhesion allows polymers to bind to the mucin-rich tear film, prolonging residence time on the ocular surface via hydrogen bonding, van der Waals forces, and electrostatic interactions [14]. These interactions, influenced by material composition and fabrication conditions, enhance ocular retention and drug delivery efficiency. Strengthening mucoadhesive properties can reduce dosing frequency and improve outcomes, overcoming limitations of conventional methods [15].

This review examines the integration of mucoadhesive properties into nanofiber-based drug delivery systems. It first discusses the ocular mucosa and its barriers to drug absorption, followed by an exploration of mucoadhesion mechanisms and the advantages of nanofibers over traditional drug delivery methods (like eye drops, ointments, and intravitreal injections). The latter sections focus on the design, fabrication, and application of mucoadhesive nanofibers in managing various ocular diseases, highlighting their transformative potential in achieving targeted, sustained, and effective therapy.

## Understanding the ocular surface and mucoadhesion

### Structure and role of the ocular surface

The conjunctiva is a specialized ocular mucosa or mucous membrane that protects and lubricates the anterior surface of the globe (bulbar conjunctiva) and the posterior surface of the eyelids (palpebral/tarsal conjunctiva), thus maintaining ocular surface homeostasis and offering protection from infections [16]. This tissue consists of a modified non-keratinizing stratified squamous epithelium that transitions to stratified columnar epithelium containing goblet cells, overlying a loose connective tissue layer known as the lamina propria or stroma. The goblet cells, along with other glandular structures, secrete mucins, which are high-molecular-weight glycoproteins critical for tear film stability and ocular surface protection [17] (Table 1). For instance, gel-forming mucins such as MUC2, MUC5AC, and MUC19 are primarily secreted by goblet cells [18], with MUC5AC also present at the apical surface of goblet cells and in the lid wiper, contributing to the tear film’s viscosity and aiding in debris clearance [19]. Transmembrane mucins like MUC1, MUC4, and MUC16, produced by the lacrimal glands and apical corneal and conjunctival epithelial cells, are found in the extracellular domain of tears, where they help anchor the tear film to the ocular surface and provide a protective barrier [20, 21]. In contrast, soluble MUC7 from the lacrimal glands and stratified epithelium [22, 23], as well as transmembrane mucins such as MUC13, MUC15, MUC17, and MUC20 from basal and intermediate epithelial layers, are not typically detected in tears, suggesting a more localized role in cellular protection rather than tear film composition [24]. In-depth details about ocular mucins are covered in paper [25].

**Table 1:** Ocular mucins type and location. Reproduced with permission from [17], Copyright Elsevier 2019

Type of mucins	Ocular location	Identified in tears
MUC1	Transmembrane, Lacrimal glands, Apical corneal and conjunctival epithelial cells	Yes (Extracellular domain)
MUC2	Gel-forming, Goblet cells	Yes
MUC4	Transmembrane, Cornea, conjunctiva, lacrimal glands, Apical conjunctival epithelial cells	Yes (Extracellular domain)
MUC5AC	Gel-forming, Apical surface of GCs, Lid wiper	Yes
MUC7	Soluble, Lacrimal glands, Stratified epithelium	No
MUC13	Transmembrane	No
MUC15	Transmembrane	No
MUC16	Transmembrane, Apical corneal epithelial surface, Lacrimal gland ductal epithelial cells	Yes (Extracellular domain)
MUC17	Transmembrane	No
MUC19	Gel-forming, Goblet cells	No
MUC20	Transmembrane, Basal and intermediate epithelial cell layer	No

**Fig. 1:**
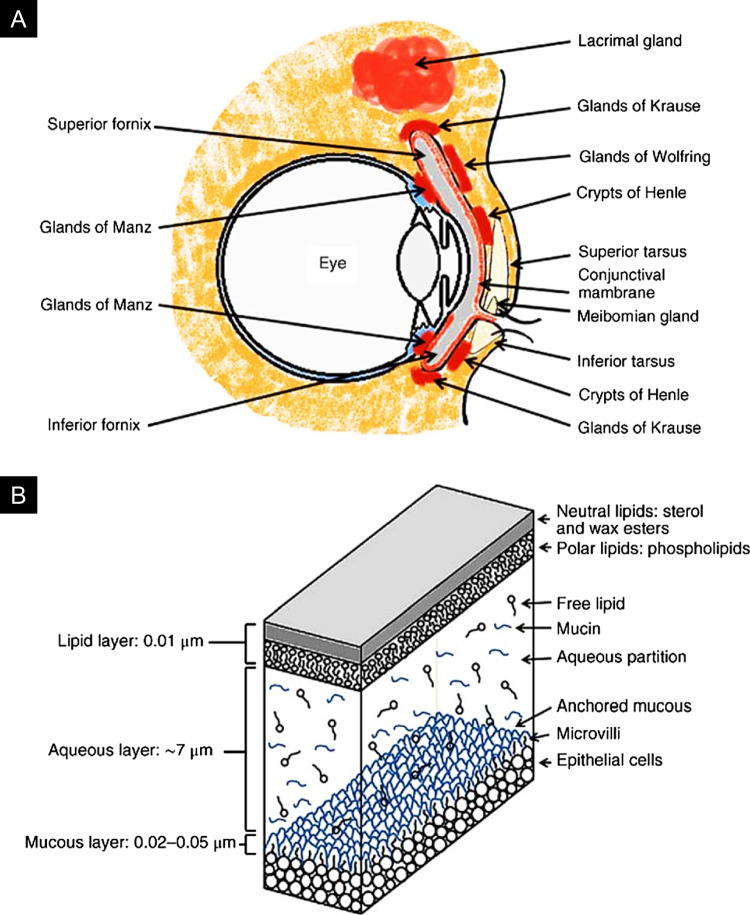
**A** Anatomical depiction of the ocular mucosa, showcasing the locations of glands responsible for the secretion of mucin, aqueous, and lipid components. **B** Schematic representation of the three-layer tear film model, highlighting the mucin, aqueous, and lipid layers. Reproduced with permission from [35], Copyright Wiley, 2014

The conjunctiva also houses accessory lacrimal glands, such as the glands of Krause located in the fornices and glands of Wolfring along the upper border of the tarsus, which secrete aqueous tear constituents essential for hydration and lubrication [26]. Additional mucus is produced by the glands of Henle, and lipid production originates from the Meibomian glands within the tarsal plates and the glands of Zeis at the eyelid lash follicles (Fig. 1A) [27, 28]. The primary source of aqueous tears is the lacrimal gland, situated in the superolateral orbital quadrant just behind the orbital margin. The tear film, measuring approximately 7 to 9 µm thick, comprises three distinct layers (Fig. 1B). The superficial lipid layer, secreted by the tarsal Meibomian and Zeis glands, minimizes evaporation, preserves the aqueous content beneath, and provides tear film stability. The aqueous layer, primarily produced by the lacrimal gland with dispersed mucin from conjunctival sources, facilitates hydration, nutrient transport, debris clearance, and antimicrobial protection through lysozyme activity [29]. The innermost mucus layer, secreted by conjunctival goblet cells and epithelial cells and anchored to the conjunctival and corneal epithelial cell microvilli, ensures the adhesion of the aqueous layer to the conjunctival surface, promoting tear film stability and smooth ocular functionality [30].

Tears serve critical functions, including moistening the ocular surface to prevent damage to delicate tissues, smoothing corneal surface irregularities, delivering oxygen and nutrients, and acting as a pH buffer to ensure ocular surface stability. In the human eye, tear volume is typically around 7 to 9 µL, with a maximum capacity of 30 µL before blinking induces drainage. The tear fluid contains electrolytes, glucose, and numerous proteins including enzymes, maintaining a pH range of 7.0 to 7.5, regulated by a bicarbonate-carbon dioxide buffer system that adjusts dynamically based on whether the eye is open or closed, influencing carbon dioxide levels and acid–base balance [31, 32]. Lacrimation, or tear production, operates autonomously under parasympathetic control to maintain continuous ocular lubrication and also functions as a reflexive response to stimuli such as irritants or foreign bodies, triggering excess tear production to flush away irritants, with hormonal influences present though not clearly defined [33]. Additional mucin secretion is contributed by Manz glands and Henle crypts, complementing the goblet cells. Blinking ensures the even distribution of tears and mucus across the ocular surface, promoting hydration and a smooth, while reflex tearing in response to severe irritation generates an overflow that clears foreign material from the ocular surface [25]. The lacrimal apparatus, comprising the lacrimal gland, puncta, canaliculi, nasolacrimal sac, and nasolacrimal duct, facilitates tear elimination. Eyelid movements create pressure changes that force tears through this collecting system into the nose [34].

The ocular mucosa, despite its protective roles, is vulnerable to pathologies and presents significant challenges for drug delivery. First, this delicate tissue is susceptible to various conditions. Dry eye syndrome, resulting from diminished tear production or excessive evaporation, compromises tear film integrity, causing irritation, corneal damage, and visual impairment [36]. Conjunctivitis, an inflammation of the conjunctiva triggered by infections or allergies, disrupts the mucosal barrier, exacerbating discomfort and redness [37, 38]. Ocular surface cancers, such as conjunctival melanoma, squamous cell carcinoma, or sebaceous carcinoma, further highlight the fragility of these tissues, as tumor growth undermines structural integrity and complicates localized therapeutic approaches [39, 40]. Second, the physiological and histological architecture of the ocular mucosa and cornea creates substantial barriers to drug penetration. The corneal epithelium, a lipophilic layer with tight junctions, restricts the penetration of hydrophilic drugs, allowing efficient permeation only for small, lipophilic molecules with an optimal log P of 2 to 3 [41]. Beneath this, the hydrophilic stroma poses an additional barrier to lipophilic drugs, necessitating amphiphilic properties for effective corneal penetration [42]. Third, pre-corneal drug clearance mechanisms significantly reduce the residence time of topically applied drugs, with less than 5% typically reaching therapeutic targets. Reflex tearing, blinking, and nasolacrimal drainage, combined with enzymatic degradation within the tear film, further limit drug stability, complicating efforts to maintain therapeutic concentrations [43, 44]. The emergence of mucoadhesive drug delivery systems offers a promising strategy to overcome these interconnected challenges by enhancing drug retention and aligning structural innovation with therapeutic efficacy, thereby improving the potential for effective ocular drug delivery.

### Theories/mechanism of mucoadhesion

**Fig. 2:**
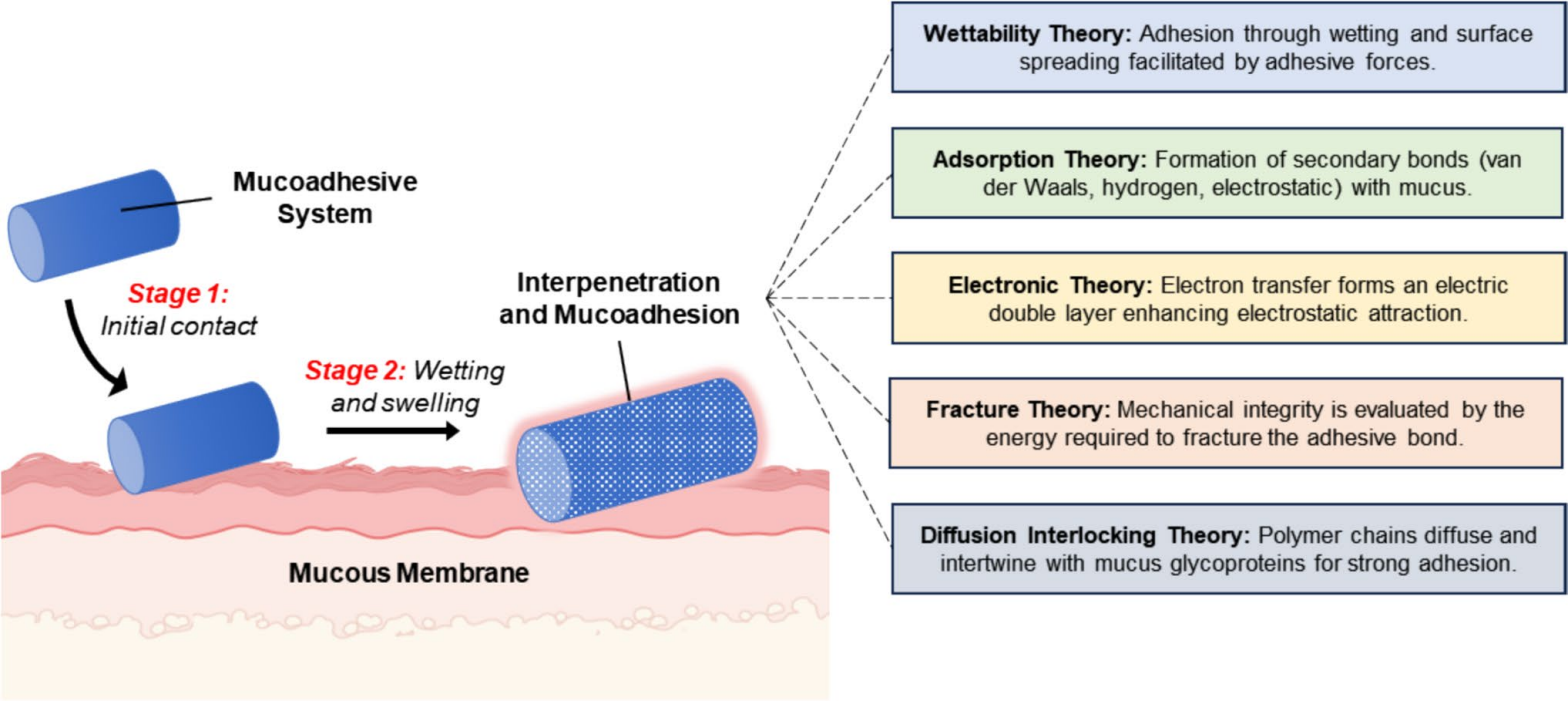
The process, stages, and proposed theories of mucoadhesion. The first stage (contact stage) involves the mucoadhesive drug delivery system making close contact with the mucous membrane, primarily through a wetting process. The second stage (consolidation) strengthens this contact through various physicochemical interactions, ensuring prolonged adhesion and effective drug delivery. Several theories that explain how drug delivery systems interact and adhere to mucosal membranes have been proposed (summarized in the right panel). *Created with BioRender.com*

Mucoadhesion has been a key area of research since the 1980 s, offering deep insights into how formulations interact with mucus and the factors that influence these interactions. Over the years, numerous theories have emerged to explain the mechanisms behind mucoadhesion. Generally, these mechanisms can be categorized into two primary stages. The initial contact stage involves the bioadhesive forming a close connection with the mucosal membrane, aided by processes like wetting or swelling [45]. In the subsequent consolidation stage, the bioadhesive becomes hydrated and swells, either by penetrating the mucosal tissue or adhering to the mucous membrane's surface through a process called interpenetration [46, 47]. These stages are fundamental to mucoadhesion and are described by several established theoretical frameworks (Fig. 2).

- **Wettability theory:** This theory is primarily applied to mucoadhesive systems that are liquid or have low viscosity. Wettability refers to a material's ability to adhere to mucosal surfaces via intermolecular interactions [48]. It is influenced by the balance between adhesive forces (liquid–solid interactions) and cohesive forces within the liquid. Upon contact, the mucoadhesive penetrates surface irregularities and adheres due to changes in surface and interfacial energies [49]. Contact angle goniometry evaluates wettability by measuring the contact angle; smaller angles indicate stronger adhesion [50]. This mechanism reflects the energy needed to overcome the surface tension at the interface between the mucoadhesive and the mucosa, promoting effective biological substrate spreading and exposure [51]. Experimentally, the contact angle (θ) is linked to the interfacial tension (γ) between the mucosal surface and the mucoadhesive system according to the equation:

$$\gamma SV-\gamma SL=\gamma LVcos\theta$$

Here, γSV is the tension between the solid and vapor, γSL is the tension between the solid and liquid, γLV is the liquid's surface tension, and θ represents the contact angle. According to this concept, bioadhesive systems designed with structures and functional groups that align with and conform to the mucosal surfaces exhibit improved physicochemical compatibility, ensuring optimal wettability, adhesion, and sustained drug retention across the ocular surface. The spreading efficiency of a bioadhesive polymer increases as its contact angle nears zero, facilitating mucin interaction with the polymer, which enhances its ability to spread [52].

- **Adsorption theory:** This theory proposes that mucoadhesion primarily results from secondary interactions between the mucoadhesive material and mucosal tissues. These include van der Waals forces, hydrogen bonding, and electrostatic interactions, which together form a stable adhesive interface [53].

Van der Waals forces, though weak, arise from transient electrical interactions and become relevant when numerous contact points are involved between the adhesive and mucosal surface [54, 55]. Hydrogen bonds, which are stronger, typically form between electronegative atoms (e.g., N, O, or F) and hydrogen atoms on polymer functional groups such as hydroxyl, carboxyl, or amino, and complementary groups in mucins [56, 57]. Additionally, electrostatic attractions often develop between oppositely charged regions, especially when mucoadhesive materials are designed to align with the ionic nature of mucosal surfaces [58]. The overall bond strength and duration depend on the polymer's chemical structure, physical state, and the mucosal surface’s properties [59].

- **Electronic theory:** This theory explains mucoadhesion based on electrostatic properties and electron transfer between materials with differing electronic characteristics [60]. When a polymer with higher electron density (or lower work function) contacts one with lower density (or higher work function), electrons transfer and form an electric double layer at the interface, consisting of oppositely charged regions on each surface [61, 62].

This double layer generates electrostatic attraction, significantly reinforcing the adhesive bond. Charge redistribution at the interface can induce dipole moments even in initially non-polar materials, expanding the zone of electrostatic interaction [63]. The work function of a material, defined as the energy required to remove an electron, plays a central role in this process. Polymers with low work functions, such as chitosan or polyaniline, donate electrons more readily, promoting adhesion with negatively charged mucins. In contrast, polymers like polyacrylic acid and alginate have higher work functions and tend to accept electrons, supporting electrostatic and hydrogen bonding interactions [64]. Understanding how work function differences between polymers and mucin influence electron transfer helps predict the strength and direction of adhesion [65].

- **Fracture theory:** This theory evaluates the mechanical integrity of mucoadhesive bonds by treating the interface as a separate material layer and analyzing the energy required to break it. The central idea is that the bond behaves as a distinct mechanical interface subjected to external force [66, 67]. The fracture strength (σ) can be calculated using the relationship between Young's modulus of elasticity (E), the fracture energy (ɛ), and the critical crack length (L) with the equation:

$$\upsigma =\sqrt{\left(\mathrm{E}\times\upvarepsilon \right)/L}$$

Fracture mechanics are classified into cohesive fractures, which occur within the adhesive, and adhesive fractures, which occur at the polymer–mucosa interface [68, 69]. The bond strength depends on the fracture energy, defined as the work done per unit area to create a new surface. Fracture can occur in different modes: Mode I (tensile separation), Mode II (shear displacement), and Mode III (torsional shear) [70]. These dynamics are influenced by properties like polymer elasticity and viscosity, mucosal surface roughness and hydration, as well as environmental conditions such as temperature and humidity.

- **Diffusion interlocking theory:** This theory suggests that mucoadhesion results from the diffusion of polymer chains into the mucus gel layer on mucosal surfaces. The degree of interpenetration and entanglement between polymer and mucus chains determines the strength and duration of adhesion [71]. The process begins when a mucoadhesive polymer contacts the mucus layer and swells, increasing the contact area and promoting chain interaction. Slight solubility in mucus is essential for effective swelling and diffusion [72]. The extent of diffusion depends on factors such as polymer molecular weight, flexibility, and chemical compatibility with mucus. Entanglement occurs when the polymer chains interlock with mucin glycoproteins, and this is further stabilized by entropic mixing forces [73]. Higher molecular weight polymers diffuse more slowly but form stronger bonds. Increased polymer concentration can raise viscosity, slowing diffusion yet enhancing chain density and interlocking potential, especially when the number average molecular weight is high [74]. Recent research using Fourier Transform Infrared (FTIR) spectroscopy and rheology has validated the estimated time (t) needed to achieve optimal adhesion through interpenetration [75, 76]. This duration can be mathematically represented by the equation:

$$T={L}^{2}/Db$$

Here, L denotes the penetration depth, and Db represents the diffusion coefficient. This formula helps predict the optimal interaction time for achieving the strongest mucoadhesive bond. The penetration depth of polymer chains into the mucus layer is crucial. The bonding process, encompassing diffusion and entanglement, is time-dependent; initial contact may lead to weak adhesion, strengthening gradually as more polymer chains diffuse and intertwine with the mucus network. Deeper penetration can result in a more substantial entanglement and, thus, a stronger mucoadhesive bond [77, 78]. However, too much diffusion can complicate the removal of the delivery system, which is an important consideration for products that require quick turnover, such as those used in gastrointestinal drug delivery [79].

While each mucoadhesion theory offers valuable insights, real-world applications often require a combination of these principles rather than reliance on a single model. Electrostatic interactions (electronic theory) and hydrogen bonding (adsorption theory) enhance mucin-polymer interactions, but prolonged adhesion also depends on polymer interpenetration (diffusion interlocking theory). Wettability theory applies mainly to liquid formulations, whereas fracture theory is crucial for solid dosage forms like nanofibers. It must be mentioned that not all theories apply equally to every mucoadhesive system. For ocular drug delivery, strong interfacial interactions (adsorption and diffusion interlocking theories) are more relevant than mechanical interlocking (fracture theory), which suits tissue adhesives. Optimizing mucoadhesive nanofibers requires selecting polymers and fabrication techniques that align with the dominant adhesion mechanisms. Achieving strong yet reversible adhesion while maintaining biocompatibility and sustained drug release depends on balancing polymer flexibility, charge distribution, and hydration properties. In practice, this often requires integrating materials, such as hydrophilic polymers with cationic copolymers, to achieve synergistic improvements in adhesion and efficacy across different ocular disease environments.

The physiology of ocular mucosa, along with the extensive theories on mucoadhesion discussed previously, highlights several key factors that are crucial for developing an effective mucoadhesive drug delivery system for the eye. These factors include various chemical, physical, and biological aspects affecting mucoadhesive polymers'interaction with mucosal tissues [80]. Each of these factors has a specific role in enhancing or restricting drug delivery efficiency through mucosal barriers. Table 2 details a comprehensive description and discussion of these factors and their impact on mucoadhesion.

**Table 2:** Insights into the factors influencing mucoadhesion

Factor	Description	Impact on Mucoadhesion	Ref
Molecular weight of polymer	Refers to the polymer size, typically measured by the mass of one mole of chains; higher molecular weights indicate longer chains	**•** Higher molecular weights promote stronger mucoadhesion by enabling extensive chain entanglement and multiple interaction sites with mucosal surfaces, resulting in more durable adhesive bonds **•** Excessively high molecular weights may impair polymer flexibility and processability, potentially limiting formulation efficiency and mucoadhesive performance	[81, 82]
Concentration of polymer	Refers to the polymer’s ratio within a formulation; higher concentrations create a denser polymer chain network	**•** Higher polymer concentrations enhance mucoadhesion by increasing the density of adhesive sites, which is particularly beneficial in environments with high mucosal turnover **•** Excessive polymer concentrations can lead to high viscosity, making formulations difficult to apply and potentially hindering drug release	[83, 84]
Swelling factor	Refers to the ability of a polymer to absorb water and swell on mucosal contact, essential for hydrophilic polymers to achieve adhesion	**•** Polymer swelling increases contact area and promotes deeper mucosal penetration, enhancing mechanical interlocking and overall adhesive strength **•** Controlled swelling is essential to maintain structural integrity, preventing the polymer from becoming too soft or disintegrating, which could weaken mucoadhesion	[84, 85]
Stereochemistry of polymer	Refers to the spatial arrangement of atoms in a polymer, influencing its alignment and interaction with biological structures	**•** It influences mucoadhesion through geometric and chemical complementarity, affecting how polymers align and interact with mucin fibers **•** Specific configurations enhance hydrogen bonding and non-covalent interactions, improving adhesion strength, while misaligned structures may reduce bonding efficiency	[86, 87]
Flexibility of polymer	Refers to the ease with which polymer chains can move and conform to mucosal surfaces, influenced by chemical structure and cross-linking density	**•** Greater polymer chain flexibility facilitates better conformation and penetration into mucosal layers, enhancing mechanical entanglement and chemical bonding for stronger mucoadhesion **•** Excessive flexibility may reduce mechanical integrity, potentially compromising bond durability and long-term adhesion performance	[88, 89]
Mucin turnover rate	Refers to the rate at which mucin is secreted by mucosal glands and removed through degradation or shedding	**•** In conditions like ocular inflammation, accelerated mucin turnover and tear instability reduce mucoadhesive bond duration, challenging sustained drug delivery **•** Requires mucoadhesive polymers to quickly establish strong bonds for effectiveness	[90, 91]
pH at the site of application	Refers to the microenvironmental pH that affects the ionization states of both the polymer and the mucosal surface	**•** pH influences the charge profile of both polymers and mucin, directly impacting electrostatic interactions that are crucial for mucoadhesion **•** pH-sensitive polymers can be tailored to enhance adhesion by optimizing hydrogen bonding and electrostatic interactions under specific mucosal pH conditions, especially in disease-altered environments	[92, 93]

### Potential of nanofiber technology

Nanofibers are fibrous structures with diameters typically below 100 nm, though electrospun fibers in practice often range from 100 to 1000 nm. Despite this, the electrospinning community commonly classifies fibers below 1 µm as nanofibers due to their high aspect ratio and nanoscale effects. These fibers are particularly useful in drug delivery and biomedical contexts, where they can be crafted from either natural or synthetic polymers. Due to their high aspect ratio (length to diameter) and large surface area-to-volume ratio, nanofibers can be tailored to possess distinct physical and chemical properties, allowing drugs/biomolecules to be loaded within their matrix [94]. This customization is often achieved by modifying the polymer composition, molecular weight, crosslinking density, or surface chemistry to tailor mechanical strength, degradation rate, and mucoadhesive properties for specific biomedical applications. Key applications of nanofibers include controlled drug release [95–97], tissue engineering [98–100], wound healing [101–103], and biosensing [104–106].

Nanofibers present unique structures that can facilitate drug delivery to both local and systemic sites. Various manufacturing methods have been utilized to create nanofibers suitable for drug delivery applications. Among the most prevalent methods is electrospinning, along with self-assembly and phase separation [107]. Electrospinning stands out for its ability to generate nanofibers with precise control over their diameter and structure, utilizing a charge-driven process. This technique can produce a range of fiber arrangements such as non-woven, aligned, patterned, randomly distributed, and convoluted dimensions [108]. The core process of electrospinning involves high electrostatic forces that counteract the surface tension of a viscous polymeric solution at the tip of the nozzle. This results in the formation of a charged droplet that extends into a Taylor cone, creating ultra-thin fibers that are collected on a charged substrate. Nanofibers fabricated through electrospinning can be modified pre- or post-production to incorporate drugs/biomolecules [109].

Effective drug uptake via transmucosal delivery is hindered by several inherent challenges, including the presence of keratinized tissues, low patient compliance, and variable drug absorption areas [110]. Moreover, the efficacy of a drug's in vivo performance can significantly depend on its therapeutic window and specific physicochemical properties. The mucus-lined cellular barrier further defines the transmucosal interface, highlighting the importance of drug residence time at the absorption site for optimal therapeutic outcomes [111, 112]. Nanofibers inherently exhibit mucoadhesive properties due to their high surface-to-volume ratios and nonwoven, interconnected structures, which provide extensive surface areas for enhanced mucosal interactions. Specifically, finer nanofibers amplify the specific surface area available for contact with mucosal surfaces [113]. Electrospinning, a charge-driven fabrication technique, further augments this innate mucoadhesivity by depositing charges on the fiber surfaces. These charges foster electrostatic interactions with anionic mucin threads, significantly bolstering the mucoadhesive capabilities of nanofibers [114].

Electrospinning relies on the rapid evaporation of the solvent during fiber formation, which can trap drugs in an amorphous state rather than their crystalline form. This transformation enhances solubility and may improve drug bioavailability [115]. This technique is particularly beneficial for drugs with poor water solubility, as it enables the incorporation of hydrophobic drugs into nanoscale fibers using amphiphilic polymers like poly(vinyl alcohol) (PVA) and polyvinyl pyrrolidone (PVP), which improve drug dispersion and solubilization. Additionally, for hydrophilic drugs, PVA nanofibers enhance membrane wettability and increase bound water content, facilitating their paracellular transport across mucosal barriers [116]. Studies indicate that drug delivery through nanofibers offers more consistent release compared to the pure drug, which shows fluctuating levels [117–121]. This suggests that nanofiber-based delivery systems are particularly advantageous for drugs categorized under the Biopharmaceutics Classification System (BCS) class IV, which struggle with both solubility and permeability challenges [122]. In the context of ocular drug delivery, examples include brinzolamide (for glaucoma) [123], natamycin (for fungal keratitis) [124], and acyclovir (for viral keratitis) [125], all of which suffer from poor aqueous solubility and limited corneal penetration.

Although nanofibers can deliver drugs almost instantaneously for immediate release, this rapid release often leads to the drug being quickly washed out from mucosal surfaces [126]. To manage drug release kinetics effectively, various polymer combinations and modified electrospinning techniques, such as core–shell electrospinning, are utilized [127]. Beyond polymer selection, factors such as fiber diameter, porosity, and surface topography significantly influence drug diffusion rates and retention at the application site [128, 129]. Additionally, post-processing modifications, including crosslinking and surface functionalization, can further regulate drug release profiles by enhancing structural stability or introducing stimuli-responsive properties. While drug loading efficiency is a crucial parameter, it does not solely determine release behavior. High encapsulation efficiency in electrospun nanofibers minimizes wastage and enhances cost-effectiveness, but controlled drug release is more dependent on polymer-drug interactions, the presence of diffusion barriers, and degradation rates of the carrier material [130]. By optimizing the excipient-to-drug ratio, alongside structural modifications, it is possible to fine-tune release kinetics for sustained therapeutic effects while ensuring economic viability for clinical use [131].

## Designing mucoadhesive nanofibers

### Fundamentals of electrospinning

**Fig. 3:**
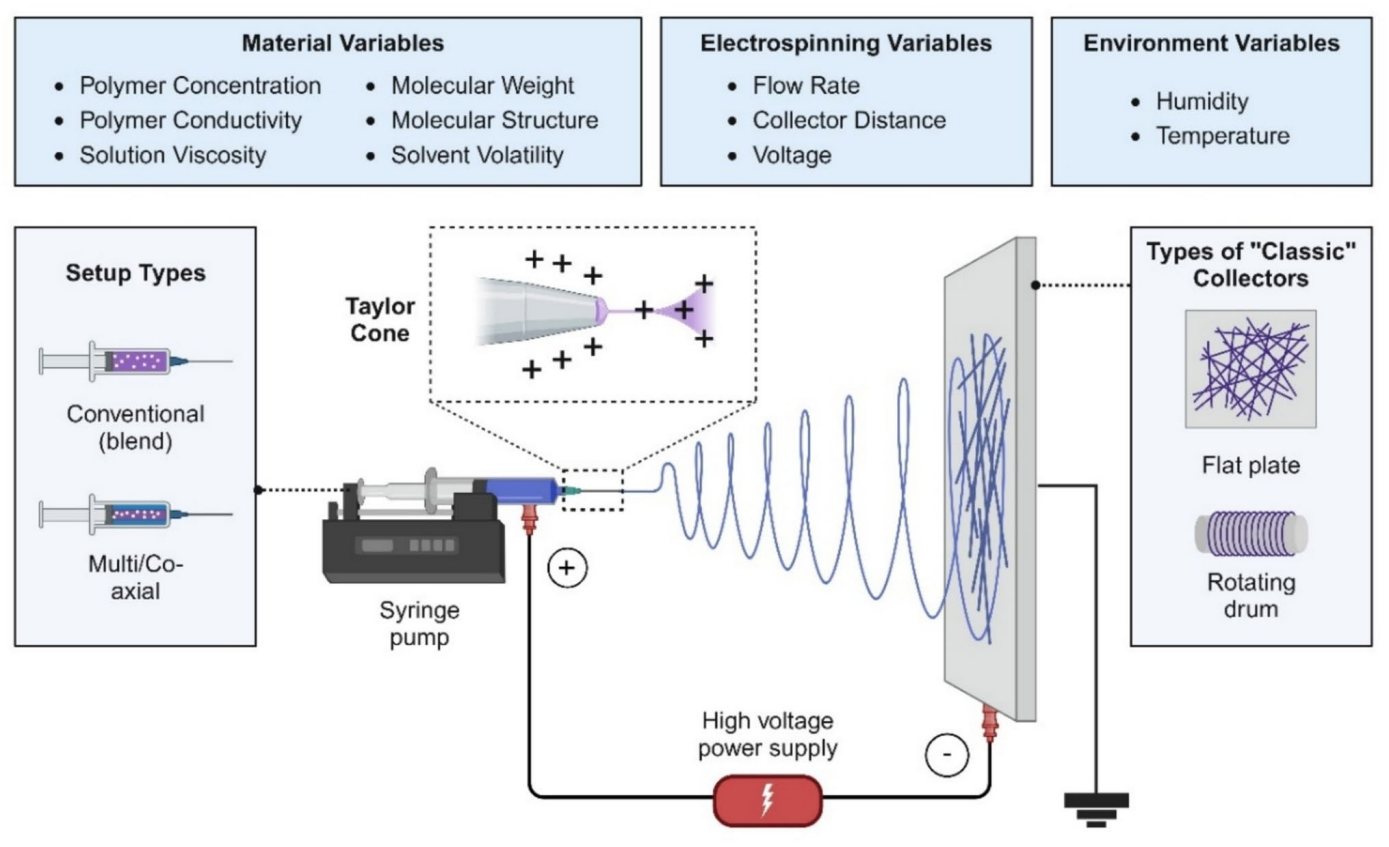
Schematic representation of an electrospinning setup highlighting critical components and variables that affect nanofiber properties. *Created with BioRender.com*

Electrospinning is extensively documented, with numerous high-quality reviews exploring its core principles, adjustable parameters, suitable materials, and fiber collection techniques [132–136]. Briefly, it is a versatile technique that utilizes electrostatic forces to produce fine fibers from a polymeric solution [137]. When a high-voltage field, typically ranging between 5 and 30 kV, is applied, the liquid becomes charged, and electrostatic repulsion overcomes surface tension, forming a Taylor cone at the needle tip, from which a thin polymer jet is ejected [138, 139]. This jet undergoes stretching and thinning as it travels towards the collector, influenced by varicose (axisymmetric) and sinuous (non-axisymmetric) instabilities, particularly the Rayleigh-Plateau instability, which minimizes fiber diameter and enhances uniformity [140]. Solvent evaporation refines fiber morphology, with factors like temperature, humidity, and solvent properties affecting the final fiber structure and mechanical properties [141–143]. A standard electrospinning system includes several key components that dictate fiber formation (Fig. 3). A syringe pump controls the polymer flow rate, typically within 0.1 to 2 mL/h, ensuring uniform fiber deposition [144]. The needle or spinneret, with diameters ranging from 0.1 to 1.0 mm, serves as the exit point for the charged polymer jet, and the needle-to-collector distance, usually set between 10 and 30 cm, allows sufficient time for solvent evaporation to prevent fiber fusion [145]. The collector design, whether a stationary plate or a rotating drum, determines fiber alignment and structural organization. Environmental control units regulate conditions to maintain jet stability, and safety measures mitigate risks associated with high-voltage equipment. By optimizing these parameters, electrospinning enables the fabrication of mucoadhesive nanofibers for ocular drug delivery, where fiber adhesion and controlled drug release are critical for therapeutic efficacy [146–150]. It is worth noting that electrospinning can also produce micro-sized fibers depending on solution properties and processing conditions. These microfibers often share structural and functional similarities with nanofibers. However, this review specifically focuses on sub-micron electrospun fibers, given their enhanced interaction with mucosal tissues.

Understanding the relationship between various electrospinning parameters and their effects on the fabrication process is crucial for optimizing the characteristics of the resultant nanofibers. Each parameter, from voltage settings to environmental conditions, plays a specific role in defining the nanofibers'morphology, uniformity, and structural integrity. Accurate control and adjustment of these parameters enable the tailored design of nanofibers for their use as a drug delivery platform. Table 3 summarizes the key parameters and their impacts on electrospinning and nanofiber properties.

**Table 3:** Electrospinning parameters and their effects on nanofiber formation and attributes

Parameter	Impact on Electrospinning Process	Impact on Nanofiber Characteristics	Ref
Electrical field (voltage)	Sufficient voltage is essential for jet initiation. Higher voltages enhance polymer jet stretching and solvent evaporation, but overly high values disrupt Taylor cone stability	Produces finer fibers with greater stretching. Excessive voltage can cause jet instability and bead formation	[151, 152]
Polymer concentration	Influences solution viscosity. Low concentrations result in weak chain entanglement; high concentrations may clog the needle	Optimal concentrations yield uniform, smooth fibers. Too low causes beading; too high impedes continuous fiber formation	[153, 154]
Flow rate	Regulates solution delivery. High flow can overwhelm the electric field, causing jet instability or ribbon-like structures. Low flow may lead to clogging or jet disruption	Stable flow ensures uniform fiber diameter and smooth morphology. Poor control leads to defects and inconsistent thickness	[155, 156]
Needle-to-collector distance	Affects the time available for solvent evaporation. Improper distances hinder fiber solidification	Proper distance allows fibers to fully dry and solidify, influencing the uniformity and mechanical strength of the fibers. Too short a distance leads to wet, merged/fused fibers; too long a distance may cause fibers to break mid-air	[157, 158]
Solution conductivity and solvent	Higher conductivity improves charge transfer and jet elongation. Solvent properties influence viscosity and evaporation rate	Improved conductivity results in more uniform fibers and reduces beading. The solvent choice should ensure optimal drying times to prevent surface defects	[159, 160]
Environmental parameters	Temperature affects the viscosity of the polymer solution, while humidity influences the solvent evaporation rate	Higher temperatures reduce viscosity, yielding finer fibers. High humidity causes beading; low humidity can result in brittleness	[161, 162]

### Advances in electrospinning: nanofiber composites and multiaxial setup

**Fig. 4:**
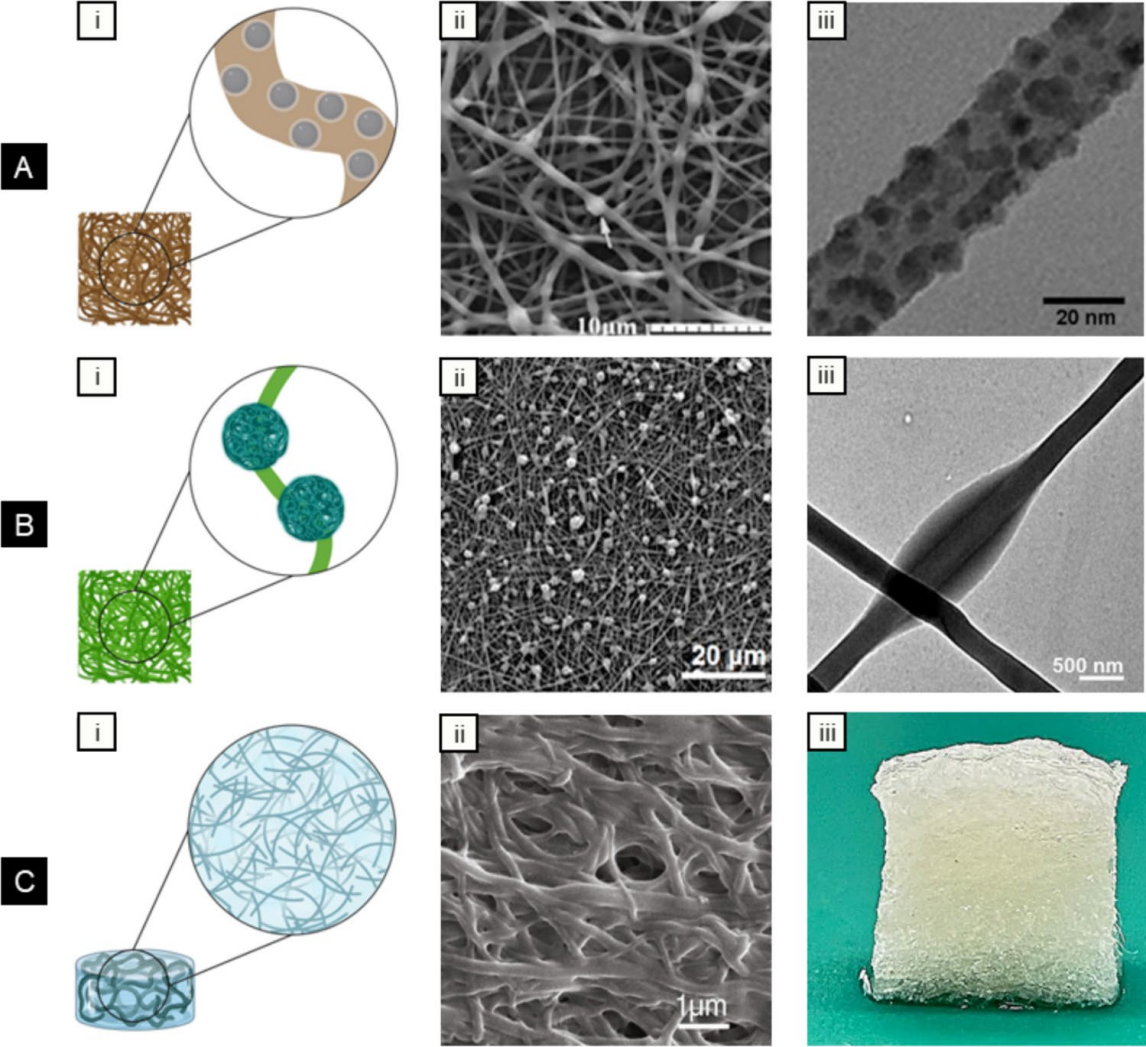
Types of electrospun composites. **A** Nanofibers with nanoparticles. (i) Schematic of nanoparticle-loaded fibers; (ii) SEM of nanofibers with drug-loaded chitosan nanoparticles. *Reproduced with permission from *[177]*, Copyright Springer Nature, 2011.* (iii) TEM of silver nanoparticle-embedded fibers. *Reproduced with permission from *[178]*, Copyright American Chemical Society, 2008.* **B** Nanofibers with microparticles. (i) Schematic of microparticle-loaded nanofibers. (ii) SEM image of nanofiber/microparticle hybrid composite. *Reproduced with permission from *[167]*, Copyright Frontiers Media S.A, 2023.* (iii) TEM of a drug loaded nanofiber/microparticle hybrid composite prepared via coaxial electrospinning. *Reproduced with permission from *[179]*, Copyright Springer Nature, 2022.* **C** Nanofiber-hydrogel composites. (i) Schematic of nanofiber-loaded hydrogel composite. (ii) SEM of a gelatin nanofiber-reinforced hydrogel composite. *Reproduced with permission from *[180]*, Copyright Elsevier, 2017.* (iii) Composite scaffold integrating nanofibers and 3D-printed hydrogel. *Reproduced with permission from *[181]*, Copyright Springer Nature, 2024. All original images created with BioRender.com*

There has been growing interest in developing nanofiber-based hybrid composites, advanced platforms where electrospun nanofibers, inherently capable of drug delivery, are paired with secondary drug delivery systems to precisely tune release profiles and achieve additional therapeutic benefits **(**Fig. 4**)**. This approach enhances the properties of nanofibers by combining them with materials that introduce new functionalities, making them valuable in biomedical applications [163]. In these composites, electrospun nanofibers often serve as the reinforcing phase, providing mechanical strength and structural integrity, while the secondary phase, such as hydrogels [164–166], microparticles [167–169], nanoparticles [170–172], or carbon-based materials like graphene and carbon nanotubes [173–175], offers additional drug-loading capacity, controlled release, or responsiveness to external stimuli. Among these, nanoparticles are the most commonly used due to their small size, which facilitates seamless integration into the polymeric matrices utilized in electrospinning. This combination may improve the mechanical and structural properties of nanofibers and introduces new features, such as responsiveness to magnetic, optical, or thermal stimuli [176].

**Fig. 5:**
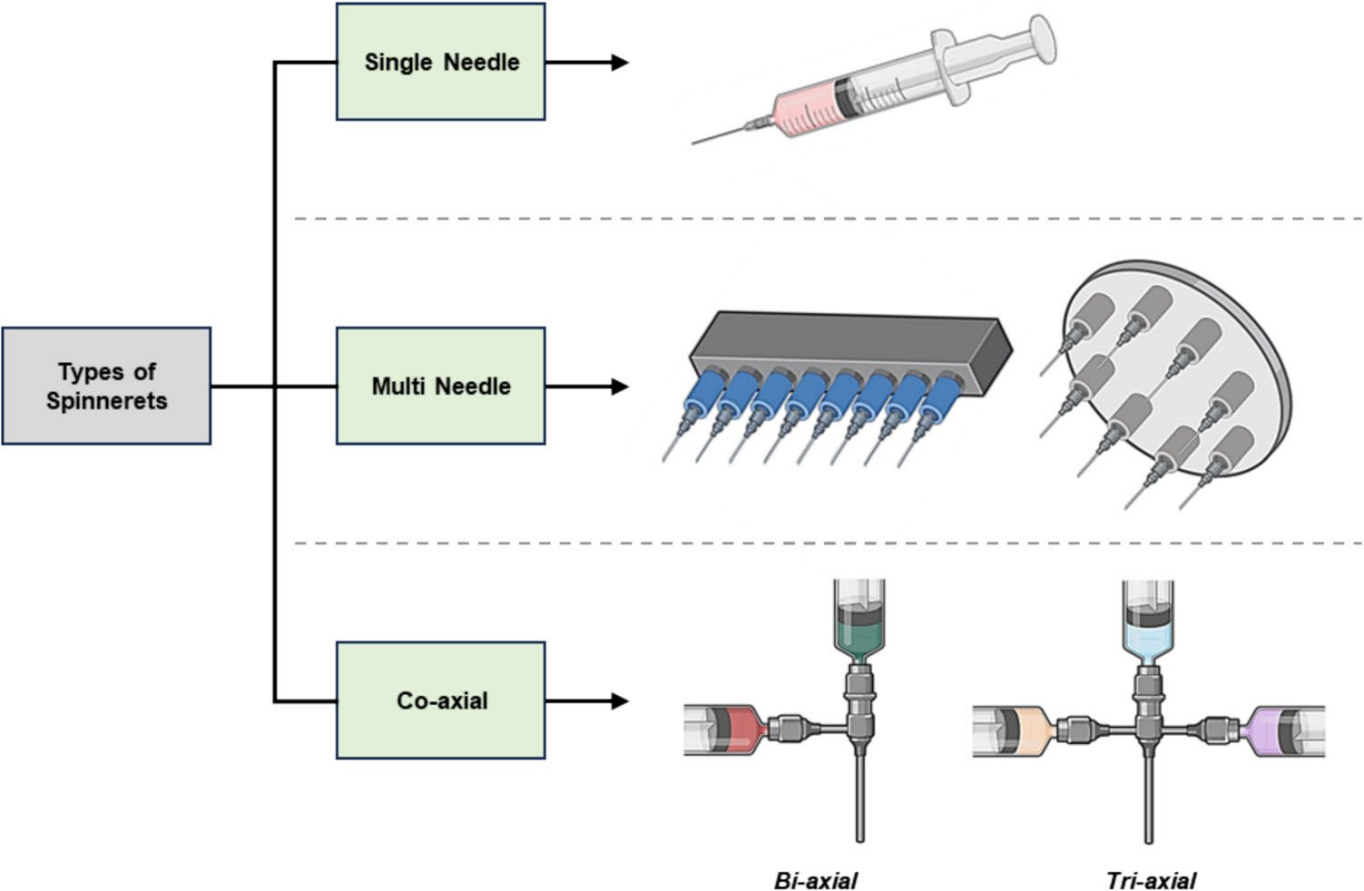
Schematic representation of different types of spinnerets used in electrospinning. Single-needle spinnerets (top) are commonly used for producing uniform nanofibers. Multi-needle spinnerets (middle) enable high-throughput fiber production. Coaxial spinnerets (bottom) facilitate core–shell fiber fabrication, with bi-axial and tri-axial configurations allowing for advanced structural designs for drug delivery applications. *Created with BioRender.com*

Multiaxial electrospinning represents a positive evolution in electrospinning technology, enabling the fabrication of complex nanofiber-based composites without the need for secondary drug delivery phases. This method overcomes the limitations of traditional single-needle electrospinning and coaxial electrospinning by allowing the fabrication of fibers with multiple concentric layers, each capable of incorporating distinct materials tailored for mucoadhesive applications. The typical setup for multiaxial electrospinning includes a multi-needle or multi-channel spinneret, where each channel delivers a different polymer solution, facilitating precise layering and material distribution **(**Fig. 5**)**. By carefully designing layer composition and thickness, multiaxial electrospinning can create protective barriers around biomolecules, safeguarding them from enzymatic degradation and shear forces present in mucosal environments [182]. Additionally, the outermost layers can be engineered using bioadhesive polymers, enhancing their interaction with mucins and prolonging retention at the target site [183]. The ability to incorporate hydrophilic and hydrophobic layers enables better drug encapsulation and sustained release, which is particularly beneficial for mucoadhesive systems aimed at localized and prolonged therapeutic effects. The release dynamics of active agents can be finely controlled through the manipulation of layer permeability and thickness. For instance, a thicker and denser outer layer may slow diffusion, promoting a prolonged release, while a thinner or more porous layer can accelerate the release [184, 185]. Moreover, stimuli-responsive polymers can be selectively introduced into designated layers, allowing the fibers to alter their properties in response to pH variations, ionic strength, or enzymatic activity within the mucosal environment. This adaptability can further optimize drug release kinetics while maintaining adhesion to biological surfaces [186].

### Strategies to incorporate mucoadhesive features

This section explores a range of techniques aimed at enhancing the mucoadhesive properties of nanofibers specifically for ocular drug delivery applications. Although some of these strategies have not yet been directly applied or reported in the context of ocular nanofibers, they have shown promise in other types of biomaterial systems. The core scientific principles underlying these methods remain broadly applicable and suggest strong potential for translation to nanofiber platforms, providing a foundation for future innovations in ocular drug delivery.

#### Polymer blending

Polymer blending is a versatile and cost-effective strategy for developing materials with tailored properties. This approach combines two or more polymers to create composites with functionalities not achievable by individual components [187]. One common method involves physically mixing polymers in the molten state using equipment such as extruders or melt-compounders, where miscibility and phase behavior determine blend homogeneity [188, 189]. Thermal properties such as glass transition and melting temperatures depend on the characteristics and interactions of the component polymers [190]. However, many polymer pairs are immiscible, resulting in phase-separated structures that require compatibilizers to enhance interfacial adhesion and improve mechanical properties [191–193]. While melt blending is traditionally used in thermoplastic processing, it can also serve as a preliminary step for creating uniform polymer blends that are subsequently dissolved in a suitable solvent for electrospinning. In electrospinning, direct solution blending is more typical, especially for temperature-sensitive or bioactive compounds [194, 195]. The resulting spinnable solution's composition, including polymer ratios and solvent compatibility, directly influences nanofiber morphology, drug encapsulation, and in vivo performance.

In mucoadhesive drug delivery systems, the combination of bioadhesive agents with synthetic polymers is especially advantageous. Synthetic polymers like poly(lactic-co-glycolic acid) (PLGA) and poly(ε-caprolactone) (PCL) are preferred for electrospinning due to their processability and mechanical strength but typically lack effective mucoadhesion, essential for successful transmucosal drug delivery. In contrast, natural bioadhesive materials such as chitosan and hyaluronic acid excel in mucoadhesion but often face challenges in electrospinning related to solubility and drug-loading capabilities [196]. By blending these two polymer types, the goal is to leverage the mucoadhesive qualities of natural materials alongside the favorable processing attributes of synthetic polymers, thus enhancing the mucoadhesion of the resulting nanofibers [197, 198].

Table 4 provides a brief overview of bioadhesive agents that can be incorporated into the polymer matrix to enhance the adhesion of nanofiber formulations to mucosal tissues. The intermolecular interactions between the polymers increase both the mechanical strength and the adhesiveness of the nanofibers to mucosal tissues, ensuring a more robust and durable attachment. This is crucial for prolonging the mucoadhesive effect and achieving sustained therapeutic agent release [199]. Furthermore, the use of natural polymers typically increases the hydrophilicity of the blend, which is favorable for maintaining moisture at the mucosal surface. This enhanced hydration improves adhesion by promoting interactions between the nanofibers and the mucosal tissues, thus enhancing the comfort and effectiveness of the drug delivery system.

**Table 4:** Bioadhesive agents and their properties

Bioadhesive Agent	Key Properties	Ref
Chitosan	Cationic biopolymer derived from chitin; possesses strong mucoadhesivity due to electrostatic interactions with negatively charged mucosal surfaces; enhances penetration through tight junctions. Also, it is biodegradable and has antimicrobial properties	[200–202]
Hyaluronic acid	Naturally occurring glycosaminoglycan with high viscoelasticity; promotes prolonged retention on mucosal surfaces through receptor-mediated (CD44) adhesion; excellent biocompatibility and promotes cell proliferation	[203–205]
Thiomers	Modified polymers with pendant thiol groups; form strong disulfide bonds with cysteine-rich mucins, increasing mucolytic degradation resistance and improving the mucoadhesive bond's stability	[206–208]
Alginate	Anionic polysaccharide that forms hydrogels in the presence of calcium ions; its gel formation at physiological pH and ionic strength mimics the natural mucus, providing sustained adhesion and compatibility	[209–211]
Cellulose derivatives	It includes hydroxypropyl cellulose and carboxymethyl cellulose (CMC) and exhibits excellent water-holding capacity and film-forming ability, facilitating extended mucoadhesion through hydrogen bonding and mechanical interlocking	[212–214]
Pectin	Plant-derived polysaccharides that gel in the presence of divalent cations form a bioadhesive barrier sensitive to pH changes, which can be exploited for targeted drug release	[215–217]
Carbopol	Cross-linked polyacrylic acid that can absorb and retain large amounts of water, forming thick gels; these gels significantly increase the residence time on mucosal surfaces, enhancing sustained drug release	[218–220]
Polyvinyl alcohol (PVA)	Synthetic polymer notable for its high hydrophilicity and excellent film-forming abilities; the formation of a hydrogel layer on mucosal surfaces facilitates strong mucoadhesion through physical entanglements	[221–223]
Polyvinylpyrrolidone (PVP)	Water-soluble polymer with good adhesion and film-forming properties; forms a non-ionic bond with the mucosal surface, making it suitable for sensitive mucosal applications where ionic interactions are undesirable	[224–226]

The proportion (relative ratio) of each component in the blend is a vital parameter that must be carefully optimized to meet predetermined requirements for biocompatibility, degradation properties, mechanical strength, and drug release kinetics. The optimal blending ratio affects the nanofibers’ structural integrity, chemical interactions, and overall efficacy [227–229]. For instance, Brako et al. demonstrated that blending polyethylene oxide (PEO) with increasing amounts of CMC significantly enhanced the mucoadhesive properties of progesterone-loaded nanofibers, as measured by both texture analysis and atomic force microscopy (AFM) [230]. Specifically, higher CMC content led to stronger mucoadhesive interactions and smoother fiber–mucosa interfaces, confirming the positive correlation between blend composition and mucoadhesion. Similarly, in another study, nanofibers prepared from blends of PEO with various mucoadhesive polymers such as sodium alginate and polyacrylic acid showed that the incorporation of 25 wt% CMC or alginate resulted in fibers with superior mucoadhesive potential compared to those composed solely of synthetic polymers [231].

It should be noted that an excess of synthetic polymer might yield fibers with excellent mechanical properties but insufficient mucoadhesion and biodegradability. Conversely, excess bioadhesive agents can improve mucoadhesion but at the expense of structural and processing qualities [232]. Achieving the ideal balance demands extensive experimental efforts involving iterative adjustments and testing to tailor the fibers’ performance to therapeutic objectives.

#### Surface modification

Surface modification of nanofibers encompasses a variety of techniques designed to alter the surface attributes of the fibers post-production, thereby improving their functionality. This process targets only the exterior layer of the nanofibers, ensuring that their core structural properties remain unchanged while introducing functional groups or structural modifications that improve adhesion to mucosal tissues [233, 234]. These modifications can generally be categorized into two main types: physical and chemical methods, both of which play a significant role in enhancing the mucoadhesive potential of nanofiber-based drug delivery systems.

#### Physical treatments

Physical surface modification techniques, such as plasma treatment, corona discharge, and UV irradiation, alter nanofiber properties without chemical additives, expanding surface area or introducing functional groups via physical interactions. These methods enhance mucoadhesion by increasing surface roughness, hydrophilicity, or mucin-binding functional groups, and are adaptable to various polymers with precise control over modification. However, scaling these processes industrially remains challenging due to difficulties in ensuring uniform treatment across large volumes [235].

- **Plasma treatment:** This technique employs ionized gas to induce physicochemical changes in nanofibers via high-energy interactions. The process cleaves molecular bonds, introducing functional groups (e.g., carboxyl, hydroxyl, amine) that enhance mucoadhesion through hydrogen bonding and electrostatic interactions with mucins [236]. Additionally, plasma exposure increases surface reactivity, hydrophilicity, and energy, improving adhesion properties [237]. For example, Das et al. found that dielectric barrier discharge plasma treatment of electrospun PVA/chitosan nanofibers significantly reduced water contact angles and increased polar surface energy, leading to enhanced wettability and cell compatibility, which are crucial for mucoadhesion [238]. The high-energy species can also etch the surface, increasing roughness and facilitating physical interlocking with mucosal surfaces, further enhancing mucoadhesive potential [239]. In a related study, Li et al. reported that composites based on electrospun PCL membrane achieved prolonged gastric retention and robust mucoadhesive strength, supporting the role of plasma-processed electrospun membranes in optimizing drug delivery to mucosal tissues [240]. Additionally, plasma polymerization, a process where plasma polymerizes gaseous monomers (e.g., acrylic acid) to deposit thin functional coatings, can be used to tailor nanofibers’ mucoadhesive properties [241, 242].
- **Corona discharge:** The principle of corona discharge relies on the high electric field gradient at sharp edges or points, which exceeds the air's dielectric breakdown strength. As a result, electrons are accelerated to high energies and ionize the air molecules, leading to the formation of ions, free electrons, and other reactive species. These reactive species, including ozone and various radicals, interact with the surface of the nanofibers placed near the corona discharge. This interaction alters the surface properties of the nanofibers by introducing polar functional groups that increase the surface energy and hydrophilicity of the nanofibers, enhancing their adhesive properties and compatibility [243]. Detailed discussions on the mechanisms and broader bioadhesive applications of corona discharge are available in other sources [244, 245]. Importantly, beyond conventional surface treatment, corona discharge can be incorporated directly into the electrospinning process to generate nanofibers with tailored porosity and surface characteristics, which can support improved mucoadhesion. For example, Song et al. demonstrated that corona-assisted electrospinning produced three-dimensional nanofiber matrices with features that facilitate better cell interaction and tissue integration, attributes that are equally valuable for maximizing mucoadhesive performance in drug delivery systems [246].
- **Ultraviolet (UV) irradiation:** This process exposes nanofibers to high-energy photons, breaking chemical bonds within the polymer matrix and generating reactive radicals on the surface [247]. These radicals may form new functional groups or trigger cross-linking, enhancing mucoadhesion through covalent and non-covalent mucin binding [248]. Additionally, UV-induced cross-linking can delay polymer degradation, enabling sustained drug release at mucosal sites. While UV irradiation has not been widely studied for ocular mucoadhesion, Rabiatul et al. found that UV-treated nanofibers had a lower water contact angle and greater cell attachment, both indicative of improved adhesive properties [249].

#### Chemical treatments

Chemical surface modification enables precise tailoring of nanofibers to enhance molecular recognition and adhesion to mucosal membranes. These treatments improve durability, reactivity, and material interactions, optimizing drug delivery efficiency. However, the use of harsh chemicals or solvents raises environmental and safety concerns, necessitating careful handling and disposal, which may limit broader applicability [250].

- **Grafting:** This method attaches polymeric chains or functional groups to nanofiber surfaces, enhancing functionality via"grafting to"and"grafting from"methods. For mucoadhesion, polymers like chitosan, poly(acrylic acid), or thiolated polymers are grafted to strengthen hydrogen bonding and covalent mucin interactions [251, 252]."Grafting to"bonds pre-synthesized chains to active sites, offering precise control over molecule orientation and density, though steric hindrance can limit high-density grafting [253, 254]. For example, chitosan grafted onto electrospun poly(DL-lactide) fibers increased surface wettability and cell proliferation [255]. Caffeic acid grafted to poly(L-lactic acid) nanofibers also improved hydrophilicity and cell attachment, both of which support mucoadhesive performance [256]. In contrast,"grafting from"initiates polymerization from surface-bound groups, forming dense polymer layers that can enhance barrier and adhesive properties, although excessive growth may affect porosity [257]. For instance, surface-initiated polymerization grafting of poly(ethylene glycol) methacrylate from electrospun polycarbonate urethane fibers significantly increased hydrophilicity and promoted cell adhesion [258]. Similarly, photografting of 2-hydroxyethyl acrylate onto electrospun poly(ethylene-co-vinyl alcohol) mats improved wetting characteristics and enhanced overall surface compatibility for mucoadhesive applications [259].
- **Etching:** It selectively removes surface layers of nanofibers using chemical reagents, resulting in increased roughness, porosity, and availability of reactive sites [260]. By dissolving surface components and breaking down polymer chains or amorphous regions, this process optimizes the surface topology, promoting greater mechanical interlocking and more effective interactions with mucosal tissues for enhanced mucoadhesion [261]. Experimental evidence shows that sodium hydroxide-treated electrospun poly(ε-caprolactone) membranes display markedly higher surface roughness and a dramatic reduction in water contact angle, leading to improved cell attachment and spreading. The resulting nanoscale features and increased wettability provide additional binding sites, further strengthening adhesion to biological surfaces [262]. Despite its potential, this approach remains relatively underexplored for ocular applications.
- **Layer-by-layer (LbL) assembly:** It enables precise deposition of multilayered coatings on nanofibers, utilizing electrostatic attraction between charged entities such as polyelectrolytes, nanoparticles, and proteins [263]. While primarily driven by electrostatic interactions, hydrogen bonding, van der Waals forces, and covalent bonding also contribute to layer stability [264]. The process alternates material deposition via dipping, spraying, or spin-coating, progressively building layers with tunable properties [265]. By selecting specific mucoadhesive polymers, LbL assembly enhances nanofiber interactions with mucosal tissues, improving adhesion [266]. Moreover, this method allows for controlled drug encapsulation within the layered structure, optimizing targeted delivery [267, 268]. For example, Müller et al. demonstrated that polyelectrolyte multilayers of poly(allylamine hydrochloride) and poly(styrenesulfonate) can be deposited onto electrospun fiber surfaces to tailor hydrophilicity, surface charge, and potentially bioadhesive properties [269]. Similarly, Chunder et al. showed that pH-responsive and temperature-sensitive multilayers, such as poly(acrylic acid)/poly(N-isopropylacrylamide), can be built onto electrospun fibers via LbL assembly to provide tunable release and controlled surface functionality, both important for achieving enhanced mucoadhesion and drug delivery performance [270].

#### Molecular imprinting

Molecular imprinting is an emerging technique that seeks to create highly specific binding sites within a cross-linked polymer matrix. The theoretical goal is to achieve binding sites that match the size, three-dimensional structure, and chemical functionality of a target"template"molecule, in a manner similar to the selective binding seen with antibodies and antigens [271]. In principle, this method could be applied to functionalize nanofiber surfaces in order to achieve selective and robust adhesion to mucosal tissues. The general process involves polymerizing monomers that are chosen for their potential to form covalent or non-covalent interactions with the template, in the presence of cross-linkers, which results in a rigid polymer network formed around the template. There is growing interest in the possibility that adding bioadhesive ligands, mucin-mimicking functional groups, or glycoproteins into such matrices could significantly enhance the mucoadhesive properties of nanofibers and help achieve prolonged drug retention at mucosal sites [272]. However, these ideas are still under investigation, and practical validation remains limited.

After polymerization, the extraction of the template is typically performed through careful washing procedures, in an attempt to leave behind the specific binding sites. Alternatively, in solid-phase imprinting, the template may be pre-immobilized on a solid support, such as glass, silicon, or iron oxide, before polymerization. This strategy is expected to simplify template removal or allow for template reuse, and it is also supposed to ensure that bioadhesive functional sites are accessible on the nanofiber surface for optimal mucosal adhesion [273]. Although such imprinted sites have been reported to offer stability and reusability, these claims and the broader utility of molecularly imprinted polymers (MIPs) in drug delivery systems that require extended residence at mucosal interfaces are still being assessed. The main categories of this technique that are currently being explored include the following:

- **Molecular imprinting during electrospinning:** This approach involves incorporating the template molecule directly into the electrospinning solution, with the aim of achieving a uniform distribution of imprinted sites within the resulting nanofibers. However, this method presents several practical challenges. There is a fundamental conflict between the requirements for molecular imprinting, which depend on a stable, cross-linked polymer network to maintain the functionality of the binding sites, and the requirements for electrospinning, which need soluble polymers to produce uniform fibers [274]. The insolubility of cross-linked MIPs generally limits compatibility with electrospinning, but research is ongoing to overcome these obstacles. Some potential solutions under investigation include the use of partially cross-linked polymers, fine-tuning the cross-linking density to balance solubility and structural integrity, or adding solubility-enhancing cross-linkers in an effort to create electrospinnable mixtures that retain imprinting capability [275].

Additionally, it is theorized that functional groups able to interact with mucosal surfaces, such as thiolated polymers or lectin-like structures, could be incorporated into the electrospinning process. This could result in nanofibers that adhere to mucus layers through both molecular recognition and covalent interactions [276, 277]. It is important to note that careful optimization of electrospinning parameters is essential, as electrostatic forces during fiber formation might otherwise disrupt the formation of the intended imprinted sites.

- **MIP layer formation onto nanofibers:** This approach relies on post-processing surface modification techniques, such as dip-coating or layer-by-layer (LbL) assembly, to introduce molecularly imprinted sites without compromising the mechanical stability of the nanofiber scaffold [278, 279]. By separating fiber fabrication from the imprinting step, it is hypothesized that tailored surface functionalization can be achieved while maintaining fiber integrity, which could enhance mucoadhesion. The MIP layer is generally created by polymerizing monomers in the presence of a template molecule directly on the fiber surface. This method is intended to leave the imprinted cavities exposed and accessible for selective interactions with mucins or other biological targets [280, 281].

Researchers are interested in incorporating mucin-mimicking glycoproteins or sialic acid structures by this method, with the aim of improving mucoadhesion, prolonging drug residence time, and enabling controlled drug release in mucosal environments. Following polymerization, the template molecules are typically removed via solvent extraction, which is intended to leave behind highly specific binding sites that match the template’s shape, size, and functional groups [282]. While there is much promise in this approach, consistent practical demonstration of these benefits remains to be seen.

- **Solid-phase imprinting:** In this technique, template molecules are immobilized on a solid substrate, and a monomer mixture is then polymerized around the template to create molecularly imprinted sites within the structure. For mucoadhesive applications, this approach is believed to allow precise positioning of bioadhesive functional groups on nanofibers, which could optimize their interactions with mucosal surfaces [283].

A notable variant of this method uses electrospun fibers as the substrate, either by coating them with a template-incorporated polymer or by integrating the template during electrospinning. This is designed to preserve the fibers’ high surface area while embedding mucin-mimicking structures to achieve targeted and robust bioadhesion [284]. The potential benefits of this approach include compatibility with a wide variety of solvents and polymer-template chemistries, which could broaden the range of mucoadhesive materials and improve control over specific interactions. Improved accessibility of imprinted cavities may also help to support prolonged adhesion and sustained drug release [285].

### Assessment of mucoadhesion

The development of mucoadhesive nanofiber systems depends on pre-clinical tests that quantify their adhesion to mucosal tissues, ensuring targeted drug delivery and sustained release. These tests enable the optimization of polymer composition, surface properties, and formulation parameters before clinical translation, ensuring efficacy and safety [286].

Mucoadhesion can be assessed using either isolated mucin or intact mucosal tissues, depending on the study objectives. When focusing on molecular interactions and binding affinities, nanofibers are typically incubated with purified mucin solutions, whether self-extracted or commercially obtained. Alternatively, studies involving excised or in vitro cultured mucosal surfaces better approximate physiological conditions, enabling direct evaluation of adhesion strength and distribution on biological tissues [287]. Commonly studied tissues for mucoadhesion include buccal, intestinal, and nasal mucosa, while ocular tissues are less frequently investigated due to limited access to physiologically relevant ex vivo or in vitro models. However, the assessment techniques outlined in this section are broadly applicable and can be adapted for ocular scenarios using excised corneal or conjunctival tissues, cultured ocular epithelial cells, or simulated tear film environments. Table 5 summarizes widely used methods for evaluating nanofiber mucoadhesion, including their potential adaptations for ocular applications, where standardized protocols remain limited.

**Table 5:** Test methods for assessing the mucoadhesive properties of nanofibers in vitro

Method	Description	Application	Ref
Nuclear magnetic resonance (NMR) spectroscopy	**•** ^1^H NMR (Proton NMR) is a highly sensitive spectroscopic technique used to analyze the magnetic environment of hydrogen atoms, providing detailed information on proton distribution and molecular structure **•** ^13^C NMR (Carbon-13 NMR) is less sensitive due to the lower natural abundance of ^13^C but offers insights into the carbon skeleton of organic molecules	**•** Proton selective relaxation rate NMR measures binding affinity and interaction dynamics between nanofibers and mucins, highlighting the impact of structural modifications on mucoadhesion **•** Pulsed-gradient spin-echo NMR assesses how these interactions affect nanofiber mobility in mucin solutions, offering insight into mucoadhesive behavior under physiological conditions	[301, 302]
Attenuated total reflectance-Fourier transform infrared spectroscopy (ATR-FTIR)	**•** ATR-FTIR uses infrared light to detect molecular vibrations, revealing detailed information about a sample’s chemical structure and composition **•** It requires minimal sample preparation and is versatile, allowing analysis of solids, liquids, and gels	**•** ATR-FTIR detects specific bonding interactions involved in mucoadhesion by identifying chemical changes when nanofibers contact mucosal surfaces **•** It also evaluates how surface modifications affect nanofiber–mucin interactions and the stability of the adhesive complex	[303, 304]
Confocal laser scanning microscopy (CLSM)	**•** It provides high-resolution, 3D imaging of biological specimens by scanning with a laser and excluding out-of-focus light **•** It enables detailed visualization of cellular structures and molecular interactions within thick tissue sections	**•** This technique enables the high-resolution, three-dimensional imaging of nanofibers within biological tissues, making it possible to observe their interaction with mucosal surfaces in real-time **•** By tagging nanofibers with fluorescent markers, CLSM can be used to trace their distribution and retention on mucosal tissues after administration	[305, 306]
Atomic force microscopy (AFM)	**•** It is a scanning probe technique that offers high-resolution imaging down to the atomic level **•** It measures the force between a sharp probe and the sample surface to analyze topography, material properties, and nanoscale interactions	**•** AFM force spectroscopy quantifies adhesive interactions by measuring force-distance profiles between a nanofiber-tipped probe and mucosal surfaces, revealing adhesion strength and work of adhesion **•** AFM imaging provides visual assessment of nanofiber conformation and distribution on mucosal surfaces before and after contact	[307, 308]
Tensile testing	**•** Texture analysis measures the force needed to pull a sample apart, revealing its tensile strength and elongation behavior **•** It evaluates mechanical properties such as elasticity, deformation resistance, and structural integrity under stress	**•** By attaching nanofiber samples to a probe and immersing them in a mucus mimic, texture analyzers can measure the force required to detach the nanofibers, quantifying their adhesive strength	[309, 310]

Natural mucins are typically extracted from porcine stomach (Type II mucin) or bovine submaxillary glands, reflecting their presence in human mucus but notably not sourced from ocular tissues [288]. In this process, mucosal tissues are mechanically homogenized in a buffer solution, typically containing a protease inhibitor to prevent mucin degradation, and then centrifuged to remove debris. Solvents (e.g., ethanol) or high-salt solutions are added to precipitate mucin from the supernatant, effectively separating it from non-mucin proteins and other impurities [289]. The precipitated mucin is dialyzed against a suitable buffer (often a saline or Tris–HCl buffer) to remove the precipitating agent and any remaining low-molecular-weight contaminants [290]. This step is crucial for achieving the purity required for reliable testing. Finally, the dialyzed mucin is lyophilized, producing a dry powder that can be reconstituted in buffers of various concentrations for mucoadhesion testing [291]. The use of non-ocular mucins in ocular surface models raises important considerations. While porcine and bovine mucins share structural similarities with human mucins, such as high molecular weight and glycosylation patterns, they differ from ocular mucins like MUC5AC and MUC16, which are specifically expressed in the tear film and conjunctival epithelium [292, 293]. These ocular mucins contribute to the tear film’s unique viscosity, hydration, and protective properties, which may not be fully replicated by gastric or submaxillary mucins [294, 295]. For researchers aiming to mimic the ocular surface, this discrepancy could affect mucoadhesion, drug release profiles, and interactions with the tear film, potentially leading to less accurate models of ocular drug delivery or surface protection. However, porcine and bovine mucins can still serve as a reasonable mimic for preliminary studies, as they replicate general mucoadhesive properties and can be more readily sourced and purified in large quantities, offering a practical alternative when ocular-specific mucins are unavailable.

For consistency and ease of use, commercially available purified mucin is frequently employed, offering a standardized alternative to self-extracted preparations [296]. Regardless of the source, it is essential to thoroughly characterize the mucin to confirm its suitability for mucoadhesion tests. Characterization involves determining the protein concentration through assays such as Bradford or BCA protein assays, which provide insights into the purity and concentration of mucin proteins [297]. Rheometric analysis determines the viscoelastic properties of the sample, confirming its ability to replicate the mechanical behavior of natural mucus [298]. Electrophoretic analysis, typically with SDS-PAGE, is employed to verify the molecular weight and purity of the mucin proteins, confirming that the extraction and purification processes have not degraded the essential properties of mucin [299]. Furthermore, gel permeation chromatography is used to assess the molecular size distribution, ensuring the consistency and uniformity of the mucin preparation [300]. Together, these characterization techniques ensure that the mucin used in mucoadhesion tests is reliable, reproducible, and biologically relevant, providing a solid foundation for the testing of mucoadhesive nanofiber formulations.

## State-of-the-art applications

This section examines recent advancements in mucoadhesive nanofibers for ocular drug delivery, focusing exclusively on studies published after 2021 to reflect the latest developments in the field. These investigations harness nanofiber technology to target a range of ocular conditions, including glaucoma, bacterial and fungal infections, and corneal wounds, showcasing its adaptability across diverse therapeutic contexts. Through a critical analysis, we compare these nanofiber-based systems with other biomaterial-based approaches, such as in situ gels, nanoparticles, and hydrogels, to elucidate their distinct benefits, including extended drug release profiles, enhanced bioavailability, and potential for improved patient adherence. Where relevant, we identify critical challenges, such as scalability of production, mechanical constraints under physiological conditions, and the need for adjustable release kinetics to balance rapid onset with sustained delivery. By critically assessing these developments, we aim to underscore the transformative potential of mucoadhesive nanofibers while pinpointing opportunities for future refinement to drive innovation and support clinical translation.

### Delivery of antimicrobials

**Fig. 6:**
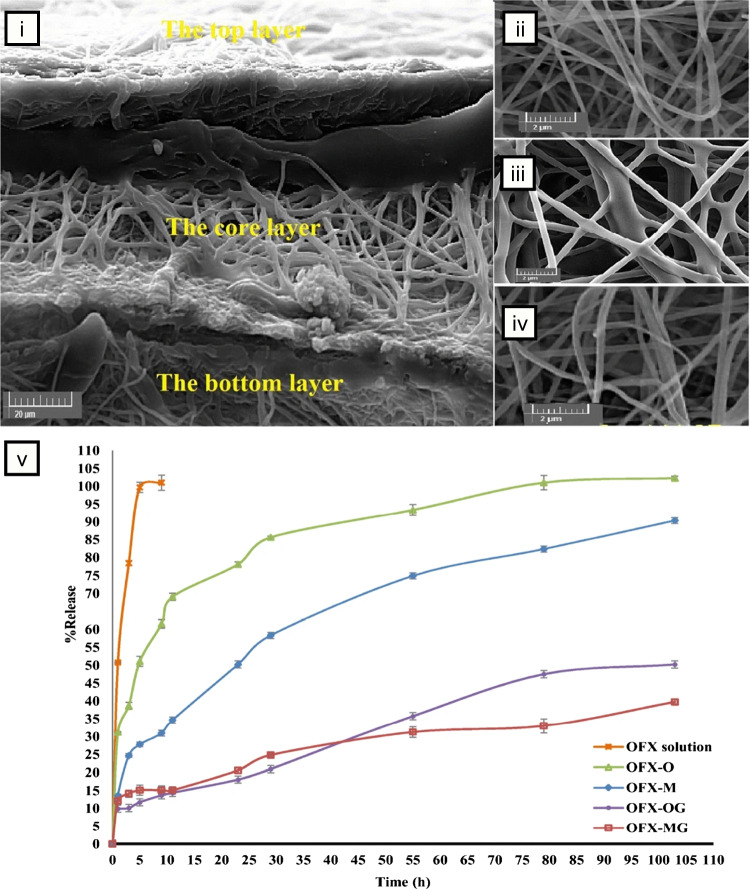
Multi-layered electrospun nanofibers as an ocular matrix for the controlled Ofloxacin release. (i) SEM cross-section of multi-layered electrospun nanofibrous structures after glutaraldehyde cross-linking (OFX-MG formulation), with magnified areas of top Eudragit RL100 layer (ii), CS-PVA-OFX at the core (iii), and bottom Eudragit RL100 layer (iv). (v) In vitro cumulative release behavior of ofloxacin from the various formulations. *Reproduced with permission from* [311]*, Copyright Springer Nature, 2021*

Ofloxacin (OFX) is a fluoroquinolone antibiotic known for its broad-spectrum antibacterial activity, making it effective against various Gram-positive and Gram-negative organisms. In ophthalmology, it is commonly prescribed to treat bacterial eye infections. Mirzaeei et al. [311] designed mucoadhesive nanofibers for delivery of OFX, leveraging the inherent bioadhesive properties of chitosan (CS) to tackle short residence times in treating bacterial conjunctivitis. Their approach utilized electrospun single-layered nanofibers composed of CS and polyvinyl alcohol (PVA), with some formulations further enhanced by multi-layered designs incorporating hydrophobic Eudragit RL100 (a synthetic, pH-independent, cationic copolymer) coatings and glutaraldehyde (GA) cross-linking. The mucoadhesive nature of CS was pivotal in extending drug retention on the corneal surface. This was evidenced by in vivo rabbit studies, where cross-linked multi-layered nanofibers (OFX-MG) maintained OFX concentrations in tear fluid above the minimum inhibitory concentration for an impressive 95 h which far surpasses the 10-h duration of a standard OFX solution. This prolonged retention translated to a 9.23-fold increase in bioavailability (AUC₀₋₉₆) compared to the solution, underscoring the role of mucoadhesion in reducing dosing frequency and enhancing therapeutic efficacy. The addition of Eudragit RL100 layers and GA cross-linking further refined this system by tempering the initial burst release seen in non-cross-linked single-layered fibers (93.8% OFX released in 103 h) to a more controlled 39.82% over the same period, highlighting a synergy between mucoadhesion and structural design (Fig. 6). Notably, the nanofibers’ mechanical robustness, with folding endurance exceeding 200 folds, suggests durability under the dynamic conditions of blinking, a practical advantage for patient comfort. Safety was affirmed by minimal ocular irritation in Draize tests and over 70% cell viability in L929 fibroblast assays, despite minor concerns about residual GA. However, the study stops short of quantifying mucoadhesion strength directly, a gap that limits full appreciation of CS’s contribution relative to structural factors.

While these chitosan nanofibers offer significant advantages, other researchers have explored alternative mucoadhesive systems for ocular drug delivery. For example, Salama et al. [312] developed mucoadhesive ofloxacin-loaded polymeric nanoparticles using polycaprolactone (PCL) and chitosan hydrochloride (CS-HCl). These nanoparticles were integrated into in situ gels (LPCL-NP2-ISG4) and preformed gels (LPCL-NP2-G4), exhibiting sustained drug release and enhanced antimicrobial efficacy in rabbit models. The in situ gel formulation (LPCL-NP2-ISG4) showed superior corneal penetration and prolonged residence time, driven by its mucoadhesive properties and temperature-triggered gelation. However, its release duration was notably shorter than that of the chitosan nanofibers, which sustained ofloxacin release for 103 h. This prolonged profile likely arises from the nanofibers’ fibrous matrix, which provides superior surface-area-to-volume ratios and mechanical interlocking with ocular mucin, outpacing the electrostatic adhesion of the nanoparticles. Such extended delivery could reduce dosing frequency, improving patient compliance, while the nanofibers’ planar structure may ensure more uniform drug distribution, potentially surpassing the irregular penetration seen in LPCL-NP2-ISG4. Similarly, Dey et al. [313] developed a locust bean gum (LBG)-based in situ gel for ocular OFX delivery, employing N-isopropyl acrylamide (NIPAAm) grafting for temperature sensitivity. This gel achieved sustained drug release over 24 h and fully healed bacterial keratitis in rat models. Although it underscored the value of mucoadhesion and temperature sensitivity in enhancing drug retention and efficacy, its release profile (~ 90% over 24 h) was faster than the ~ 40% release over 103 h observed with the nanofibers. This indicates that while in situ gels excel in rapid and sustained release, nanofibers provide a more prolonged and controlled release, making them better suited for chronic conditions requiring long-term drug delivery. Although the nanofiber system shows great promise, future studies should investigate incorporating burst-release mechanisms to address acute infections while preserving sustained release for chronic conditions. Further research is also needed to assess the safety and efficacy of these nanofibers in human subjects.

**Fig. 7:**
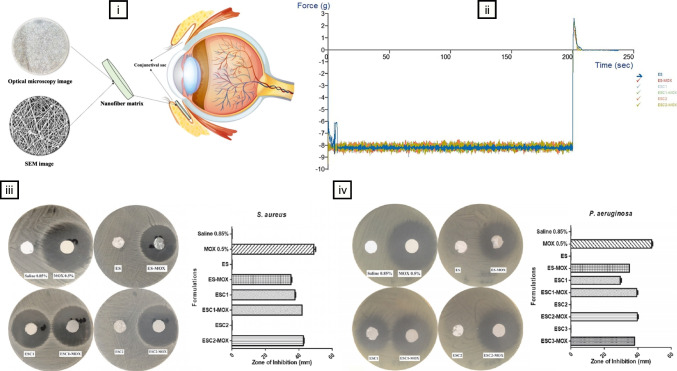
Mucoadhesive electrospun nanofibrous PCL/PLA matrices for the ocular delivery of moxifloxacin. (i) Schematic of the developed nanofiber matrix and its application to the eye. (ii) Results of ex vivo bioadhesion studies. (iii) and (iv) show inhibition zones caused by blank and MOX-loaded formulations in *S. aureus* and *P. aeruginosa*, respectively. *Reproduced with permission from* [314]*, Copyright Taylor & Francis, 2024*

Moxifloxacin (MOX) is a fourth-generation fluoroquinolone antibiotic known for its broad-spectrum antimicrobial activity. In ocular applications, it is commonly used to treat infections such as bacterial conjunctivitis, keratitis, and postoperative prophylaxis due to its excellent penetration and rapid action. Çağlar et al. [314] developed electrospun PCL/PLA nanofibers coated with a novel hyaluronic acid (HA) and xanthan gum (XA) blend for ocular moxifloxacin (MOX) delivery, targeting bacterial infections. This work harnesses mucoadhesion to counter rapid precorneal clearance, transforming hydrophobic nanofibers into bioadhesive ocular inserts. The HA/XA coating, leveraging HA’s hydrogen bonding and XA’s ionic interactions with mucin, enhanced retention, with ex vivo goat cornea tests showing the 0.2% HA/XA variant (ESC2-MOX) achieving a work of adhesion (0.0475 ± 0.0128 N·s) sufficient to resist blinking forces. Unlike uncoated fibers, where static charge drove adhesion, the coating’s mucoadhesive capacity tuned release: ESC1-MOX (0.1%) delivered ~ 80% MOX in 10 days (60% in 24 h) for acute needs, while ESC2-MOX slowed to ~ 30%, ideal for prophylaxis. Antimicrobial efficacy was demonstrated by the large inhibition zones, particularly for *Staphylococcus aureus* (~ 42 mm) and *Pseudomonas aeruginosa* (~ 39.67 mm), confirming potent activity against common ocular pathogens (Fig. 7). Remarkably, the coated nanofibers also supported L929 fibroblast viability (up to 115.49 ± 2.14% for ESC2-MOX), hinting at tissue-healing potential beyond drug delivery. However, the study’s reliance on ex vivo rather than in vivo retention data limits its translational certainty, and the interplay between coating-induced film formation and release kinetics warrants deeper mechanistic exploration. Compared to single-polymer coatings (e.g., alginate or chitosan), the HA/XA blend increases mucoadhesion by integrating HA’s hydrogen bonding with XA’s ionic effects, though the sodium salt form of XA may weaken these interactions, a detail future work could optimize.

In comparison, Youssef et al. [315] developed a moxifloxacin-loaded nanoemulsion (NE) with mucoadhesive agents (MOX-NEM), revealing distinct differences. The MOX-NE and MOX-NEM formulations exhibited sustained release over 12 h and a 2.1-fold improvement in transcorneal permeation compared to Vigamox® eyedrops. However, the mucoadhesive nanofibers provide a far more prolonged release, with only 30% of the drug released over 10 days, outlasting the nanoemulsion’s 12-h profile. This extended release is vital for conditions requiring sustained antibiotic exposure. Another study by Gade et al. [316] developed a drug-eluting polymeric contact lens for delivering moxifloxacin and dexamethasone, achieving sustained release up to 24 h and improved corneal drug distribution compared to standard solutions. Yet, the nanofibers offer a non-invasive alternative, avoiding the contact lens’s potential drawbacks, such as discomfort, blurred vision, or altered corneal oxygen permeability from prolonged wear. The nanofibers adhere to the ocular surface without requiring direct corneal placement. Future work could focus on optimizing HA/XA concentrations or combining the nanofibers with additional therapies to address diverse treatment needs, such as rapid high-level antibiotic release for acute bacterial loads and sustained delivery for prophylaxis or healing, alongside extended in vivo studies to confirm safety, mucoadhesion, and therapeutic effectiveness on the ocular surface.

Mehrandish et al. [317] investigated mucoadhesive electrospun nanofibers as an innovative platform for sustained ocular delivery of itraconazole (ITZ), a potent antifungal agent. The study employed polyvinyl alcohol-cellulose acetate (PVA-CA) and polycaprolactone-polyethylene glycol (PCL-PEG) blends to fabricate nanofibers tailored for enhanced corneal retention. Mucoadhesion arises from PVA’s hydroxyl groups, which facilitate hydrogen bonding with corneal mucin, and PEG’s hydrophilic domains, which promote water retention and matrix swelling. These properties enable PVA-CA nanofibers to exhibit greater swelling capacity than PCL-PEG variants, resulting in a matrix-erosion-driven ITZ release spanning 55 days, compared to the diffusion-dominated profile of PCL-PEG. This extended release, underpinned by mucoadhesive interactions, addresses the challenge of rapid precorneal clearance inherent to conventional eye drops, offering a potential reduction in dosing frequency for fungal keratitis management. The mucoadhesive nature of PVA-CA also enhances antifungal efficacy, with superior drug diffusion against Candida albicans and Aspergillus fumigatus relative to PCL-PEG formulations. Furthermore, PVA and PEG improve the mechanical flexibility and tensile strength of CA and PCL, respectively, ensuring durability under ocular shear forces, as validated by irritation-free outcomes in a 7-day Draize test.

In comparison, a layer-by-layer biopolymer-coated deformable liposome–in situ gel system developed by Badran et al. [318] reveals the nanofibers’ superior sustained release characteristics. The deformable liposomes (DLs), coated with chitosan (CS) and hyaluronic acid (HA) to improve ocular retention, exhibited significantly faster drug release than nanofibers. While DLs offered enhanced corneal epithelium permeability and higher bioavailability, they relied on additional in situ gel formulations to prolong retention. The liposomal system excelled in transcorneal permeation and rapid antifungal effects, making it advantageous for acute infections. However, the nanofiber system outperforms liposomes in sustained release and prolonged bioavailability, minimizing the need for frequent application. A similar contrast emerges with thermosensitive and mucoadhesive in situ ocular gels for ITZ nanocrystals (NCs) reported by Permana et al. [319]. The NC-based thermosensitive in situ gel improved aqueous solubility and corneal penetration, achieving a 93% reduction in Candida albicans population within 48 h in an ex vivo model. This rapid action renders NC-based gels highly effective for acute fungal infections, yet their shorter retention times necessitate frequent dosing. Conversely, the mucoadhesive nanofibers deliver a gradual and sustained release, reducing the need for multiple daily applications. This difference is pivotal for tailoring ocular drug delivery platforms to distinct clinical needs. While both deformable liposomal systems and nanocrystal-loaded in situ gels enhance ITZ bioavailability, their faster release kinetics make them less suited for long-term treatment of chronic infections. By contrast, the mucoadhesive nanofiber system uniquely integrates high drug-loading capacity, sustained release, and robust bioadhesion, overcoming a critical limitation in ocular drug retention.

### Glaucoma therapy

**Fig. 8:**
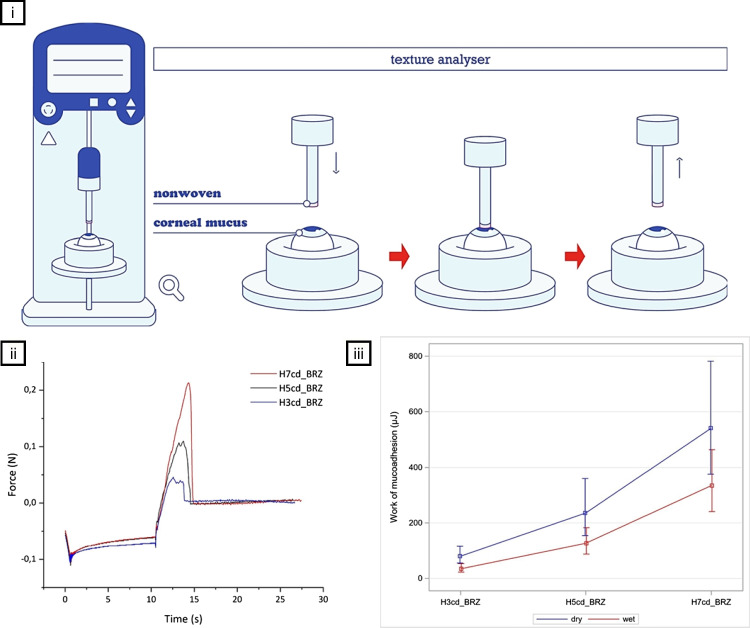
Mucoadhesive brinzolamide-loaded nanofibers for alternative glaucoma treatment. (i) Schematic representation of the experimental setup used for the mucoadhesion study on the corneal surface. (ii) Representative time-force curves obtained during the mucoadhesion test under dry conditions, illustrating the adhesion behavior of BRZ-loaded nanofiber. (iii) Representative curve showing the calculated average work of mucoadhesion for the BRZ-loaded nanofiber. *Reproduced with permission from *[320]*, Copyright Elsevier, 2022*

Glaucoma, a leading cause of irreversible blindness, is characterized by elevated intraocular pressure and progressive optic nerve damage. A range of advanced drug delivery systems are being developed to overcome limitations of conventional eye drops, reduce dosing frequency and the number of different drops, ultimately improving patient adherence. In a recent study, Cegielska et al. [320] developed mucoadhesive brinzolamide (BRZ)-loaded nanofibers for sustained ocular delivery, offering an alternative to the conventional eye drop formulation typically used for glaucoma treatment. The nanofibers were fabricated using electrospinning with β-cyclodextrin (β-CD), hydroxypropyl cellulose (HPC), and PCL blends, achieving smooth morphology with fiber diameters ranging from 300 nm to 1 µm. BRZ was encapsulated with an exceptionally high efficiency in certain formulations, attributed to drug-polymer interactions and β-CD's drug solubilization properties. In vitro drug release studies showed sustained and controlled BRZ release for over 72 h, with no burst release, and exhibited a biphasic profile. Among the tested formulations, H3cd_BRZ, which had a PCL-rich composition, provided optimal drug delivery kinetics. Ex vivo corneal permeation studies using sheep corneas demonstrated enhanced delivery, with H3cd_BRZ achieving the highest cumulative permeation of 61.76 µg/cm^2^ and a steady-state flux of 0.1914 µg/cm^2^/min, significantly outperforming the commercial eye drop formulation Optilamid® (cumulative permeation 44.5 µg/cm^2^ and flux 0.143 µg/cm^2^/min). The strong mucoadhesion of HPC-rich formulations (H7cd_BRZ) improved the nanofibers’ residence time on the corneal surface, addressing challenges associated with rapid clearance (Fig. 8). Although Cegielska et al.’s study presents promising in vitro and ex vivo results, it lacks in vivo data to support these findings. Additional mechanistic insights into mucoadhesion dynamics and long-term stability data would further strengthen its potential for clinical translation.

In comparison, Huang et al. [321] solid drug nanoparticles (SDNs) to improve ocular bioavailability of hydrophobic antiglaucoma drugs, highlighting distinct differences. The SDNs, engineered with brimonidine (BM) and betaxolol (BX) via flash nanoprecipitation, produced uniform sizes (≈150 nm BM, ≈80 nm BX), threefold enhanced corneal permeation (> 30% vs. < 10% for BT/BH in 4 h ex vivo), and sustained IOP reduction (2.91 mmHg vs. 0.88 mmHg in normotensive rats, lasting 120 h). Trehalose-stabilized SDNs exhibited batch consistency, 7-day colloidal stability at 4 °C, and prolonged release (< 40% BM, < 55% BX in 4 h vs. > 60% BT, > 70% BH), improving bioavailability and reducing dosing frequency. However, their dependence on hydrophobic drugs raises risks of systemic absorption, potentially causing side effects (e.g., cardiovascular effects from beta-blockers), and their release profile, though sustained, may be less ideal for acute glaucoma needing rapid IOP reduction. By contrast, the mucoadhesive nanofibers, loaded with brinzolamide, utilize HPC-rich compositions for superior corneal adhesion (ex vivo sheep cornea testing) and a burst-free release over 72 h, ensuring localized delivery. Similarly, Lin et al. [322] developed an in situ-crosslinked hydrogel for inducing chronic ocular hypertension (COH) in a glaucoma model, using an injectable hydrogel of hyperbranched poly(ethylene glycol) (HB-PEG) and thiolated hyaluronic acid (HA-SH) to block aqueous humor outflow, resulting in sustained IOP elevation and retinal ganglion cell (RGC) loss. While effective, this invasive method involves risks like injection-related discomfort, potential tissue damage, and the need for clinical oversight. The mucoadhesive nanofibers, applied topically to the ocular surface, offer a non-invasive alternative. Despite challenges such as the lack of a rapid-onset mechanism for acute IOP spikes, limited data on mechanical robustness under blinking or tear fluid dynamics, and scalability issues with electrospinning for commercial production, the HPC-based nanofiber platform uniquely integrates strong mucoadhesion with controlled, extended release. This makes it particularly well-suited for chronic glaucoma management, where patient adherence and minimized ocular irritation are critical.

### Corneal wound healing

Insulin’s role in ocular surface homeostasis, mediated by receptors on the cornea and lacrimal gland, underpins its potential to promote wound healing and mitigate dry eye syndrome and corneal lesions, particularly in diabetic patients. This has driven the development of targeted delivery systems to optimize its therapeutic impact. Voronova et al.’s [323] photothermally activated mucoadhesive nanofiber mats, constructed from poly(acrylic acid) (PAA) and reduced graphene oxide (rGO) crosslinked with β-cyclodextrin, exemplify this approach. These nanofibers, with an average diameter of 400 ± 150 nm, adhere robustly to the corneal mucosa, leveraging mucoadhesion to extend insulin residence time. Photothermal activation via a 980 nm near-infrared laser (500 mW/cm^2^) elevates the fiber surface temperature to 51 ± 2 °C, triggering a controlled release that achieves an insulin flux of 24.3 ± 3.1 μg/cm^2^/h across porcine corneas ex vivo—outpacing buccal mucosa (e.g., ~ 10 μg/cm^2^/h) and skin-based systems. Over 6 h, 37 ± 1% of the loaded insulin permeates the cornea, with 25% remaining adhered to the corneal tissue, highlighting the mats’ capacity for sustained, localized delivery. However, the need for laser activation introduces complexity and raises safety concerns about repeated exposure, suggesting exploration of alternatives like pH- or temperature-responsive polymers to simplify clinical use.

Comparative analysis with other ophthalmic insulin strategies reveals the mucoadhesive nanofibers’ advantages. Chen et al. [324] employed insulin eye drops at 1.5 IU/mL, administered four times daily to STZ-induced diabetic mice, achieving significant corneal epithelial closure within 72 h (assessed via fluorescein staining) and a 30% increase in nerve density (via substance P and CGRP immunofluorescence). These results confirm insulin’s regenerative potential, but the frequent dosing, driven by rapid tear turnover (clearance half-life ~ 2–5 min), reduces practicality and risks inconsistent drug levels. In contrast, the nanofibers leverage mucoadhesion to anchor insulin, potentially reducing dosing to once daily or less, while their photothermal control could maintain therapeutic concentrations (e.g., > 1 μg/cm^2^/h flux) beyond the transient peaks of drops. This extended contact may enhance insulin’s interaction with corneal receptors, amplifying nerve regeneration and epithelial repair beyond the eye drop outcomes. Likewise, Cruz-Cazarim et al. [325] improved retention using chitosan microparticles and chitosan/poloxamer thermos-reversible gels, delivering insulin at 1 IU/mL once daily to diabetic Wistar rats. After 5 days, tear secretion doubled from ~ 5 mm to > 10 mm on Schirmer’s test (p < 0.05) compared to untreated controls, and by day 15, corneal epithelial thickness increased by ~ 20 μm, indicating enhanced regeneration. The mucoadhesive chitosan and poloxamer 407’s gelation at ocular temperature extends residence time, but the passive release from these systems lacks precise kinetic control, resulting in a steady yet unoptimized insulin profile with potential variability in bioavailability. The nanofibers, however, combine mucoadhesion with tunable release, achieving a higher flux (24.3 vs. estimated < 15 μg/cm^2^/h for gels) and retaining insulin at the cornea longer (25% vs. likely < 10% for GELMP), offering a more efficient delivery mechanism. The nanofibers’ advantage lies in maximizing insulin-tissue intimacy. Their adhesion, driven by PAA’s carboxyl groups and β-cyclodextrin’s hydrogen bonding, creates a stable drug reservoir, contrasting with the brief exposure of the eye drops (cleared within minutes) and the less adhesive microparticles in the gels (dispersed but not inherently fibrous).

**Fig. 9:**
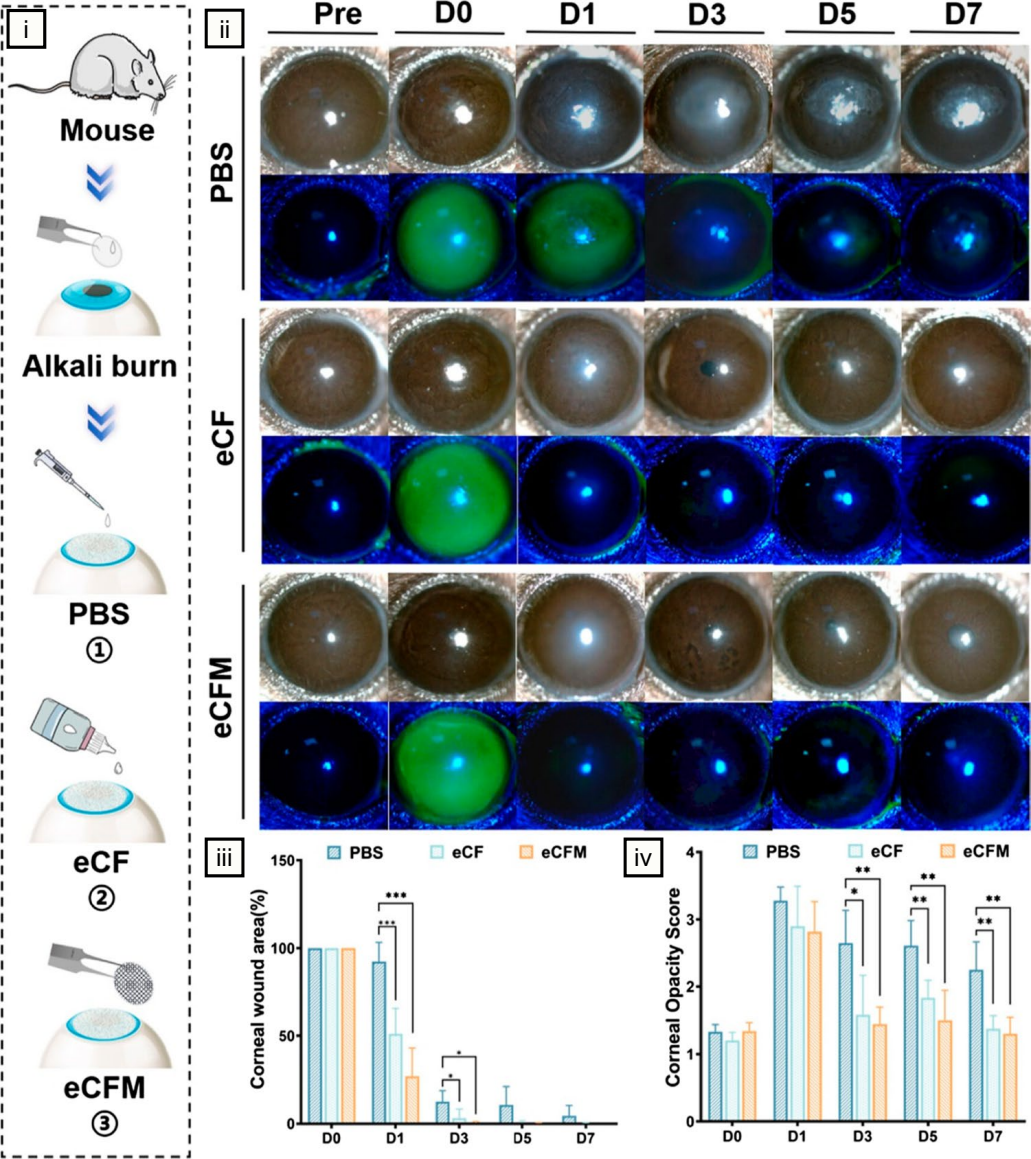
In vivo evaluation of electrospun collagen nanofibers (eCF) for enhanced wound healing in alkali-burned corneas. (i) Schematic representation of the in vivo experimental model used to study corneal wound healing. (ii) Slit-lamp observations and fluorescein staining of alkali-burned corneas treated with PBS, eCF, and eCFM at 0, 1-, 3-, 5-, and 7-days post-operation. (iii) Quantitative analysis of the corneal epithelial wound area over time. (iv) Corneal opacity scores at each time point. *Reproduced with permission from *[326]*, Copyright American Chemical Society, 2024*

Corneal trauma presents significant challenges to effective healing, as the natural repair process often results in fibrosis and scar formation, which can severely impair vision. Recently, Zhang et al. [326] developed electrospun collagen nanofibers (eCFs) with enhanced mucoadhesive properties, aiming to reduce inflammation, inhibit fibrosis, and promote wound healing on the ocular surface. The nanofibers, fabricated from type I collagen, exhibited diameters of 301 ± 58 nm with uniform, interconnected porosity, mimicking the natural extracellular matrix (ECM). The mechanical properties of eCFs, with a tensile strength of 3.2 ± 0.3 MPa and elongation at break of 29.4 ± 4.1%, support their ability to adhere to the ocular mucosa while maintaining flexibility, a crucial factor in minimizing mechanical irritation during blinking. In vitro, eCFs significantly mitigated inflammation in lipopolysaccharide (LPS)-stimulated human corneal epithelial cells (hCECs) and stromal fibroblasts (hCSFs). The nanofibers reduced IL-6, IL-8, and TNF-α mRNA levels by 64%, 58%, and 71%, respectively, while protein levels decreased by up to 75% (p < 0.001). The mucoadhesive nature of eCFs plays a vital role in their ability to modulate corneal wound healing. The nanofibers enhanced cell viability under inflammatory conditions, increasing hCEC and hCSF survival by 29% and 34%, respectively, respectively, compared to untreated controls. Additionally, eCFs prevented excessive fibrosis by suppressing α-SMA expression by 63% while upregulating ALDH3A1 by 72%, thereby maintaining keratocyte quiescence and inhibiting myofibroblast differentiation. These effects, facilitated by the prolonged retention of eCFs on the mucosal surface, contribute to their ability to promote regenerative healing while preventing corneal scarring. In vivo, eCFs accelerated epithelial closure in an alkali-burned mouse model, achieving 92% defect closure by day 3, compared to 65% in controls, reinforcing their role as an effective mucoadhesive scaffold for corneal repair. Corneal opacity was reduced by 45%, with significant restoration of stromal transparency (Fig. 9). Histological analysis confirmed reduced neutrophil infiltration, suppressed fibrotic remodeling, and enhanced epithelial and stromal regeneration after 7 days of treatment. These findings highlight their potential as a promising scaffold for corneal injury repair.

In comparison, a gelatin-based photocurable hydrogel system for corneal wound repair developed by Li et al. [327] reveals distinct therapeutic differences. The thiol-acrylate crosslinked gelatin hydrogels offered an injectable, photocurable platform with tunable mechanical properties, addressing a limitation of eCFs’ fixed mechanical characteristics post-fabrication. While eCFs provide sufficient robustness and flexibility for topical ocular use, their lack of in situ adaptability contrasts with the hydrogels’ ability to adjust stiffness and elasticity for customized defect repair. These hydrogels demonstrated excellent biocompatibility, supported corneal epithelial regeneration, and achieved rapid defect closure in rabbit models within three days, matching eCFs’ timeline. However, their reliance on UV irradiation for crosslinking raises concerns about phototoxicity and DNA damage in ocular tissues. Moreover, despite high transparency and adaptability to irregular defects, they lack the nanofibrous architecture of eCFs, which guides cellular migration and promotes organized tissue regeneration. Another comparison with GelCORE, a visible light-crosslinked bioadhesive hydrogel for corneal repair, developed by Shirzaei Sani et al. [328], further highlights eCFs’ strengths. GelCORE exhibited strong bioadhesion, rapid defect sealing, and high transparency, making it a robust option for corneal stromal regeneration. Unlike eCFs, which rely on passive integration with host tissue, GelCORE actively adheres to the defect site, providing immediate stabilization. However, while GelCORE excels in mechanical tunability and adhesion strength, it does not inherently deliver sustained anti-inflammatory effects or mimic the native ECM as effectively as eCFs. This reveals a trade-off between bioadhesion and bioactivity: eCFs create a biologically favorable environment for tissue repair but need mechanical optimization, whereas hydrogels offer structural support but may not fully replicate native corneal ECM interactions. Given these comparisons, the electrospun collagen nanofibers present a compelling case for biomimetic corneal regeneration, particularly in reducing inflammation and fibrosis. Future work should focus on enhancing their mechanical stability and long-term integration, potentially by hybridizing eCFs with adhesive hydrogels to merge bioactivity with mechanical resilience, enabling both sustained regenerative benefits and immediate wound stabilization.

## Translational challenges and recommendations

Ocular drug delivery systems leveraging mucoadhesion face distinct technical challenges due to their prolonged and modified interaction with the ocular mucosa. In the case of electrospun nanofibers, biocompatibility is an important consideration as the materials used must not only be effective in adhering to mucosal surfaces but also remain safe for long-term exposure without causing adverse reactions. Studies on mucoadhesive systems suggest that adhesion times can vary widely, ranging from several hours to days, depending on factors like material composition, formulation design, and the ocular environment, particularly in the context of disease conditions, though specific durations are often tied to the experimental models and materials being investigated [329]. While blending synthetic polymers is a favored approach for enhancing adhesion, the chemical makeup and breakdown products of these polymers can be cytotoxic, particularly at high concentrations or with extended use [330, 331]. In contrast, natural polymers such as chitosan, hyaluronic acid, and alginate have demonstrated superior biocompatibility and lower cytotoxicity due to their structural similarity to biological macromolecules and their enzymatic degradation pathways, which produce non-toxic byproducts [332]. Studies have shown that natural polymers generally exhibit better cell viability, reduced inflammatory responses, and enhanced tissue integration compared to some synthetic alternatives, making them attractive for biomedical applications [333, 334]. Continuous exposure to synthetic polymers can irritate or damage cellular structures, compromising mucosal barrier integrity. Surface treatments using harsh chemical processes to modify nanofiber surfaces may worsen these effects by introducing reactive or unstable groups that increase cytotoxicity risks, particularly in sensitive nasal or ocular mucosa [335]. Moreover, the degradation byproducts of non-biodegradable or slow-degrading polymers can persist in tissues, potentially triggering toxicity or inflammation if they interfere with cellular function or the local microenvironment. Polymers degrading into acidic components may also alter mucosal pH and homeostasis, heightening irritation or infection risks [336].

Ensuring long-term biocompatibility is a major challenge for mucoadhesive nanofibers, given the delicate nature and rapid renewal of mucosal tissues. These materials must be non-irritating, avoid disrupting normal tissue healing or turnover, and produce degradation products that remain harmless throughout their lifecycle, a critical requirement for treating chronic conditions at these sites [337]. The continuous turnover of mucosal surfaces, combined with the potential for inflammatory responses or delayed adverse effects, complicates the assurance of long-term safety. Standard in vitro methods, such as monolayer cell cultures (e.g., human corneal epithelial cell lines) or simple co-culture systems, are widely used to assess biocompatibility and cytotoxicity [338, 339]. However, these models fail to replicate the dynamic physiological environment of the ocular mucosa. They lack the complex multilayered structure of the corneal epithelium, a functional tear film, and the constant blinking mechanics that influence mucosal turnover and material interactions in vivo. Additionally, these static systems inadequately address immune responses or the gradual degradation of nanofibers over time, which may result in unforeseen inflammatory reactions or delayed adverse effects, complicating predictions of long-term tissue compatibility. To address these shortcomings, advanced systems such as organ-on-chip platforms [340–342] provide more accurate representations of the eye’s dynamic conditions, incorporating its multilayered structure and mechanical interactions. While accelerated degradation studies conducted in the lab can offer insights into polymer breakdown, they frequently misrepresent real-time degradation and byproduct interactions in vivo. Extensive in vitro testing remains critical, utilizing controlled conditions to assess biocompatibility, cytotoxicity, and degradation behavior. Since short-term cytotoxicity assays may not detect the effects of chronic exposure, advanced techniques such as long-term co-culture systems, repeated dosing studies, and real-time degradation monitoring are increasingly utilized to minimize adverse biological effects prior to preclinical evaluation [343, 344]. Preclinical testing in animal models, adapted to include prolonged observation periods, repeated dosing regimens, and environments that closely mimic human mucosal tissues, further evaluates safety and efficacy over extended durations. These modifications allow for detailed assessment of inflammatory responses, tissue regeneration capabilities, and potential adverse reactions under physiological conditions, providing a comprehensive understanding of nanofiber behavior in complex biological systems [345]. Collectively, these rigorous in vitro and preclinical testing stages are essential for validating the safety and effectiveness of mucoadhesive nanofibers, ensuring their suitability for advancement to clinical trials [346].

Scaling up nanofiber-based drug delivery systems to industrial and clinical levels also presents significant challenges, due to the complex nature of their fabrication. These challenges are aggravated when incorporating mucoadhesive properties into the nanofibers [347]. The process of electrospinning, used in the fabrication of these nanofibers, is highly susceptible to slight variations in operational conditions and environmental factors. These minor changes can result in significant inconsistencies in the nanofibers'diameter, porosity, and surface structure [348]. The effectiveness of mucoadhesion in these nanofibers largely depends on the quality of the polymer used. Variations in the polymer's molecular weight distribution, purity, or processing conditions can lead to irregularities in the strength and longevity of adhesion. When mucoadhesive properties are enhanced by blending different polymers, maintaining a consistent ratio and even distribution becomes more challenging at a larger scale, affecting the overall mucoadhesive performance [349, 350]. Moreover, achieving uniform surface chemistry across extensive batches poses difficulties, especially when surface treatments/functionalization are applied. To ensure reliable mucoadhesive performance, the product specification for these nanofibers should clearly define critical parameters such as polymer composition (e.g., specific molecular weight range and purity levels), blend ratios (e.g., a fixed percentage of each polymer type), and target mucoadhesive strength (e.g., measured in terms of adhesion force in Newtons or duration of adhesion under physiological conditions). Additionally, the specification should include standards for surface chemistry consistency, such as the degree of functionalization (e.g., percentage of active sites) and uniformity of coating thickness (e.g., within a tolerance of ± 5 nm). These metrics provide a benchmark for quality and performance, ensuring the nanofibers meet intended therapeutic or functional requirements. Implementing real-time monitoring and developing quality control protocols for raw materials during the electrospinning process, as implemented in non-medical fields using nanofibers, could help maintain output quality. The adoption of closed-loop systems that allow real-time adjustments to critical parameters could further ensure that the final product remains consistent between batches [351, 352].

The final hurdle to the commercialization of mucoadhesive nanofiber drug delivery systems lie in overcoming regulatory challenges. These innovative technologies involve new materials, fabrication techniques, and drug delivery methods, which must meet stringent safety, efficacy, and quality requirements set by regulatory bodies like the FDA (Food and Drug Administration), EMA (European Medicines Agency), and other national agencies [353, 354]. As highlighted in the pre-clinical studies from the previous section, many mucoadhesive nanofiber systems incorporate novel polymers, additives, or modified biomaterials, some of which have not yet been approved for drug delivery. This raises concerns about their long-term safety. Regulatory agencies usually require these new materials to undergo evaluation under ISO 10993 standards for biological safety, which include tests for cytotoxicity, sensitization, and genotoxicity, among others [355, 356]. Beyond these safety requirements, the cost of regulatory approval is a major limiting factor. Extensive toxicology studies, stability testing, and large-scale manufacturing validation require substantial financial investment, often making it impractical to introduce entirely new polymeric materials into the market [357]. As a result, most commercially viable technologies rely on a small number of well-established, regulatory-approved polymers (such as PLGA, PCL, and chitosan) where safety profiles and biocompatibility data are already well-documented. While this accelerates regulatory approval, it also restricts innovation by discouraging the exploration of novel, potentially more effective biomaterials [358].

Nanofibers are a relatively new drug delivery platform, and the limited clinical experience makes it difficult for regulatory bodies to thoroughly assess their safety and efficacy. A key challenge is determining the regulatory classification of these systems; whether they will be regulated as a drug, biologic, or medical device. This classification impacts the regulatory approval process, timelines, and requirements [359]. While not strictly predefined, the regulatory classification of mucoadhesive nanofiber-based systems depends on their primary mode of action. If the system functions mainly as a drug carrier, where the therapeutic effect comes from the active pharmaceutical ingredient (API), it is regulated as a drug product. In some cases, it may be classified as a combination product, requiring both the drug and the nanofiber carrier to meet regulatory standards. If the nanofiber itself provides therapeutic benefits, such as promoting wound healing or acting as a physical barrier, it may be classified as a medical device. [360]. Different classifications require distinct approval pathways and testing protocols. For example, combination products need to undergo both drug and device evaluations, which complicates the approval process by requiring compliance with standards for both categories. Additionally, regulatory agencies typically demand comprehensive clinical data to demonstrate the safety and effectiveness of new drug delivery technologies [361]. The novel nature of nanofibers complicates this process, as there are often no established clinical pathways for evaluating them. This when combined with the high cost of extensive safety testing, stability studies, and large-scale manufacturing validation can be prohibitive, particularly for small companies and academic innovations. To facilitate the clinical transition of next-generation nanofiber-based drug delivery systems, collaboration between regulatory bodies, researchers, and industry stakeholders is essential. Clearer regulatory pathways can be established by reaching consensus on testing standards, clinical endpoints, and acceptable risk levels. This will be key in advancing these systems from the laboratory to the market [362].

## Concluding remarks

Mucoadhesive nanofibers represent a transformative innovation in ocular drug delivery, addressing many limitations of traditional systems. By leveraging the high surface area and tunable properties of nanofibers alongside the adhesive capabilities of mucoadhesive polymers, these systems achieve enhanced retention on the ocular surface, improved drug bioavailability, and controlled release profiles. This review has showcased how mucoadhesive nanofibers effectively surmount the structural and functional challenges of the ocular surface, outperforming conventional drug delivery alternatives in overcoming these barriers.

The electrospinning process enables the production of fibers with tunable diameters, morphologies, and mechanical properties, tailored to meet the demands of ocular drug delivery. By adjusting key parameters such as polymer concentration, applied voltage, and environmental conditions, researchers can design nanofibers optimized for drug encapsulation and sustained release. Additionally, advancements in polymer blending, surface modification, and molecular imprinting have enhanced the mucoadhesive properties of nanofibers, facilitating stronger adhesion to the ocular surface. Polymer blending allows the incorporation of bioadhesive components, enhancing covalent and non-covalent interactions with mucins. Surface modifications, such as plasma treatment or grafting, improve wettability and charge distribution, promoting electrostatic and hydrogen bonding with the tear film. Molecular imprinting enables the creation of mucin-specific binding sites, optimizing drug retention and controlled release. Together, these strategies prolong nanofiber residence time on the ocular surface, allowing for sustained drug diffusion through the cornea and conjunctiva, leading to enhanced drug absorption and bioavailability. These innovations position mucoadhesive nanofibers as a highly effective solution for achieving localized drug delivery in the eye, reducing dosing frequency, and minimizing systemic side effects.

Despite their immense potential, challenges remain in translating mucoadhesive nanofibers into clinical applications for ocular therapies. Ensuring biocompatibility with the delicate tissues of the ocular mucosa is paramount, especially with synthetic polymers, where factors like the nanofibers’ increased surface area or the eye’s heightened sensitivity could potentially amplify irritation or adverse reactions, even with well-studied, generally well-tolerated materials. While electrospinning at a commercial scale has been successfully implemented by several companies, challenges can persist in maintaining batch-to-batch consistency, optimizing fiber reproducibility, and ensuring cost-effective scalability for pharmaceutical applications. Additionally, regulatory frameworks must continue evolving to accommodate the unique nature of these systems, particularly in defining standardized testing protocols for mucoadhesion, ocular retention, and long-term biocompatibility. Existing guidelines for ophthalmic drug delivery primarily focus on conventional formulations, and there is a lack of specific criteria for nanofiber-based systems, including stability, degradation kinetics, and patient safety over prolonged use. Establishing clear regulatory pathways tailored to these advanced biomaterials will be essential for their clinical translation and approval.

Nonetheless, the future of mucoadhesive nanofibers in ocular drug delivery is highly promising. Their ability to provide targeted, sustained, and efficient drug release opens the door to innovative treatments for a wide range of ocular diseases, including dry eye syndrome, conjunctivitis, keratitis, and ocular surface cancers. Additionally, the adaptability of these systems to incorporate both hydrophilic and lipophilic drugs ensures their applicability across diverse therapeutic landscapes. The potential for personalized ocular therapies, where nanofibers are tailored to the specific needs of individual patients, represents a paradigm shift in patient care, offering solutions that are both effective and patient-friendly.

In conclusion, mucoadhesive nanofibers are poised to redefine the field of ocular drug delivery, providing groundbreaking advancements in both therapeutic efficacy and patient experience. Ongoing research tackling current hurdles will likely cement their role in advancing ophthalmic medicine and translating innovative biomaterials into practical treatments.

## Data Availability

Not applicable.

## References

[1] Ezike TC, Okpala US, Onoja UL, Nwike CP, Ezeako EC, Okpara OJ, Okoroafor CC, Eze SC, Kalu OL, Odoh EC, Nwadike UG, Ogbodo JO, Umeh BU, Ossai EC, Nwanguma BC. Advances in drug delivery systems, challenges and future directions. Heliyon. 2023;9:e17488. 10.1016/j.heliyon.2023.e17488. 37416680 10.1016/j.heliyon.2023.e17488 PMC10320272

[2] Polaka S, Desai N, Kshirsagar B, Rajpoot K, Tekade RK. Revamping the pharmacokinetics of poorly soluble drugs using different formulations. In: Biopharmaceutics and Pharmacokinetics Considerations, Elsevier. 2021:387–413. 10.1016/B978-0-12-814425-1.00005-X.

[3] Trucillo P. Biomaterials for drug delivery and human applications. Materials. 2024;17:456. 10.3390/ma17020456. 38255624 10.3390/ma17020456 PMC10817481

[4] Gipson IK. The ocular surface: The challenge to enable and protect vision. Invest Opthalmol Vis Sci. 2007;48:4391. 10.1167/iovs.07-0770. 10.1167/iovs.07-0770 PMC2886589 17898256

[5] Chandel A, Kandav G. Insights into ocular therapeutics: A comprehensive review of anatomy, barriers, diseases and nanoscale formulations for targeted drug delivery. J Drug Deliv Sci Technol. 2024;97:105785. 10.1016/j.jddst.2024.105785.

[6] Giri BR, Jakka D, Sandoval MA, Kulkarni VR, Bao Q. Advancements in ocular therapy: A review of emerging drug delivery approaches and pharmaceutical technologies. Pharmaceutics. 2024;16:1325. 10.3390/pharmaceutics16101325. 39458654 10.3390/pharmaceutics16101325 PMC11511072

[7] Salave S, Patel P, Desai N, Rana D, Benival D, Khunt D, Thanawuth K, Prajapati BG, Sriamornsak P. Recent advances in dosage form design for the elderly: a review. Expert Opin Drug Deliv. 2023;20:1553–71. 10.1080/17425247.2023.2286368. 37978899 10.1080/17425247.2023.2286368

[8] Boddu SHS, Acharya D, Hala V, Jani H, Pande S, Patel C, Shahwan M, Jwala R, Ranch KM. An update on strategies to deliver protein and peptide drugs to the eye. ACS Omega. 2023;8:35470–98. 10.1021/acsomega.3c02897. 37810716 10.1021/acsomega.3c02897 PMC10552503

[9] Ji D, Lin Y, Guo X, Ramasubramanian B, Wang R, Radacsi N, Jose R, Qin X, Ramakrishna S. Electrospinning of nanofibres. Nat Rev Methods Primers. 2024;4:1. 10.1038/s43586-023-00278-z.

[10] Hiwrale A, Bharati S, Pingale P, Rajput A. Nanofibers: A current era in drug delivery system. Heliyon. 2023;9:e18917. 10.1016/j.heliyon.2023.e18917. 37674834 10.1016/j.heliyon.2023.e18917 PMC10477438

[11] Mishra D, Gade S, Pathak V, Vora LK, Mcloughlin K, Medina R, Donnelly RF, Raghu Raj Singh T. Ocular application of electrospun materials for drug delivery and cellular therapies. Drug Discov Today. 2023;28:103676. 10.1016/j.drudis.2023.103676. 37343817 10.1016/j.drudis.2023.103676

[12] Abdulhussain R, Adebisi A, Conway BR, Asare-Addo K. Electrospun nanofibers: Exploring process parameters, polymer selection, and recent applications in pharmaceuticals and drug delivery. J Drug Deliv Sci Technol. 2023;90:105156. 10.1016/j.jddst.2023.105156.

[13] Sahu D, Rath G, Gupta G. Addressing ocular drug delivery challenges with solid nanofiber variants and supramolecular nanofibrous gel composite. J Drug Deliv Sci Technol. 2024;94:105476. 10.1016/j.jddst.2024.105476.

[14] Mansuri S, Kesharwani P, Jain K, Tekade RK, Jain NK. Mucoadhesion: A promising approach in drug delivery system. React Funct Polym. 2016;100:151–72. 10.1016/j.reactfunctpolym.2016.01.011.

[15] Farhaj S, Conway BR, Ghori MU. Nanofibres in drug delivery applications. Fibers. 2023;11:21. 10.3390/fib11020021.

[16] Forrester JV, Dick AD, McMenamin PG, Roberts F, Pearlman E. Anatomy of the eye and orbit. In: The Eye, 4th ed., Elsevier. 2016:1–102.e2. 10.1016/B978-0-7020-5554-6.00001-0.

[17] Baudouin C, Rolando M, Benitez Del Castillo JM, Messmer EM, Figueiredo FC, Irkec M, Van Setten G, Labetoulle M. Reconsidering the central role of mucins in dry eye and ocular surface diseases. Prog Retin Eye Res. 2019;71:68–87. 10.1016/j.preteyeres.2018.11.007. 30471351 10.1016/j.preteyeres.2018.11.007

[18] Gipson IK. Distribution of mucins at the ocular surface. Exp Eye Res. 2004;78:379–88. 10.1016/S0014-4835(03)00204-5. 15106916 10.1016/s0014-4835(03)00204-5

[19] Hodges RR, Dartt DA. Tear film mucins: Front line defenders of the ocular surface; comparison with airway and gastrointestinal tract mucins. Exp Eye Res. 2013;117:62–78. 10.1016/j.exer.2013.07.027. 23954166 10.1016/j.exer.2013.07.027 PMC4222248

[20] Govindarajan B, Gipson IK. Membrane-tethered mucins have multiple functions on the ocular surface. Exp Eye Res. 2010;90:655–63. 10.1016/j.exer.2010.02.014. 20223235 10.1016/j.exer.2010.02.014 PMC2893012

[21] Ablamowicz AF, Nichols JJ. Ocular surface membrane-associated mucins. Ocul Surf. 2016;14:331–41. 10.1016/j.jtos.2016.03.003. 27154035 10.1016/j.jtos.2016.03.003

[22] Dartt DA. Control of mucin production by ocular surface epithelial cells. Exp Eye Res. 2004;78:173–85. 10.1016/j.exer.2003.10.005. 14729350 10.1016/j.exer.2003.10.005

[23] Paulsen F. Cell and molecular biology of human lacrimal gland and nasolacrimal duct mucins. 2006:229–279. 10.1016/S0074-7696(06)49005-7. 10.1016/S0074-7696(06)49005-7 16697285

[24] Spurr-Michaud S, Argüeso P, Gipson I. Assay of mucins in human tear fluid. Exp Eye Res. 2007;84:939–50. 10.1016/j.exer.2007.01.018. 17399701 10.1016/j.exer.2007.01.018 PMC1950265

[25] Argüeso P. Human ocular mucins: The endowed guardians of sight. Adv Drug Deliv Rev. 2022;180:114074. 10.1016/j.addr.2021.114074. 34875287 10.1016/j.addr.2021.114074 PMC8724396

[26] Nichols BA. Conjunctiva. Microsc Res Tech. 1996;33:296–319. 10.1002/(SICI)1097-0029(19960301)33:4%3c296::AID-JEMT2%3e3.0.CO;2-O. 8652888 10.1002/(SICI)1097-0029(19960301)33:4<296::AID-JEMT2>3.0.CO;2-O

[27] Conrady CD, Joos ZP, Patel BCK. Review: The lacrimal gland and its role in dry eye. J Ophthalmol. 2016;2016:1–11. 10.1155/2016/7542929. 10.1155/2016/7542929 PMC4793137 27042343

[28] Dao DPD, Le PH. Histology, Goblet Cells. 2025. 31985989

[29] Singh S, Basu S. The human lacrimal gland: historical perspectives, current understanding, and recent advances. Curr Eye Res. 2020;45:1188–98. 10.1080/02713683.2020.1774065. 32450044 10.1080/02713683.2020.1774065

[30] Dartt DA, Hodges RR, Zoukhri D. Tears and their secretion. 2005:21–82. 10.1016/S1569-2590(05)10002-0.

[31] Baeyens V, Gurny R. Chemical and physical parameters of tears relevant for the design of ocular drug delivery formulations. Pharm Acta Helv. 1997;72:191–202. 10.1016/S0031-6865(97)00021-6. 9372641 10.1016/s0031-6865(97)00021-6

[32] Masoudi S. Biochemistry of human tear film: A review. Exp Eye Res. 2022;220:109101. 10.1016/j.exer.2022.109101. 35508212 10.1016/j.exer.2022.109101

[33] Cher I. Fluids of the ocular surface: concepts, functions and physics. Clin Exp Ophthalmol. 2012;40:634–43. 10.1111/j.1442-9071.2012.02758.x. 22300341 10.1111/j.1442-9071.2012.02758.x

[34] Paulsen F, Schaudig U, Thale AB. Drainage of tears: Impact on the ocular surface and lacrimal system. Ocul Surf. 2003;1:180–91. 10.1016/S1542-0124(12)70013-7. 17075649 10.1016/s1542-0124(12)70013-7

[35] Morrison PWJ, Khutoryanskiy VV. Anatomy of the Eye and the Role of Ocular Mucosa in Drug Delivery, in: Mucoadhesive Materials and Drug Delivery Systems, Wiley. 2014:39–60. 10.1002/9781118794203.ch02.

[36] Huang R, Su C, Fang L, Lu J, Chen J, Ding Y. Dry eye syndrome: comprehensive etiologies and recent clinical trials. Int Ophthalmol. 2022;42:3253–72. 10.1007/s10792-022-02320-7. 35678897 10.1007/s10792-022-02320-7 PMC9178318

[37] Azari AA, Arabi A. Conjunctivitis: A systematic review. J Ophthalmic Vis Res. 2020;15:372. 10.18502/jovr.v15i3.7456. 32864068 10.18502/jovr.v15i3.7456 PMC7431717

[38] Gilger BC, Degroote R, Deeg C, Diseases of the uvea, uveitis, and recurrent uveitis. In: Equine Ophthalmology, Wiley. 2022:441–498. 10.1002/9781119782285.ch6

[39] Maheshwari A, Finger PT. Cancers of the eye. Cancer Metastasis Rev. 2018;37:677–90. 10.1007/s10555-018-9762-9. 30203109 10.1007/s10555-018-9762-9

[40] Desai N, Sahel D, Kubal B, Postwala H, Shah Y, Chavda VP, Fernandes C, Khatri DK, Vora LK. Role of the extracellular matrix in cancer: insights into tumor progression and therapy. Adv Ther (Weinh). 2025. 10.1002/adtp.202400370.

[41] Balla A, Auriola S, Grey AC, Demarais NJ, Valtari A, Heikkinen EM, Toropainen E, Urtti A, Vellonen K-S, Ruponen M. Partitioning and spatial distribution of drugs in ocular surface tissues. Pharmaceutics. 2021;13:658. 10.3390/pharmaceutics13050658. 34064499 10.3390/pharmaceutics13050658 PMC8147976

[42] Allyn MM, Luo RH, Hellwarth EB, Swindle-Reilly KE. Considerations for Polymers Used in Ocular Drug Delivery. Front Med (Lausanne). 2022;8:787644. 10.3389/fmed.2021.787644. 35155469 10.3389/fmed.2021.787644 PMC8831705

[43] Grassiri B, Zambito Y, Bernkop-Schnürch A. Strategies to prolong the residence time of drug delivery systems on ocular surface. Adv Colloid Interface Sci. 2021;288:102342. 10.1016/j.cis.2020.102342. 33444845 10.1016/j.cis.2020.102342

[44] Bertsch P, Bergfreund J, Windhab EJ, Fischer P. Physiological fluid interfaces: Functional microenvironments, drug delivery targets, and first line of defense. Acta Biomater. 2021;130:32–53. 10.1016/j.actbio.2021.05.051. 34077806 10.1016/j.actbio.2021.05.051

[45] Bartkowiak A, Rojewska M, Hyla K, Zembrzuska J, Prochaska K. Surface and swelling properties of mucoadhesive blends and their ability to release fluconazole in a mucin environment. Colloids Surf B Biointerfaces. 2018;172:586–93. 10.1016/j.colsurfb.2018.09.014. 30218984 10.1016/j.colsurfb.2018.09.014

[46] Jain S, Sankar R. Development and characterization of gastroretentive sustained-release formulation by combination of swelling and mucoadhesive approach: a mechanistic study. Drug Des Devel Ther. 2013;5:1455–69. 10.2147/DDDT.S52890. 10.2147/DDDT.S52890 PMC3857114 24348022

[47] Chatterjee B, Amalina N, Sengupta P, Mandal UK. Mucoadhesive Polymers and Their Mode of Action: A Recent Update. J Appl Pharm Sci. 2017;7:195–203. 10.7324/JAPS.2017.70533.

[48] Burgalassi S, Monti D, Tampucci S, Chetoni P. In vitro evaluation of some parameters involved in mucoadhesion of aqueous polymeric dispersions. Pharm Dev Technol. 2015;20:927–34. 10.3109/10837450.2014.943406. 25059381 10.3109/10837450.2014.943406

[49] Rojewska M, Olejniczak-Rabinek M, Bartkowiak A, Snela A, Prochaska K, Lulek J. The wettability and swelling of selected mucoadhesive polymers in simulated saliva and vaginal fluids. Colloids Surf B Biointerfaces. 2017;156:366–74. 10.1016/j.colsurfb.2017.05.042. 28551570 10.1016/j.colsurfb.2017.05.042

[50] Ratner BD, Castner DG. Surface properties and surface characterization of biomaterials. In: Biomater Sci, 4th ed., Elsevier. 2020:53–75. 10.1016/B978-0-12-816137-1.00006-4.

[51] Agrawal G, Negi YS, Pradhan S, Dash M, Samal SK. Wettability and contact angle of polymeric biomaterials. In: Characterization of Polymeric Biomaterials, Elsevier. 2017:57–81. 10.1016/B978-0-08-100737-2.00003-0.

[52] Shaikh R, Raj Singh TR, Garland MJ, Woolfson AD, Donnelly RF. Mucoadhesive drug delivery systems. J Pharm Bioallied Sci. 2011;3:89. 10.4103/0975-7406.76478. 21430958 10.4103/0975-7406.76478 PMC3053525

[53] Laffleur F, Netsomboon K, Bernkop-Schnürch A, Westmeier D, Stauber RH, Docter D. Comprehensive mucoadhesive study of anionic polymers and their derivate. Eur Polym J. 2017;93:314–22. 10.1016/j.eurpolymj.2017.06.012.

[54] Tanaka M. Physics of interactions at biological and biomaterial interfaces. Curr Opin Colloid Interface Sci. 2013;18:432–9. 10.1016/j.cocis.2013.07.002.

[55] McGhee EO, Hart SM, Urueña JM, Sawyer WG. Hydration control of gel-adhesion and muco-adhesion. Langmuir. 2019;35:15769–75. 10.1021/acs.langmuir.9b02816. 31659909 10.1021/acs.langmuir.9b02816

[56] Lee L-H. Adhesion and surface-hydrogen-bond components for polymers and biomaterials. J Adhes. 1998;67:1–18. 10.1080/00218469808011096.

[57] Khutoryanskaya OV, Morrison PWJ, Seilkhanov SK, Mussin MN, Ozhmukhametova EK, Rakhypbekov TK, Khutoryanskiy VV. Hydrogen-bonded complexes and blends of poly(acrylic acid) and methylcellulose: Nanoparticles and mucoadhesive films for ocular delivery of riboflavin. Macromol Biosci. 2014;14:225–34. 10.1002/mabi.201300313. 24106128 10.1002/mabi.201300313

[58] Bovone G, Dudaryeva OY, Marco-Dufort B, Tibbitt MW. Engineering hydrogel adhesion for biomedical applications via chemical design of the junction. ACS Biomater Sci Eng. 2021;7:4048–76. 10.1021/acsbiomaterials.0c01677. 33792286 10.1021/acsbiomaterials.0c01677

[59] Wu L, Shan W, Zhang Z, Huang Y. Engineering nanomaterials to overcome the mucosal barrier by modulating surface properties. Adv Drug Deliv Rev. 2018;124:150–63. 10.1016/j.addr.2017.10.001. 28989056 10.1016/j.addr.2017.10.001

[60] Smart JD. Theories of Mucoadhesion. In: Mucoadhesive Materials and Drug Delivery Systems, Wiley. 2014:159–174. 10.1002/9781118794203.ch07.

[61] Dedinaite A, Lundin M, Macakova L, Auletta T. Mucin−chitosan complexes at the solid−liquid interface: Multilayer formation and stability in surfactant solutions. Langmuir. 2005;21:9502–9. 10.1021/la0511844. 16207028 10.1021/la0511844

[62] Collado-González M, González Espinosa Y, Goycoolea FM. Interaction between chitosan and mucin: Fundamentals and applications. Biomimetics. 2019;4:32. 10.3390/biomimetics4020032. 31105217 10.3390/biomimetics4020032 PMC6631199

[63] Chen J, Pei Z, Chai B, Jiang P, Ma L, Zhu L, Huang X. Engineering the dielectric constants of polymers: From molecular to mesoscopic scales. Adv Mater. 2023. 10.1002/adma.202308670. 38100840 10.1002/adma.202308670

[64] Tofail SAM, Bauer J. Electrically polarized biomaterials. Adv Mater. 2016;28:5470–84. 10.1002/adma.201505403. 27122372 10.1002/adma.201505403

[65] Serra L, Doménech J, Peppas NA. Engineering design and molecular dynamics of mucoadhesive drug delivery systems as targeting agents. Eur J Pharm Biopharm. 2009;71:519–28. 10.1016/j.ejpb.2008.09.022. 18976706 10.1016/j.ejpb.2008.09.022 PMC2680154

[66] Singh I, Pawar P, Sanusi EA, Odeku OA. Mucoadhesive Polymers for Drug Delivery Systems, in: Adhesion in Pharmaceutical, Biomedical and Dental Fields, Wiley. 2017:89–113. 10.1002/9781119323716.ch5.

[67] Pathak K, Malviya R. Introduction, theories and mechanisms of bioadhesion. In: Bioadhesives in Drug Delivery, Wiley. 2020:1–27. 10.1002/9781119640240.ch1.

[68] Baus RA, Zahir-Jouzdani F, Dünnhaupt S, Atyabi F, Bernkop-Schnürch A. Mucoadhesive hydrogels for buccal drug delivery: In vitro-in vivo correlation study. Eur J Pharm Biopharm. 2019;142:498–505. 10.1016/j.ejpb.2019.07.019. 31330258 10.1016/j.ejpb.2019.07.019

[69] Baus RA, Haug MF, Leichner C, Jelkmann M, Bernkop-Schnürch A. In vitro–in vivo correlation of mucoadhesion studies on buccal mucosa. Mol Pharm. 2019;16:2719–27. 10.1021/acs.molpharmaceut.9b00254. 31038970 10.1021/acs.molpharmaceut.9b00254

[70] Dillard DA. Applying fracture mechanics to adhesive bonds. In: Adhesive Bonding, 2nd ed., Elsevier. 2021:295–316. 10.1016/B978-0-12-819954-1.00014-9.

[71] Awaja F. Autohesion of polymers. Polymer (Guildf). 2016;97:387–407. 10.1016/j.polymer.2016.05.043.

[72] Schattling P, Taipaleenmäki E, Zhang Y, Städler B. A polymer chemistry point of view on mucoadhesion and mucopenetration. Macromol Biosci. 2017;17:1700060. 10.1002/mabi.201700060. 10.1002/mabi.201700060 28675773

[73] Taipaleenmäki E, Städler B. Recent advancements in using polymers for intestinal mucoadhesion and mucopenetration. Macromol Biosci. 2020;20:1900342. 10.1002/mabi.201900342. 10.1002/mabi.201900342 32045102

[74] Raos G, Zappone B. Polymer adhesion: Seeking new solutions for an old problem. Macromolecules. 2021;54:10617–44. 10.1021/acs.macromol.1c01182.

[75] Sriamornsak P, Wattanakorn N, Nunthanid J, Puttipipatkhachorn S. Mucoadhesion of pectin as evidence by wettability and chain interpenetration. Carbohydr Polym. 2008;74:458–67. 10.1016/j.carbpol.2008.03.022.

[76] Zhang Q, Li X, Jasti BR. Role of physicochemical properties of some grades of hydroxypropyl methylcellulose on in vitro mucoadhesion. Int J Pharm. 2021;609:121218. 10.1016/j.ijpharm.2021.121218. 34687813 10.1016/j.ijpharm.2021.121218

[77] Hackelbusch S, Rossow T, van Assenbergh P, Seiffert S. Chain dynamics in supramolecular polymer networks. Macromolecules. 2013;46:6273–86. 10.1021/ma4003648.

[78] Dupré de Baubigny J, Perrin P, Pantoustier N, Salez T, Reyssat M, Monteux C. Growth mechanism of polymer membranes obtained by H-bonding across immiscible liquid interfaces. ACS Macro Lett. 2021;10:204–9. 10.1021/acsmacrolett.0c00847. 35570784 10.1021/acsmacrolett.0c00847

[79] Chu JN, Traverso G. Foundations of gastrointestinal-based drug delivery and future developments. Nat Rev Gastroenterol Hepatol. 2022;19:219–38. 10.1038/s41575-021-00539-w. 34785786 10.1038/s41575-021-00539-w PMC12053541

[80] Kulkarni R, Fanse S, Burgess DJ. Mucoadhesive drug delivery systems: a promising non-invasive approach to bioavailability enhancement Part I: biophysical considerations. Expert Opin Drug Deliv. 2023;20:395–412. 10.1080/17425247.2023.2181331. 36803111 10.1080/17425247.2023.2181331

[81] Naito K, Ochiai Y, Tsuboi R, Nimura K, Yashiro K. Study by molecular dynamics and first-principles calculation on the influence of length of molecular chain and entanglement of molecular chains on the strength of amorphous polyethylene. J Fiber Sci Technol. 2020;76:267–74. 10.2115/fiberst.2020-0031.

[82] Nam S, Mooney D. Polymeric tissue adhesives. Chem Rev. 2021;121:11336–84. 10.1021/acs.chemrev.0c00798. 33507740 10.1021/acs.chemrev.0c00798

[83] Patlolla VGR, Holbrook WP, Gizurarson S, Kristmundsdottir P. Evaluation of in vitro mucoadhesiveness and texture profile analysis of doxycycline in situ hydrogels. Pharmazie. 2020;75:7–12. 10.1691/ph.2020.9122. 32033626 10.1691/ph.2020.9122

[84] Macocinschi D, Filip D, Ciubotaru B-I, Dumitriu RP, Varganici C-D, Zaltariov M-F. Blends of sodium deoxycholate-based poly(ester ether)urethane ionomer and hydroxypropylcellulose with mucosal adhesiveness. Int J Biol Macromol. 2020;162:1262–75. 10.1016/j.ijbiomac.2020.06.191. 32585272 10.1016/j.ijbiomac.2020.06.191

[85] Suchaoin W, Pereira de Sousa I, Netsomboon K, Rohrer J, Hoffmann Abad P, Laffleur F, Matuszczak B, Bernkop-Schnürch A. Mucoadhesive polymers: Synthesis and in vitro characterization of thiolated poly(vinyl alcohol). Int J Pharm. 2016;503:141–9. 10.1016/j.ijpharm.2016.03.006. 26965199 10.1016/j.ijpharm.2016.03.006

[86] Worch JC, Prydderch H, Jimaja S, Bexis P, Becker ML, Dove AP. Stereochemical enhancement of polymer properties. Nat Rev Chem. 2019;3:514–35. 10.1038/s41570-019-0117-z.

[87] Kruger AG, Brucks SD, Yan T, Cárcarmo-Oyarce G, Wei Y, Wen DH, Carvalho DR, Hore MJA, Ribbeck K, Schrock RR, Kiessling LL. Stereochemical control yields mucin mimetic polymers. ACS Cent Sci. 2021;7:624–30. 10.1021/acscentsci.0c01569. 34056092 10.1021/acscentsci.0c01569 PMC8155468

[88] Menchicchi B, Fuenzalida JP, Hensel A, Swamy MJ, David L, Rochas C, Goycoolea FM. Biophysical analysis of the molecular interactions between polysaccharides and mucin. Biomacromol. 2015;16:924–35. 10.1021/bm501832y. 10.1021/bm501832y 25630032

[89] De Souza Ferreira SB, Moço TD, Borghi-Pangoni FB, Junqueira MV, Bruschi ML. Rheological, mucoadhesive and textural properties of thermoresponsive polymer blends for biomedical applications. J Mech Behav Biomed Mater. 2016;55:164–78. 10.1016/j.jmbbm.2015.10.026. 10.1016/j.jmbbm.2015.10.026 26590909

[90] Schneider H, Pelaseyed T, Svensson F, Johansson MEV. Study of mucin turnover in the small intestine by in vivo labeling. Sci Rep. 2018;8:5760. 10.1038/s41598-018-24148-x. 29636525 10.1038/s41598-018-24148-x PMC5893601

[91] Melhem H, Regan-Komito D, Niess JH. Mucins dynamics in physiological and pathological conditions. Int J Mol Sci. 2021;22:13642. 10.3390/ijms222413642. 34948435 10.3390/ijms222413642 PMC8707880

[92] Hsein H, Garrait G, Beyssac E, Hoffart V. Whey protein mucoadhesive properties for oral drug delivery: Mucin–whey protein interaction and mucoadhesive bond strength. Colloids Surf B Biointerfaces. 2015;136:799–808. 10.1016/j.colsurfb.2015.10.016. 26529388 10.1016/j.colsurfb.2015.10.016

[93] de Oliveira Cardoso VM, Gremião MPD, Cury BSF. Mucin-polysaccharide interactions: A rheological approach to evaluate the effect of pH on the mucoadhesive properties. Int J Biol Macromol. 2020;149:234–45. 10.1016/j.ijbiomac.2020.01.235. 31982533 10.1016/j.ijbiomac.2020.01.235

[94] Kulkarni D, Giram P, Mahore J, Kapare H, Panzade P. Electrospun nanofibers: a promising paradigm for biomedical applications. Int J Polym Mater Polym Biomater. 2024;74(5):403–23. 10.1080/00914037.2024.2335173.

[95] Wang C, Su Y, Xie J. Advances in electrospun nanofibers: Versatile materials and diverse biomedical applications. Acc Mater Res. 2024. 10.1021/accountsmr.4c00145.39882339

[96] Sujitha AS, Saikant R, Ragupathy L, Hubert Joe I, Painuly D. Gold nanoparticles-incorporated electrospun nanofibrous membrane for optical biosensing applications: An experimental and computational approach. Fibers and Polymers. 2024;25:1193–210. 10.1007/s12221-024-00511-w.

[97] Abdul Hameed MM, Mohamed Khan SAP, Thamer BM, Rajkumar N, El-Hamshary H, El-Newehy M. Electrospun nanofibers for drug delivery applications: Methods and mechanism. Polym Adv Technol. 2023;34:6–23. 10.1002/pat.5884.

[98] Moazzami Goudarzi Z, Behzad T, Ghasemi-Mobarakeh L, Kharaziha M. An investigation into influence of acetylated cellulose nanofibers on properties of PCL/Gelatin electrospun nanofibrous scaffold for soft tissue engineering. Polymer (Guildf). 2021;213:123313. 10.1016/j.polymer.2020.123313.

[99] Raja IS, Preeth DR, Vedhanayagam M, Hyon S-H, Lim D, Kim B, Rajalakshmi S, Han D-W. Polyphenols-loaded electrospun nanofibers in bone tissue engineering and regeneration. Biomater Res. 2021;25:29. 10.1186/s40824-021-00229-3. 34563260 10.1186/s40824-021-00229-3 PMC8466400

[100] Najafi R, Chahsetareh H, Pezeshki-Modaress M, Aleemardani M, Simorgh S, Davachi SM, Alizadeh R, Asghari A, Hassanzadeh S, Bagher Z. Alginate sulfate/ECM composite hydrogel containing electrospun nanofiber with encapsulated human adipose-derived stem cells for cartilage tissue engineering. Int J Biol Macromol. 2023;238:124098. 10.1016/j.ijbiomac.2023.124098. 36948341 10.1016/j.ijbiomac.2023.124098

[101] Yang Y, Du Y, Zhang J, Zhang H, Guo B. Structural and functional design of electrospun nanofibers for hemostasis and wound healing. Adv Fiber Mater. 2022;4:1027–57. 10.1007/s42765-022-00178-z.

[102] Zhang X, Lv R, Chen L, Sun R, Zhang Y, Sheng R, Du T, Li Y, Qi Y. a multifunctional janus electrospun nanofiber dressing with biofluid draining, monitoring, and antibacterial properties for wound healing. ACS Appl Mater Interfaces. 2022;14:12984–3000. 10.1021/acsami.1c22629. 35266385 10.1021/acsami.1c22629

[103] Park H, Patil TV, Dutta SD, Lee J, Ganguly K, Randhawa A, Kim H, Lim K. Extracellular matrix-bioinspired anisotropic topographical cues of electrospun nanofibers: A strategy of wound healing through macrophage polarization. Adv Healthc Mater. 2024;13:2304114. 10.1002/adhm.202304114. 10.1002/adhm.202304114 38295299

[104] Zhou J, Zheng J, Wang C, Zhang G, Yang H, Xiong F, Fan M, Wang Z, Li Y, Yang C. Electrospun biosensors for biomarker detection. Colloid Interface Sci Commun. 2024;59:100767. 10.1016/j.colcom.2024.100767.

[105] Maleki F, Razmi H, Rashidi M-R, Yousefi M, Ramezani S, Ghorbani M. Electrospun EU/HPMC nanofibers decorated by ZIF-8 nanoparticle as the advanced electrochemical biosensor modifier for sensitive and selective detection of c-MET cancer biomarker in human plasma sample. Biosens Bioelectron. 2024;257:116319. 10.1016/j.bios.2024.116319. 38669845 10.1016/j.bios.2024.116319

[106] Yildirim-Tirgil N, Akkoyun S, Atan HU, Bozkurt B. Development of a polypyrrole-chitosan electrospun nanofiber-based enzymatic biosensor for sensitive and rapid detection of acetylcholine. ACS Appl Polym Mater. 2025;7:611–21. 10.1021/acsapm.4c02614.

[107] Anusiya G, Jaiganesh R. A review on fabrication methods of nanofibers and a special focus on application of cellulose nanofibers. Carbohydr Polym Technol Appl. 2022;4:100262. 10.1016/j.carpta.2022.100262.

[108] Keirouz A, Wang Z, Reddy VS, Nagy ZK, Vass P, Buzgo M, Ramakrishna S, Radacsi N. The history of electrospinning: past, present, and future developments. Adv Mater Technol. 2023;8:2201723. 10.1002/admt.202201723.

[109] Ahmadi Bonakdar M, Rodrigue D. Electrospinning: Processes Structures, and Materials. Macromol. 2024;4:58–103. 10.3390/macromol4010004.

[110] Riaz A, Gidvall S, Prgomet Z, Hernandez AR, Ruzgas T, Nilsson EJ, Davies J, Valetti S. Three-dimensional oral mucosal equivalents as models for transmucosal drug permeation studies. Pharmaceutics. 2023;15:1513. 10.3390/pharmaceutics15051513. 37242755 10.3390/pharmaceutics15051513 PMC10223481

[111] Zheng Y, Xing L, Chen L, Zhou R, Wu J, Zhu X, Li L, Xiang Y, Wu R, Zhang L, Huang Y. Tailored elasticity combined with biomimetic surface promotes nanoparticle transcytosis to overcome mucosal epithelial barrier. Biomaterials. 2020;262:120323. 10.1016/j.biomaterials.2020.120323. 32896816 10.1016/j.biomaterials.2020.120323

[112] Butnarasu C, Petrini P, Bracotti F, Visai L, Guagliano G, Fiorio Pla A, Sansone E, Petrillo S, Visentin S. Mucosomes: Intrinsically mucoadhesive glycosylated mucin nanoparticles as multi-drug delivery platform. Adv Healthc Mater. 2022;11:2200340. 10.1002/adhm.202200340. 35608152 10.1002/adhm.202200340 PMC11468529

[113] Pérez-González GL, Villarreal-Gómez LJ, Serrano-Medina A, Torres-Martínez EJ, Cornejo-Bravo JM. <p>Mucoadhesive electrospun nanofibers for drug delivery systems: applications of polymers and the parameters’ roles</p>. Int J Nanomedicine. 2019;14:5271–85. 10.2147/IJN.S193328. 31409989 10.2147/IJN.S193328 PMC6643962

[114] Chan KC, Sadaf A, Gerrit Korvink J, Wenzel W. Electromechanical analysis of electrospun polymer fiber deposition. J Appl Phys. 2023:134. 10.1063/5.0171903.

[115] Castillo-Henríquez L, Vargas-Zúñiga R, Pacheco-Molina J, Vega-Baudrit J. Electrospun nanofibers: A nanotechnological approach for drug delivery and dissolution optimization in poorly water-soluble drugs. ADMET DMPK. 2020. 10.5599/admet.844. 35300196 10.5599/admet.844 PMC8915594

[116] Modgill V, Garg T, Goyal AK, Rath G. Permeability study of ciprofloxacin from ultra-thin nanofibrous film through various mucosal membranes. Artif Cells Nanomed Biotechnol. 2016;44:122–7. 10.3109/21691401.2014.924007. 24915047 10.3109/21691401.2014.924007

[117] Xu H, Li H, Chang J. Controlled drug release from a polymer matrix by patterned electrospun nanofibers with controllable hydrophobicity. J Mater Chem B. 2013;1:4182. 10.1039/c3tb20404a. 32260972 10.1039/c3tb20404a

[118] Paaver U, Heinämäki J, Laidmäe I, Lust A, Kozlova J, Sillaste E, Kirsimäe K, Veski P, Kogermann K. Electrospun nanofibers as a potential controlled-release solid dispersion system for poorly water-soluble drugs. Int J Pharm. 2015;479:252–60. 10.1016/j.ijpharm.2014.12.024. 25549852 10.1016/j.ijpharm.2014.12.024

[119] Kajdič S, Planinšek O, Gašperlin M, Kocbek P. Electrospun nanofibers for customized drug-delivery systems. J Drug Deliv Sci Technol. 2019;51:672–81. 10.1016/j.jddst.2019.03.038.

[120] Uhljar LÉ, Kan SY, Radacsi N, Koutsos V, Szabó-Révész P, Ambrus R. In vitro drug release permeability, and structural test of ciprofloxacin-loaded nanofibers. Pharmaceutics. 2021;13:556. 10.3390/pharmaceutics13040556. 33921031 10.3390/pharmaceutics13040556 PMC8071406

[121] Wang Y, Yu D-G, Liu Y, Liu Y-N. Progress of electrospun nanofibrous carriers for modifications to drug release profiles. J Funct Biomater. 2022;13:289. 10.3390/jfb13040289. 36547549 10.3390/jfb13040289 PMC9787859

[122] Pattnaik S, Swain K, Ramakrishna S. Optimal delivery of poorly soluble drugs using electrospun nanofiber technology: Challenges, state of the art, and future directions. WIREs Nanomed Nanobiotechnol. 2023;15:e1859. 10.1002/wnan.1859. 10.1002/wnan.1859 36193733

[123] Sharma Y, Chahar K, Mishra L, Kumari L, Singla A, Patel P, Singh D, Das Kurmi B. Recent overviews on the drug delivery aspects and applications of brinzolamide for the management of glaucoma. Health Sci Rev. 2023;6:100083. 10.1016/j.hsr.2023.100083.

[124] Mascarenhas M, Chaudhari P, Lewis SA. Natamycin ocular delivery: Challenges and advancements in ocular therapeutics. Adv Ther. 2023;40:3332–59. 10.1007/s12325-023-02541-x. 37289410 10.1007/s12325-023-02541-x PMC10329963

[125] Tsatsos M, MacGregor C, Athanasiadis I, Moschos MM, Hossain P, Anderson D. Herpes simplex virus keratitis: an update of the pathogenesis and current treatment with oral and topical antiviral agents. Clin Exp Ophthalmol. 2016;44:824–37. 10.1111/ceo.12785. 27273328 10.1111/ceo.12785

[126] Joy N, Venugopal D, Samavedi S. Robust strategies to reduce burst and achieve tunable control over extended drug release from uniaxially electrospun composites. Eur Polym J. 2022;168:111102. 10.1016/j.eurpolymj.2022.111102.

[127] Liu Y, Chen X, Gao Y, Yu D-G, Liu P. Elaborate design of shell component for manipulating the sustained release behavior from core–shell nanofibres. J Nanobiotechnology. 2022;20:244. 10.1186/s12951-022-01463-0. 35643572 10.1186/s12951-022-01463-0 PMC9148457

[128] Schoeller J, Itel F, Wuertz-Kozak K, Gaiser S, Luisier N, Hegemann D, Ferguson SJ, Fortunato G, Rossi RM. pH-responsive chitosan/alginate polyelectrolyte complexes on electrospun PLGA nanofibers for controlled drug release. Nanomaterials. 2021;11:1850. 10.3390/nano11071850. 34361236 10.3390/nano11071850 PMC8308421

[129] Ramos C, Lanno G-M, Laidmäe I, Meos A, Härmas R, Kogermann K. High humidity electrospinning of porous fibers for tuning the release of drug delivery systems. Int J Polym Mater Polym Biomater. 2021;70:880–92. 10.1080/00914037.2020.1765361.

[130] Hou J, Yang J, Zheng X, Wang M, Liu Y, Yu D-G. A nanofiber-based drug depot with high drug loading for sustained release. Int J Pharm. 2020;583:119397. 10.1016/j.ijpharm.2020.119397. 32376443 10.1016/j.ijpharm.2020.119397

[131] Tan SM, Teoh XY, Le Hwang J, Khong ZP, Sejare R, Almashhadani AQ, Assi RA, Chan SY. Electrospinning and its potential in fabricating pharmaceutical dosage form. J Drug Deliv Sci Technol. 2022;76:103761. 10.1016/j.jddst.2022.103761.

[132] Li Y, Zhu J, Cheng H, Li G, Cho H, Jiang M, Gao Q, Zhang X. Developments of advanced electrospinning techniques: A critical review. Adv Mater Technol. 2021;6:2100410. 10.1002/admt.202100410.

[133] Wen X, Xiong J, Lei S, Wang L, Qin X. Diameter refinement of electrospun nanofibers: from mechanism strategies to applications. Adv Fiber Mater. 2022;4:145–61. 10.1007/s42765-021-00113-8.

[134] Avossa J, Herwig G, Toncelli C, Itel F, Rossi RM. Electrospinning based on benign solvents: current definitions, implications and strategies. Green Chem. 2022;24:2347–75. 10.1039/D1GC04252A.

[135] Odularu AT. Basic principles of electrospinning mechanisms, nanofibre production, and anticancer drug delivery. J Chem. 2022;2022:1–15. 10.1155/2022/9283325.

[136] Kumar Sharma G, Rachel James N. Electrospinning: The technique and applications. In: Recent Developments in Nanofibers Research, IntechOpen. 2023. 10.5772/intechopen.105804.

[137] Xue J, Wu T, Dai Y, Xia Y. Electrospinning and electrospun nanofibers: methods, materials, and applications. Chem Rev. 2019;119:5298–415. 10.1021/acs.chemrev.8b00593. 30916938 10.1021/acs.chemrev.8b00593 PMC6589095

[138] Akdere M, Schneiders T. Modeling of the electrospinning process. In: Advances in Modeling and Simulation in Textile Engineering, Elsevier. 2021:237–253. 10.1016/B978-0-12-822977-4.00015-7.

[139] Ghaderpour A, Hoseinkhani Z, Yarani R, Mohammadiani S, Amiri F, Mansouri K. Altering the characterization of nanofibers by changing the electrospinning parameters and their application in tissue engineering, drug delivery, and gene delivery systems. Polym Adv Technol. 2021;32:1924–50. 10.1002/pat.5242.

[140] Guo Y, Wang X, Shen Y, Dong K, Shen L, Alzalab AAA. Research progress, models and simulation of electrospinning technology: a review. J Mater Sci. 2022;57:58–104. 10.1007/s10853-021-06575-w. 34658418 10.1007/s10853-021-06575-w PMC8513391

[141] Huang L, Bui N, Manickam SS, McCutcheon JR. Controlling electrospun nanofiber morphology and mechanical properties using humidity. J Polym Sci B Polym Phys. 2011;49:1734–44. 10.1002/polb.22371.

[142] Mazoochi T, Hamadanian M, Ahmadi M, Jabbari V. Investigation on the morphological characteristics of nanofiberous membrane as electrospun in the different processing parameters. Int J Ind Chem. 2012;3:2. 10.1186/2228-5547-3-2.

[143] Yang G-Z, Li H-P, Yang J-H, Wan J, Yu D-G. Influence of working temperature on the formation of electrospun polymer nanofibers. Nanoscale Res Lett. 2017;12:55. 10.1186/s11671-016-1824-8. 28105604 10.1186/s11671-016-1824-8 PMC5247380

[144] Deshawar D, Gupta K, Chokshi P. Electrospinning of polymer solutions: An analysis of instability in a thinning jet with solvent evaporation. Polymer (Guildf). 2020;202:122656. 10.1016/j.polymer.2020.122656.

[145] Demirtaş MS, Saha MC. Engineering highly aligned continuous nanofibers via electrospinning: A comprehensive study on collector design, electrode geometry, and collector speed. Express Polym Lett. 2024;18:851–67. 10.3144/expresspolymlett.2024.63.

[146] Faizal F, Al-Fikri AM, Abdurrochman A, Joni IM, Panatarani C. Development of precision pump and high voltage DC-regulator for electrospinning apparatus: experimental test with preparation of PVA microfiber. J Phys Conf Ser. 2020;1568:012006. 10.1088/1742-6596/1568/1/012006.

[147] Abu Owida H, Al-haj Moh’d B, Al Takrouri M. Designing an Integrated Low-cost Electrospinning Device for Nanofibrous Scaffold Fabrication. HardwareX. 2022;11:e00250. 10.1016/j.ohx.2021.e00250. 35509902 10.1016/j.ohx.2021.e00250 PMC9058581

[148] Yusro M, Kadarisman K. Development of low-cost electrospinning to fabricate structured nanofiber for biomedical designs with manageable flowrate and voltage. Indonesian J Electron Electromed Eng Med Informatics. 2022:4. 10.35882/ijeeemi.v4i3.234.

[149] de la Rosa Gatica JA, Martínez Hernández AL, Vázquez-Nava N, García-Casillas PE, Velasco-Santos C. Design, development, and experimental setup of near-field electrospinning with a sharp electrode: Influence of procedural parameters on the 3D nanofiber structure. Rev Sci Instrum. 2022:93. 10.1063/5.0065101. 10.1063/5.0065101 35104972

[150] Morkus P, Sibbald S, Choi L, Rassenberg S, Filipe CDM, Latulippe DR. Miniaturization of an enclosed electrospinning process to enhance reproducibility in the fabrication of rapidly dissolving cell-based biosensors. Biotechnol J. 2024;19:2300306. 10.1002/biot.202300306. 10.1002/biot.202300306 37882254

[151] Can-Herrera LA, Oliva AI, Dzul-Cervantes MAA, Pacheco-Salazar OF, Cervantes-Uc JM. Morphological and mechanical properties of electrospun polycaprolactone scaffolds: Effect of applied voltage. Polymers (Basel). 2021;13:662. 10.3390/polym13040662. 33672211 10.3390/polym13040662 PMC7926916

[152] Sivan M, Madheswaran D, Valtera J, Kostakova EK, Lukas D. Alternating current electrospinning: The impacts of various high-voltage signal shapes and frequencies on the spinnability and productivity of polycaprolactone nanofibers. Mater Des. 2022;213:110308. 10.1016/j.matdes.2021.110308.

[153] Tarus B, Fadel N, Al-Oufy A, El-Messiry M. Effect of polymer concentration on the morphology and mechanical characteristics of electrospun cellulose acetate and poly (vinyl chloride) nanofiber mats. Alex Eng J. 2016;55:2975–84. 10.1016/j.aej.2016.04.025.

[154] Topuz F, Uyar T. Electrospinning of cyclodextrin nanofibers: The effect of process parameters. J Nanomater. 2020;2020:1–10. 10.1155/2020/7529306.

[155] Bakar SSS, Fong KC, Eleyas A, Nazeri MFM. Effect of voltage and flow rate electrospinning parameters on polyacrylonitrile electrospun fibers. IOP Conf Ser Mater Sci Eng. 2018;318:012076. 10.1088/1757-899X/318/1/012076.

[156] Mohammadi M, Mohammadi N, Mehdipour-Ataei S. On the preparation of thin nanofibers of polysulfone polyelectrolyte for improving conductivity of proton-exchange membranes by electrospinning: Taguchi design, response surface methodology, and genetic algorithm. Int J Hydrogen Energy. 2020;45:34110–24. 10.1016/j.ijhydene.2020.09.125.

[157] Haider A, Haider S, Kang I-K. A comprehensive review summarizing the effect of electrospinning parameters and potential applications of nanofibers in biomedical and biotechnology. Arab J Chem. 2018;11:1165–88. 10.1016/j.arabjc.2015.11.015.

[158] Fatimah I, Sari TI, Anggoro D. Effect of concentration and nozzle-collector distance on the morphology of nanofibers. Key Eng Mater. 2020;860:315–9. 10.4028/www.scientific.net/KEM.860.315.

[159] Angammana CJ, Jayaram SH. Analysis of the effects of solution conductivity on electrospinning process and fiber morphology. IEEE Trans Ind Appl. 2011;47:1109–17. 10.1109/TIA.2011.2127431.

[160] Lasprilla-Botero J, Álvarez-Láinez M, Lagaron JM. The influence of electrospinning parameters and solvent selection on the morphology and diameter of polyimide nanofibers. Mater Today Commun. 2018;14:1–9. 10.1016/j.mtcomm.2017.12.003.

[161] Mailley D, Hébraud A, Schlatter G. A Review on the Impact of Humidity during Electrospinning: From the Nanofiber Structure Engineering to the Applications. Macromol Mater Eng. 2021;306:2100115. 10.1002/mame.202100115.

[162] Chinnappan BA, Krishnaswamy M, Xu H, Hoque ME. Electrospinning of biomedical nanofibers/nanomembranes: Effects of process parameters. Polymers (Basel). 2022;14:3719. 10.3390/polym14183719. 36145868 10.3390/polym14183719 PMC9504486

[163] Huang W, Xiao Y, Shi X. Construction of electrospun organic/inorganic hybrid nanofibers for drug delivery and tissue engineering applications. Adv Fiber Mater. 2019;1:32–45. 10.1007/s42765-019-00007-w.

[164] Zare P, Pezeshki-Modaress M, Davachi SM, Zare P, Yazdian F, Simorgh S, Ghanbari H, Rashedi H, Bagher Z. Alginate sulfate-based hydrogel/nanofiber composite scaffold with controlled Kartogenin delivery for tissue engineering. Carbohydr Polym. 2021;266:118123. 10.1016/j.carbpol.2021.118123. 34044939 10.1016/j.carbpol.2021.118123

[165] Rajati H, Alvandi H, Rahmatabadi SS, Hosseinzadeh L, Arkan E. A nanofiber-hydrogel composite from green synthesized AgNPs embedded to PEBAX/PVA hydrogel and PA/Pistacia atlantica gum nanofiber for wound dressing. Int J Biol Macromol. 2023;226:1426–43. 10.1016/j.ijbiomac.2022.11.255. 36442567 10.1016/j.ijbiomac.2022.11.255

[166] Huang Y, Song M, Li X, Du Y, Gao Z, Zhao Y-Q, Li C, Yan H, Mo X, Wang C, Hou G, Xie X. Temperature-responsive self-contraction nanofiber/hydrogel composite dressing facilitates the healing of diabetic-infected wounds. Mater Today Bio. 2024;28:101214. 10.1016/j.mtbio.2024.101214. 39280109 10.1016/j.mtbio.2024.101214 PMC11402428

[167] Sun L, Zhou J, Chen Y, Yu D-G, Liu P. A combined electrohydrodynamic atomization method for preparing nanofiber/microparticle hybrid medicines. Front Bioeng Biotechnol. 2023;11:1308004. 10.3389/fbioe.2023.1308004. 38033817 10.3389/fbioe.2023.1308004 PMC10684662

[168] Zhou J, Dai Y, Fu J, Yan C, Yu D-G, Yi T. Dual-step controlled release of berberine hydrochloride from the trans-scale hybrids of nanofibers and microparticles. Biomolecules. 2023;13:1011. 10.3390/biom13061011. 37371591 10.3390/biom13061011 PMC10295831

[169] Ajalloueian F, Eklund Thamdrup LH, Mazzoni C, Petersen RS, Keller SS, Boisen A. High-yield fabrication of monodisperse multilayer nanofibrous microparticles for advanced oral drug delivery applications. Heliyon. 2024;10:e30844. 10.1016/j.heliyon.2024.e30844. 38799753 10.1016/j.heliyon.2024.e30844 PMC11126835

[170] Tuğcu-Demiröz F, Saar S, Kara AA, Yıldız A, Tunçel E, Acartürk F. Development and characterization of chitosan nanoparticles loaded nanofiber hybrid system for vaginal controlled release of benzydamine. Eur J Pharm Sci. 2021;161:105801. 10.1016/j.ejps.2021.105801. 33691155 10.1016/j.ejps.2021.105801

[171] Mohamady Hussein MA, Guler E, Rayaman E, Cam ME, Sahin A, Grinholc M, Sezgin Mansuroglu D, Sahin YM, Gunduz O, Muhammed M, El-Sherbiny IM, Megahed M. Dual-drug delivery of Ag-chitosan nanoparticles and phenytoin via core-shell PVA/PCL electrospun nanofibers. Carbohydr Polym. 2021;270:118373. 10.1016/j.carbpol.2021.118373. 34364617 10.1016/j.carbpol.2021.118373

[172] Heydari Foroushani P, Rahmani E, Alemzadeh I, Vossoughi M, Pourmadadi M, Rahdar A, Díez-Pascual AM. Curcumin sustained release with a hybrid chitosan-silk fibroin nanofiber containing silver nanoparticles as a novel highly efficient antibacterial wound dressing. Nanomaterials. 2022;12:3426. 10.3390/nano12193426. 36234554 10.3390/nano12193426 PMC9565735

[173] Heidari M, Bahrami SH, Ranjbar-Mohammadi M, Milan PB. Smart electrospun nanofibers containing PCL/gelatin/graphene oxide for application in nerve tissue engineering. Mater Sci Eng C. 2019;103:109768. 10.1016/j.msec.2019.109768. 10.1016/j.msec.2019.109768 31349413

[174] Bei HP, Yang Y, Zhang Q, Tian Y, Luo X, Yang M, Zhao X. Graphene-based nanocomposites for neural tissue engineering. Molecules. 2019;24:658. 10.3390/molecules24040658. 30781759 10.3390/molecules24040658 PMC6413135

[175] Muñoz-Gonzalez AM, Leal-Marin S, Clavijo-Grimaldo D, Glasmacher B. Graphene-enhanced PCL electrospun nanofiber scaffolds for cardiac tissue engineering. Int J Artif Organs. 2024;47:633–41. 10.1177/03913988241266088. 39113566 10.1177/03913988241266088 PMC11487899

[176] Zhang M, Song W, Tang Y, Xu X, Huang Y, Yu D. Polymer-based nanofiber-nanoparticle hybrids and their medical applications. Polymers (Basel). 2022;14:351. 10.3390/polym14020351. 35054758 10.3390/polym14020351 PMC8780324

[177] Wang Y, Qiao W, Wang B, Zhang Y, Shao P, Yin T. Electrospun composite nanofibers containing nanoparticles for the programmable release of dual drugs. Polym J. 2011;43:478–83. 10.1038/pj.2011.11.

[178] Kong H, Jang J. Antibacterial properties of novel poly(methyl methacrylate) nanofiber containing silver nanoparticles. Langmuir. 2008;24:2051–6. 10.1021/la703085e. 18225933 10.1021/la703085e

[179] He H, Wu M, Zhu J, Yang Y, Ge R, Yu D-G. Engineered spindles of little molecules around electrospun nanofibers for biphasic drug release. Adv Fiber Mater. 2022;4:305–17. 10.1007/s42765-021-00112-9.

[180] Tonsomboon K, Butcher AL, Oyen ML. Strong and tough nanofibrous hydrogel composites based on biomimetic principles. Mater Sci Eng, C. 2017;72:220–7. 10.1016/j.msec.2016.11.025. 10.1016/j.msec.2016.11.025 28024580

[181] Xiao L, Liu H, Huang H, Wu S, Xue L, Geng Z, Cai L, Yan F. 3D nanofiber scaffolds from 2D electrospun membranes boost cell penetration and positive host response for regenerative medicine. J Nanobiotechnology. 2024;22:322. 10.1186/s12951-024-02578-2. 38849858 10.1186/s12951-024-02578-2 PMC11162076

[182] Hezarkhani M, Aliyeva N, Menceloglu YZ, Saner Okan B. Fabrication Methodologies of Multi-layered and Multi-functional Electrospun Structures by Co-axial and Multi-axial Electrospinning Techniques. In: Electrospun Nanofibers, Springer International Publishing, Cham. 2022:35–66. 10.1007/978-3-030-99958-2_2.

[183] Tabakoglu S, Kołbuk D, Sajkiewicz P. Multifluid electrospinning for multi-drug delivery systems: pros and cons, challenges, and future directions. Biomater Sci. 2023;11:37–61. 10.1039/D2BM01513G. 10.1039/d2bm01513g 36367316

[184] Ding Y, Dou C, Chang S, Xie Z, Yu D-G, Liu Y, Shao J. Core-shell eudragit S100 nanofibers prepared via triaxial electrospinning to provide a colon-targeted extended drug release. Polymers (Basel). 2020;12:2034. 10.3390/polym12092034. 32906728 10.3390/polym12092034 PMC7565919

[185] Ghosal K, Augustine R, Zaszczynska A, Barman M, Jain A, Hasan A, Kalarikkal N, Sajkiewicz P, Thomas S. Novel drug delivery systems based on triaxial electrospinning based nanofibers. React Funct Polym. 2021;163:104895. 10.1016/j.reactfunctpolym.2021.104895.

[186] Karimi Afshar S, Abdorashidi M, Dorkoosh FA, Akbari Javar H. Electrospun fibers: Versatile approaches for controlled release applications. Int J Polym Sci. 2022;2022:1–17. 10.1155/2022/9116168.

[187] Nyamweya NN. Applications of polymer blends in drug delivery. Futur J Pharm Sci. 2021;7:18. 10.1186/s43094-020-00167-2.

[188] Lin ST, Kimble L, Bhattacharyya D. Polymer blends and composites for biomedical applications. In: Biomaterials for Implants and Scaffolds, 1st ed., Springer. 2017:195–235. 10.1007/978-3-662-53574-5_7.

[189] Nawn G, Vezzù K, Negro E, Pace G, Park JW, Wycisk R, Cavinato G, Pintauro PN, Di Noto V. Structural analyses of blended Nafion/PVDF electrospun nanofibers. Phys Chem Chem Phys. 2019;21:10357–69. 10.1039/C9CP01891C. 31074475 10.1039/c9cp01891c

[190] Zarrintaj P, Saeb MR, Jafari SH, Mozafari M. Application of compatibilized polymer blends in biomedical fields. In: Compatibilization of Polymer Blends, Elsevier. 2020:511–537. 10.1016/B978-0-12-816006-0.00018-9.

[191] Spontak RJ, Ryan JJ. Polymer blend compatibilization by the addition of block copolymers. In: Compatibilization of Polymer Blends, Elsevier. 2020:57–102. 10.1016/B978-0-12-816006-0.00003-7.

[192] Fortelný I, Jůza J. The effects of copolymer compatibilizers on the phase structure evolution in polymer blends—a review. Materials. 2021;14:7786. 10.3390/ma14247786. 34947377 10.3390/ma14247786 PMC8707745

[193] Goonoo N, Bhaw-Luximon A, Jhurry D. Biodegradable polymer blends: miscibility, physicochemical properties and biological response of scaffolds. Polym Int. 2015;64:1289–302. 10.1002/pi.4937.

[194] Behtaj S, Karamali F, Masaeli E, Anissimov YG, Rybachuk M. Electrospun PGS/PCL, PLLA/PCL, PLGA/PCL and pure PCL scaffolds for retinal progenitor cell cultivation. Biochem Eng J. 2021;166:107846. 10.1016/j.bej.2020.107846.

[195] Asano N, Sugihara S, Suye S, Fujita S. Electrospun porous nanofibers with imprinted patterns induced by phase separation of immiscible polymer blends. ACS Omega. 2022;7:19997–20005. 10.1021/acsomega.2c01798. 35721947 10.1021/acsomega.2c01798 PMC9202247

[196] Ghasemiyeh P, Mohammadi-Samani S. Polymers Blending as Release Modulating Tool in Drug Delivery. Front Mater. 2021;8:752813. 10.3389/fmats.2021.752813.

[197] Sionkowska A. Current research on the blends of natural and synthetic polymers as new biomaterials: Review. Prog Polym Sci. 2011;36:1254–76. 10.1016/j.progpolymsci.2011.05.003.

[198] Tahir M, Vicini S, Sionkowska A. Electrospun materials based on polymer and biopolymer blends—a review. Polymers (Basel). 2023;15:1654. 10.3390/polym15071654. 37050268 10.3390/polym15071654 PMC10096894

[199] Stie MB, Gätke JR, Wan F, Chronakis IS, Jacobsen J, Nielsen HM. Swelling of mucoadhesive electrospun chitosan/polyethylene oxide nanofibers facilitates adhesion to the sublingual mucosa. Carbohydr Polym. 2020;242:116428. 10.1016/j.carbpol.2020.116428. 32564847 10.1016/j.carbpol.2020.116428

[200] Desai N, Rana D, Salave S, Gupta R, Patel P, Karunakaran B, Sharma A, Giri J, Benival D, Kommineni N. Chitosan: A potential biopolymer in drug delivery and biomedical applications. Pharmaceutics. 2023;15:1313. 10.3390/pharmaceutics15041313. 37111795 10.3390/pharmaceutics15041313 PMC10144389

[201] Ahmad K, Zhang Y, Chen P, Yang X, Hou H. Chitosan interaction with stomach mucin layer to enhances gastric retention and mucoadhesive properties. Carbohydr Polym. 2024;333:121926. 10.1016/j.carbpol.2024.121926. 38494203 10.1016/j.carbpol.2024.121926

[202] Abu Elella MH, Kolawole OM. Recent advances in modified chitosan-based drug delivery systems for transmucosal applications: A comprehensive review. Int J Biol Macromol. 2024;277:134531. 10.1016/j.ijbiomac.2024.134531. 39116977 10.1016/j.ijbiomac.2024.134531

[203] Kotla NG, Mohd Isa IL, Larrañaga A, Maddiboyina B, Swamy SK, Sivaraman G, Vemula PK. Hyaluronic acid-based bioconjugate systems, scaffolds, and their therapeutic potential. Adv Healthc Mater. 2023;12:2203104. 10.1002/adhm.202203104. 10.1002/adhm.202203104 36972409

[204] Guarise C, Acquasaliente L, Pasut G, Pavan M, Soato M, Garofolin G, Beninatto R, Giacomel E, Sartori E, Galesso D. The role of high molecular weight hyaluronic acid in mucoadhesion on an ocular surface model. J Mech Behav Biomed Mater. 2023;143:105908. 10.1016/j.jmbbm.2023.105908. 37209594 10.1016/j.jmbbm.2023.105908

[205] Ponedel’kina YI, Khaybrakhmanova EA, Yu LR. In vitro evaluation of the mucoadhesive properties of zinc hyaluronate. Nat Prod Res. 2025:1–5. 10.1080/14786419.2025.2463693. 10.1080/14786419.2025.2463693 39933058

[206] Mfoafo K, Mittal R, Eshraghi A, Omidi Y, Omidian H. Thiolated polymers: An overview of mucoadhesive properties and their potential in drug delivery via mucosal tissues. J Drug Deliv Sci Technol. 2023;85: 104596. 10.1016/j.jddst.2023.104596.

[207] Puri V, Sharma A, Kumar P, Singh I. Thiolation of biopolymers for developing drug delivery systems with enhanced mechanical and mucoadhesive properties: A review. Polymers (Basel). 2020;12:1803. 10.3390/polym12081803. 32796741 10.3390/polym12081803 PMC7464630

[208] Fürst A, Kali G, Dizdarević A, Stengel D, Bernkop-Schnürch A. Mucoadhesive polymers: Design of S-protected thiolated cyclodextrin-based hydrogels. Int J Pharm. 2024;656:124075. 10.1016/j.ijpharm.2024.124075. 38599445 10.1016/j.ijpharm.2024.124075

[209] Rosiak P, Latanska I, Paul P, Sujka W, Kolesinska B. Modification of alginates to modulate their physic-chemical properties and obtain biomaterials with different functional properties. Molecules. 2021;26:7264. 10.3390/molecules26237264. 34885846 10.3390/molecules26237264 PMC8659150

[210] Zhang Z, Liu H, Yu D-G, Bligh S-WA. Alginate-based electrospun nanofibers and the enabled drug controlled release profiles: A review. Biomolecules. 2024;14:789. 10.3390/biom14070789. 39062503 10.3390/biom14070789 PMC11274620

[211] Qosim N, Dai Y, Williams GR, Edirisinghe M. Structure, properties, forming, and applications of alginate fibers: A review. Int Mater Rev. 2024;69:309–33. 10.1177/09506608241280419.

[212] Hasanin MS. Cellulose-based biomaterials: Chemistry and biomedical applications. Starch - Stärke. 2022;74:2200060. 10.1002/star.202200060.

[213] Chang Y, Zhao W, Li W, Zhang Q, Wang G. Bioadhesive and drug-loaded cellulose nanofiber/alginate film for healing oral mucosal wounds. Int J Biol Macromol. 2024;276:133858. 10.1016/j.ijbiomac.2024.133858. 39009262 10.1016/j.ijbiomac.2024.133858

[214] Ferreira JO, Zambuzi GC, Camargos CHM, Carvalho ACW, Ferreira MP, Rezende CA, de Freitas O, Francisco KR. Zein and hydroxypropyl methylcellulose acetate succinate microfibers combined with metronidazole benzoate and/or metronidazole-incorporated cellulose nanofibrils for potential periodontal treatment. Int J Biol Macromol. 2024;261:129701. 10.1016/j.ijbiomac.2024.129701. 38280709 10.1016/j.ijbiomac.2024.129701

[215] Noreen A, Nazli Z-H, Akram J, Rasul I, Mansha A, Yaqoob N, Iqbal R, Tabasum S, Zuber M, Zia KM. Pectins functionalized biomaterials; a new viable approach for biomedical applications: A review. Int J Biol Macromol. 2017;101:254–72. 10.1016/j.ijbiomac.2017.03.029. 28300586 10.1016/j.ijbiomac.2017.03.029

[216] Khorasani AC, Shojaosadati SA. Pectin-non-starch nanofibers biocomposites as novel gastrointestinal-resistant prebiotics. Int J Biol Macromol. 2017;94:131–44. 10.1016/j.ijbiomac.2016.10.011. 27720960 10.1016/j.ijbiomac.2016.10.011

[217] Laurén P, Paukkonen H, Lipiäinen T, Dong Y, Oksanen T, Räikkönen H, Ehlers H, Laaksonen P, Yliperttula M, Laaksonen T. Pectin and mucin enhance the bioadhesion of drug loaded nanofibrillated cellulose films. Pharm Res. 2018;35:145. 10.1007/s11095-018-2428-z. 29790010 10.1007/s11095-018-2428-z

[218] Lam HT, Zupančič O, Laffleur F, Bernkop-Schnürch A. Mucoadhesive properties of polyacrylates: Structure – function relationship. Int J Adhes Adhes. 2021;107:102857. 10.1016/j.ijadhadh.2021.102857.

[219] Karthikeyan K, Sowjanya RS, Yugandhar ADV, Gopinath S, Korrapati PS. Design and development of a topical dosage form for the convenient delivery of electrospun drug loaded nanofibers. RSC Adv. 2015;5:52420–6. 10.1039/C5RA04438C.

[220] Hosseinzadeh S, Hamedi S, Esmaeili E, Kabiri M, Babaie A, Soleimani M, Ardeshirylajimi A. Mucoadhesive nanofibrous membrane with anti-inflammatory activity. Polym Bull. 2019;76:4827–40. 10.1007/s00289-018-2618-1.

[221] Zhong Y, Lin Q, Yu H, Shao L, Cui X, Pang Q, Zhu Y, Hou R. Construction methods and biomedical applications of PVA-based hydrogels. Front Chem. 2024;12:1376799. 10.3389/fchem.2024.1376799. 38435666 10.3389/fchem.2024.1376799 PMC10905748

[222] Samprasit W, Rojanarata T, Akkaramongkolporn P, Ngawhirunpat T, Kaomongkolgit R, Opanasopit P. Fabrication and in vitro/in vivo performance of mucoadhesive electrospun nanofiber mats containing α-mangostin. AAPS PharmSciTech. 2015;16:1140–52. 10.1208/s12249-015-0300-6. 25716329 10.1208/s12249-015-0300-6 PMC4674643

[223] Vashisth P, Raghuwanshi N, Srivastava AK, Singh H, Nagar H, Pruthi V. Ofloxacin loaded gellan/PVA nanofibers - Synthesis, characterization and evaluation of their gastroretentive/mucoadhesive drug delivery potential. Mater Sci Eng C. 2017;71:611–9. 10.1016/j.msec.2016.10.051. 10.1016/j.msec.2016.10.051 27987752

[224] Franco P, De Marco I. The use of poly(N-vinyl pyrrolidone) in the delivery of drugs: A review. Polymers (Basel). 2020;12:1114. 10.3390/polym12051114. 32414187 10.3390/polym12051114 PMC7285361

[225] Kurakula M, Koteswara Rao GSN. Moving polyvinyl pyrrolidone electrospun nanofibers and bioprinted scaffolds toward multidisciplinary biomedical applications. Eur Polym J. 2020;136:109919. 10.1016/j.eurpolymj.2020.109919.

[226] Paczkowska-Walendowska M, Szymanowska D, Cielecka-Piontek J. Mechanochemical properties of mucoadhesive tablets based on PVP/HPβCD electrospun nanofibers as local delivery of polygoni cuspidati extract for treating oral infections. Pharmaceuticals. 2023;16:579. 10.3390/ph16040579. 37111336 10.3390/ph16040579 PMC10145533

[227] Chou S-F, Woodrow KA. Relationships between mechanical properties and drug release from electrospun fibers of PCL and PLGA blends. J Mech Behav Biomed Mater. 2017;65:724–33. 10.1016/j.jmbbm.2016.09.004. 27756048 10.1016/j.jmbbm.2016.09.004 PMC6461716

[228] Guidotti G, Soccio M, Bondi E, Posati T, Sotgiu G, Zamboni R, Torreggiani A, Corticelli F, Lotti N, Aluigi A. Effects of the blending ratio on the design of keratin/poly(butylene succinate) nanofibers for drug delivery applications. Biomolecules. 2021;11:1194. 10.3390/biom11081194. 34439860 10.3390/biom11081194 PMC8392087

[229] Jahanmardi Y, Tavanaie MA, Tehrani-Bagha AR. Curcumin release from blended polycaprolactone/polylactic acid electrospun nanofibrous meshes. J Ind Text. 2021;50:1065–78. 10.1177/1528083719851845.

[230] Brako F, Thorogate R, Mahalingam S, Raimi-Abraham B, Craig DQM, Edirisinghe M. Mucoadhesion of progesterone-loaded drug delivery nanofiber constructs. ACS Appl Mater Interfaces. 2018;10:13381–9. 10.1021/acsami.8b03329. 29595052 10.1021/acsami.8b03329

[231] Brako F, Raimi-Abraham B, Mahalingam S, Craig DQM, Edirisinghe M. Making nanofibres of mucoadhesive polymer blends for vaginal therapies. Eur Polym J. 2015;70:186–96. 10.1016/j.eurpolymj.2015.07.006.

[232] Rohani Shirvan A, Hemmatinejad N, Bahrami SH, Bashari A. Fabrication of multifunctional mucoadhesive buccal patch for drug delivery applications. J Biomed Mater Res A. 2021;109:2640–56. 10.1002/jbm.a.37257. 34190400 10.1002/jbm.a.37257

[233] Jordan AM, Viswanath V, Kim S-E, Pokorski JK, Korley LTJ. Processing and surface modification of polymer nanofibers for biological scaffolds: a review. J Mater Chem B. 2016;4:5958–74. 10.1039/C6TB01303A. 32263485 10.1039/c6tb01303a

[234] Mertgen A-S, Trossmann VT, Guex AG, Maniura-Weber K, Scheibel T, Rottmar M. Multifunctional biomaterials: Combining material modification strategies for engineering of cell-contacting surfaces. ACS Appl Mater Interfaces. 2020;12:21342–67. 10.1021/acsami.0c01893. 32286789 10.1021/acsami.0c01893

[235] Sofi HS, Ashraf R, Khan AH, Beigh MA, Majeed S, Sheikh FA. Reconstructing nanofibers from natural polymers using surface functionalization approaches for applications in tissue engineering, drug delivery and biosensing devices. Mater Sci Eng C. 2019;94:1102–24. 10.1016/j.msec.2018.10.069. 10.1016/j.msec.2018.10.069 30423692

[236] Karthik C, Rajalakshmi S, Thomas S, Thomas V. Intelligent polymeric biomaterials surface driven by plasma processing. Curr Opin Biomed Eng. 2023;26:100440. 10.1016/j.cobme.2022.100440.

[237] Sivan M, Madheswaran D, Asadian M, Cools P, Thukkaram M, Van Der Voort P, Morent R, De Geyter N, Lukas D. Plasma treatment effects on bulk properties of polycaprolactone nanofibrous mats fabricated by uncommon AC electrospinning: A comparative study. Surf Coat Technol. 2020;399:126203. 10.1016/j.surfcoat.2020.126203.

[238] Das P, Ojah N, Kandimalla R, Mohan K, Gogoi D, Dolui SK, Choudhury AJ. Surface modification of electrospun PVA/chitosan nanofibers by dielectric barrier discharge plasma at atmospheric pressure and studies of their mechanical properties and biocompatibility. Int J Biol Macromol. 2018;114:1026–32. 10.1016/j.ijbiomac.2018.03.115. 29578008 10.1016/j.ijbiomac.2018.03.115

[239] Juárez-Moreno JA, Ávila-Ortega A, Oliva AI, Avilés F, Cauich-Rodríguez JV. Effect of wettability and surface roughness on the adhesion properties of collagen on PDMS films treated by capacitively coupled oxygen plasma. Appl Surf Sci. 2015;349:763–73. 10.1016/j.apsusc.2015.05.063.

[240] Li Z, Zeng R, Yang L, Ren X, Maffucci KG, Qu Y. Development and characterization of PCL electrospun membrane-coated bletilla striata polysaccharide-based gastroretentive drug delivery system. AAPS PharmSciTech. 2020;21:66. 10.1208/s12249-019-1607-5. 31932983 10.1208/s12249-019-1607-5

[241] Bhatt S, Pulpytel J, Arefi-Khonsari F. Low and atmospheric plasma polymerisation of nanocoatings for bio-applications. Surf Innov. 2015;3:63–83. 10.1680/sufi.14.00008.

[242] Iqbal M, Dinh D, Abbas Q, Imran M, Sattar H, Ul Ahmad A. Controlled surface wettability by plasma polymer surface modification. Surfaces. 2019;2:349–71. 10.3390/surfaces2020026.

[243] Sivri Ç. Development of electrospun nanofibers having novel morphologies via corona plasma treatment. J Achiev Mater Manuf Eng. 2016;76:30–40. 10.5604/17348412.1228632.

[244] Khamsen N, Akkarachanchainon A, Teerakawanich N, Srisonphan S. Organic and bio material surface modification via corona discharge induced atmospheric-cold plasma. Procedia Comput Sci. 2016;86:325–8. 10.1016/j.procs.2016.05.088.

[245] Dole N, Ahmadi K, Solanki D, Swaminathan V, Keswani V, Keswani M. Corona Treatment of Polymer Surfaces to Enhance Adhesion. In: Polymer Surface Modification to Enhance Adhesion, Wiley. 2024:45–76. 10.1002/9781394231034.ch2.

[246] Song W, Chen L, Seta J, Markel DC, Yu X, Ren W. Corona discharge: A novel approach to fabricate three-dimensional electrospun nanofibers for bone tissue engineering. ACS Biomater Sci Eng. 2017;3:1146–53. 10.1021/acsbiomaterials.7b00061. 33429589 10.1021/acsbiomaterials.7b00061

[247] Jaganathan SK, Balaji A, Vellayappan MV, Subramanian AP, John AA, Asokan MK, Supriyanto E. Review: Radiation-induced surface modification of polymers for biomaterial application. J Mater Sci. 2015;50:2007–18. 10.1007/s10853-014-8718-x.

[248] Luo J, Zhang M, Nie J, Liu G, Tan J, Yang B, Song S, Zhao JR. A deep insight into the structure and performance evolution of aramid nanofiber films induced by UV irradiation. Polym Degrad Stab. 2019;167:170–8. 10.1016/j.polymdegradstab.2019.07.001.

[249] Rabiatul AR, Lokanathan Y, Rohaina CM, Chowdhury SR, Aminuddin BS, Ruszymah BHI. Surface modification of electrospun poly(methyl methacrylate) (PMMA) nanofibers for the development of *in vitro* respiratory epithelium model. J Biomater Sci Polym Ed. 2015;26:1297–311. 10.1080/09205063.2015.1088183. 26335265 10.1080/09205063.2015.1088183

[250] Kulkarni D, Musale S, Panzade P, Paiva-Santos AC, Sonwane P, Madibone M, Choundhe P, Giram P, Cavalu S. Surface Functionalization of Nanofibers: The Multifaceted Approach for Advanced Biomedical Applications. Nanomaterials. 2022;12:3899. 10.3390/nano12213899. 36364675 10.3390/nano12213899 PMC9655053

[251] Minko S. Grafting on solid surfaces: “Grafting to” and “Grafting from” methods. In: Polymer Surfaces and Interfaces, Springer Berlin Heidelberg, Berlin, Heidelberg. 2008:215–234. 10.1007/978-3-540-73865-7_11.

[252] Sakhare MS, Rajput HH. Polymer grafting and applications in pharmaceutical drug delivery systems-a brief review. Asian J Pharm Clin Res. 2017;10:59. 10.22159/ajpcr.2017.v10i6.18072.

[253] Hua Z, Keogh R, Li Z, Wilks TR, Chen G, O’Reilly RK. Reversibly manipulating the surface chemistry of polymeric nanostructures via a “Grafting To” approach mediated by nucleobase interactions. Macromolecules. 2017;50:3662–70. 10.1021/acs.macromol.7b00286. 28529382 10.1021/acs.macromol.7b00286 PMC5435456

[254] Rosenthal A, Mantz A, Nguyen AL, Bittrich E, Schubert E, Schubert MM, Stamm M, Pannier AK, Uhlmann P. Biofunctionalization of titanium substrates using nanoscale polymer brushes with cell adhesion peptides. J Phys Chem B. 2018;122(25):6543–50. 10.1021/acs.jpcb.8b02407. 29878775 10.1021/acs.jpcb.8b02407

[255] Cui W, Li X, Xie C, Chen J, Zou J, Zhou S, Weng J. Controllable growth of hydroxyapatite on electrospun poly(dl-lactide) fibers grafted with chitosan as potential tissue engineering scaffolds. Polymer (Guildf). 2010;51:2320–8. 10.1016/j.polymer.2010.03.037.

[256] Chuysinuan P, Pavasant P, Supaphol P. Preparation and Characterization of Caffeic Acid-Grafted Electrospun Poly( <scp>l</scp> -Lactic Acid) Fiber Mats for Biomedical Applications. ACS Appl Mater Interfaces. 2012;4:3031–40. 10.1021/am300404v. 22577837 10.1021/am300404v

[257] Amokrane G, Humblot V, Jubeli E, Yagoubi N, Ramtani S, Migonney V, Falentin-Daudré C. Electrospun poly(ε-caprolactone) fiber scaffolds functionalized by the covalent grafting of a bioactive polymer: Surface characterization and influence on in vitro biological response. ACS Omega. 2019;4:17194–208. 10.1021/acsomega.9b01647. 31656893 10.1021/acsomega.9b01647 PMC6811844

[258] Yuan W, Feng Y, Wang H, Yang D, An B, Zhang W, Khan M, Guo J. Hemocompatible surface of electrospun nanofibrous scaffolds by ATRP modification. Mater Sci Eng C. 2013;33:3644–51. 10.1016/j.msec.2013.04.048. 10.1016/j.msec.2013.04.048 23910260

[259] Mayuri PV, Bhatt A, Joseph R, Ramesh P. Effect of photografting 2-hydroxyethyl acrylate on the hemocompatibility of electrospun poly(ethylene-co-vinyl alcohol) fibroporous mats. Mater Sci Eng C. 2016;60:19–29. 10.1016/j.msec.2015.11.004. 10.1016/j.msec.2015.11.004 26706502

[260] Wang JH. Surface preparation techniques for biomedical applications. In: Coatings for Biomedical Applications, Elsevier. 2012:143–175. 10.1533/9780857093677.1.143.

[261] Vladkova TG. Surface engineered polymeric biomaterials with improved biocontact properties. Int J Polym Sci. 2010;2010:1–22. 10.1155/2010/296094.

[262] Chen F, Lee CN, Teoh SH. Nanofibrous modification on ultra-thin poly(e-caprolactone) membrane via electrospinning. Mater Sci Eng C. 2007;27:325–32. 10.1016/j.msec.2006.05.004.

[263] Borges J, Zeng J, Liu XQ, Chang H, Monge C, Garot C, Ren K, Machillot P, Vrana NE, Lavalle P, Akagi T, Matsusaki M, Ji J, Akashi M, Mano JF, Gribova V, Picart C. Recent developments in layer-by-layer assembly for drug delivery and tissue engineering applications. Adv Healthc Mater. 2024;13:2302713. 10.1002/adhm.202302713. 38116714 10.1002/adhm.202302713 PMC11469081

[264] Gentile P, Carmagnola I, Nardo T, Chiono V. Layer-by-layer assembly for biomedical applications in the last decade. Nanotechnology. 2015;26:422001. 10.1088/0957-4484/26/42/422001. 26421916 10.1088/0957-4484/26/42/422001

[265] Sui C, Wang C, Wang Z, Xu Y, Gong E, Cheng T, Zhou G. Different coating on electrospun nanofiber via layer-by-layer self-assembly for their photocatalytic activities. Colloids Surf A Physicochem Eng Asp. 2017;529:425–33. 10.1016/j.colsurfa.2017.06.030.

[266] Li D, Dai F, Li H, Wang C, Shi X, Cheng Y, Deng H. Chitosan and collagen layer-by-layer assembly modified oriented nanofibers and their biological properties. Carbohydr Polym. 2021;254:117438. 10.1016/j.carbpol.2020.117438. 33357911 10.1016/j.carbpol.2020.117438

[267] Cheng G, Yin C, Tu H, Jiang S, Wang Q, Zhou X, Xing X, Xie C, Shi X, Du Y, Deng H, Li Z. Controlled co-delivery of growth factors through layer-by-layer assembly of core-shell nanofibers for improving bone regeneration. ACS Nano. 2019;13:6372–82. 10.1021/acsnano.8b06032. 31184474 10.1021/acsnano.8b06032

[268] Yuan M, Dai F, Li D, Fan Y, Xiang W, Tao F, Cheng Y, Deng H. Lysozyme/collagen multilayers layer-by-layer deposited nanofibers with enhanced biocompatibility and antibacterial activity. Mater Sci Eng C. 2020;112:110868. 10.1016/j.msec.2020.110868. 10.1016/j.msec.2020.110868 32409037

[269] Müller K, Quinn JF, Johnston APR, Becker M, Greiner A, Caruso F. Polyelectrolyte functionalization of electrospun fibers. Chem Mater. 2006;18:2397–403. 10.1021/cm052760k.

[270] Chunder A, Sarkar S, Yu Y, Zhai L. Fabrication of ultrathin polyelectrolyte fibers and their controlled release properties. Colloids Surf B Biointerfaces. 2007;58:172–9. 10.1016/j.colsurfb.2007.03.004. 17418541 10.1016/j.colsurfb.2007.03.004

[271] Ndunda EN. Molecularly imprinted polymers—A closer look at the control polymer used in determining the imprinting effect: A mini review. J Mol Recognit. 2020;33:e2855. 10.1002/jmr.2855. 32529728 10.1002/jmr.2855

[272] El-Schich Z, Zhang Y, Feith M, Beyer S, Sternbæk L, Ohlsson L, Stollenwerk M, Wingren AG. Molecularly imprinted polymers in biological applications. Biotechniques. 2020;69:407–20. 10.2144/btn-2020-0091. 10.2144/btn-2020-0091 33000637

[273] Keçili R, Ünlüer ÖB, Ersöz A, Say R. Molecularly imprinted polymers (MIPs) for biomedical applications. In: Advances in Biomedical Polymers and Composites, Elsevier. 2023:745–768. 10.1016/B978-0-323-88524-9.00008-5.

[274] Patel KD, Kim H, Knowles JC, Poma A. Molecularly imprinted polymers and electrospinning: manufacturing convergence for next-level applications. Adv Funct Mater. 2020;30:2001955. 10.1002/adfm.202001955.

[275] Kehinde NA, Zenixole T, Nelson T. Fabrication and evaluation of multiple template cross-linked molecularly imprinted electro spun nanofibers for selective extraction of nickel and vanadyl tetraphenylporphyrin from organic media. Afr J Pure Appl Chem. 2015;9:223–39. 10.5897/AJPAC2015.0648.

[276] Zahedi P, Fallah-Darrehchi M, Nadoushan SA, Aeinehvand R, Bagheri L, Najafi M. Morphological, thermal and drug release studies of poly (methacrylic acid)-based molecularly imprinted polymer nanoparticles immobilized in electrospun poly (ε-caprolactone) nanofibers as dexamethasone delivery system. Korean J Chem Eng. 2017;34:2110–8. 10.1007/s11814-017-0078-1.

[277] Gore PM, Khurana L, Siddique S, Panicker A, Kandasubramanian B. Ion-imprinted electrospun nanofibers of chitosan/1-butyl-3-methylimidazolium tetrafluoroborate for the dynamic expulsion of thorium (IV) ions from mimicked effluents. Environ Sci Pollut Res. 2018;25:3320–34. 10.1007/s11356-017-0618-6. 10.1007/s11356-017-0618-6 29150802

[278] Liu Y, Cao B, Jia P, An J, Luo C, Ma L, Chang J, Pan K. Layer-by-Layer Surface Molecular Imprinting on Polyacrylonitrile Nanofiber Mats. J Phys Chem A. 2015;119:6661–7. 10.1021/acs.jpca.5b02325. 26038802 10.1021/acs.jpca.5b02325

[279] Chronakis S, Ioannis, Lei Y. Molecularly Imprinted Nano- and Microstructures by Electrospinning, in: Molecular Imprinting, Jenny Stanford Publishing. 2016:207–229. 10.1201/b15678-9.

[280] Zhai Y, Wang D, Liu H, Zeng Y, Yin Z, Li L. Electrochemical Molecular Imprinted Sensors Based on Electrospun Nanofiber and Determination of Ascorbic Acid. Anal Sci. 2015;31:793–8. 10.2116/analsci.31.793. 26256603 10.2116/analsci.31.793

[281] Urraca JL, Cortés-Llanos B, Aroca C, de la Presa P, Pérez L, Moreno-Bondi MC. Magnetic field-induced polymerization of molecularly imprinted polymers. J Phys Chem C. 2018;122:10189–96. 10.1021/acs.jpcc.7b12804.

[282] Wu K, Yang W, Jiao Y, Zhou C. A surface molecularly imprinted electrospun polyethersulfone (PES) fiber mat for selective removal of bilirubin. J Mater Chem B. 2017;5:5763–73. 10.1039/C7TB00643H. 32264210 10.1039/c7tb00643h

[283] Cowen T, Stefanucci E, Piletska E, Marrazza G, Canfarotta F, Piletsky SA. Synthetic mechanism of molecular imprinting at the solid phase. Macromolecules. 2020;53:1435–42. 10.1021/acs.macromol.9b01913.

[284] Criscenti G, De Maria C, Longoni A, van Blitterswijk CA, Fernandes HAM, Vozzi G, Moroni L. Soft-molecular imprinted electrospun scaffolds to mimic specific biological tissues. Biofabrication. 2018;10:045005. 10.1088/1758-5090/aad48a. 30024388 10.1088/1758-5090/aad48a

[285] Ghorani B, Tucker N, Yoshikawa M. Approaches for the assembly of molecularly imprinted electrospun nanofibre membranes and consequent use in selected target recognition. Food Res Int. 2015;78:448–64. 10.1016/j.foodres.2015.11.014. 28433314 10.1016/j.foodres.2015.11.014

[286] Woertz C, Preis M, Breitkreutz J, Kleinebudde P. Assessment of test methods evaluating mucoadhesive polymers and dosage forms: An overview. Eur J Pharm Biopharm. 2013;85:843–53. 10.1016/j.ejpb.2013.06.023. 23851076 10.1016/j.ejpb.2013.06.023

[287] Mackie AR, Goycoolea FM, Menchicchi B, Caramella CM, Saporito F, Lee S, Stephansen K, Chronakis IS, Hiorth M, Adamczak M, Waldner M, Nielsen HM, Marcelloni L. Innovative methods and applications in mucoadhesion research. Macromol Biosci. 2017;17:1600534. 10.1002/mabi.201600534. 10.1002/mabi.201600534 28378910

[288] Madsen JB, Sotres J, Pakkanen KI, Efler P, Svensson B, Abou Hachem M, Arnebrant T, Lee S. Structural and mechanical properties of thin films of bovine submaxillary mucin versus porcine gastric mucin on a hydrophobic surface in aqueous solutions. Langmuir. 2016;32:9687–96. 10.1021/acs.langmuir.6b02057. 27597630 10.1021/acs.langmuir.6b02057

[289] Schoemig V, Isik E, Martin L, Berensmeier S. Solid liquid liquid extraction of porcine gastric mucins from homogenized animal material. RSC Adv. 2017;7:39708–17. 10.1039/C7RA06594A.

[290] Marczynski M, Rickert CA, Fuhrmann T, Lieleg O. An improved, filtration-based process to purify functional mucins from mucosal tissues with high yields. Sep Purif Technol. 2022;294:121209. 10.1016/j.seppur.2022.121209.

[291] Georgiades P, Pudney PDA, Thornton DJ, Waigh TA. Particle tracking microrheology of purified gastrointestinal mucins. Biopolymers. 2014;101:366–77. 10.1002/bip.22372. 23955640 10.1002/bip.22372

[292] Bansil R, Turner BS. Mucin structure, aggregation, physiological functions and biomedical applications. Curr Opin Colloid Interface Sci. 2006;11:164–70. 10.1016/j.cocis.2005.11.001.

[293] Petrou G, Crouzier T. Mucins as multifunctional building blocks of biomaterials. Biomater Sci. 2018;6:2282–97. 10.1039/C8BM00471D. 30047553 10.1039/c8bm00471d

[294] Liu C, Madl AC, Cirera‐Salinas D, Kress W, Straube F, Myung D, Fuller GG. Mucin‐like glycoproteins modulate interfacial properties of a mimetic ocular epithelial surface. Adv Sci. 2021:8. 10.1002/advs.202100841. 10.1002/advs.202100841 PMC8373091 34184839

[295] Martinez-Carrasco R, Rachagani S, Batra SK, Argüeso P, Fini ME. Roles unveiled for membrane-associated mucins at the ocular surface using a Muc4 knockout mouse model. Sci Rep. 2023;13:13558. 10.1038/s41598-023-40491-0. 37604830 10.1038/s41598-023-40491-0 PMC10442421

[296] Marczynski M, Kimna C, Lieleg O. Purified mucins in drug delivery research. Adv Drug Deliv Rev. 2021;178:113845. 10.1016/j.addr.2021.113845. 34166760 10.1016/j.addr.2021.113845

[297] Schömig VJ, Käsdorf BT, Scholz C, Bidmon K, Lieleg O, Berensmeier S. An optimized purification process for porcine gastric mucin with preservation of its native functional properties. RSC Adv. 2016;6:44932–43. 10.1039/C6RA07424C.

[298] Rossi S, Vigani B, Bonferoni MC, Sandri G, Caramella C, Ferrari F. Rheological analysis and mucoadhesion: A 30 year-old and still active combination. J Pharm Biomed Anal. 2018;156:232–8. 10.1016/j.jpba.2018.04.041. 29729636 10.1016/j.jpba.2018.04.041

[299] Melvin M, Fancy N, Kniffen D, Bergstrom K. Extraction and verification of mouse and human mucins from tissue and fecal material. In: Carbohydrate-Protein Interactions 2nd ed. 2023:197–205. 10.1007/978-1-0716-3151-5_14. 10.1007/978-1-0716-3151-5_14 37149532

[300] Wagner CE, Krupkin M, Smith-Dupont KB, Wu CM, Bustos NA, Witten J, Ribbeck K. Comparison of physicochemical properties of native mucus and reconstituted mucin gels. Biomacromol. 2023;24:628–39. 10.1021/acs.biomac.2c01016. 10.1021/acs.biomac.2c01016 36727870

[301] Franconi F, Lemaire L, Gimel J-C, Bonnet S, Saulnier P. NMR diffusometry: A new perspective for nanomedicine exploration. J Control Release. 2021;337:155–67. 10.1016/j.jconrel.2021.07.025. 34280413 10.1016/j.jconrel.2021.07.025

[302] Cesari A, Fabiano A, Piras AM, Zambito Y, Uccello-Barretta G, Balzano F. Binding and mucoadhesion of sulfurated derivatives of quaternary ammonium-chitosans and their nanoaggregates: An NMR investigation. J Pharm Biomed Anal. 2020;177:112852. 10.1016/j.jpba.2019.112852. 31499432 10.1016/j.jpba.2019.112852

[303] Mukherjee S, Martinez-Gonzalez JA, Gowen AA. Feasibility of attenuated total reflection-fourier transform infrared (ATR-FTIR) chemical imaging and partial least squares regression (PLSR) to predict protein adhesion on polymeric surfaces. Analyst. 2019;144:1535–45. 10.1039/C8AN01768A. 30542682 10.1039/c8an01768a

[304] Uthaiwat P, Priprem A, Puthongking P, Daduang J, Nukulkit C, Chio-Srichan S, Boonsiri P, Thapphasaraphong S. Characteristic evaluation of gel formulation containing niosomes of melatonin or its derivative and mucoadhesive properties using ATR-FTIR spectroscopy. Polymers (Basel). 2021;13:1142. 10.3390/polym13071142. 33918458 10.3390/polym13071142 PMC8038236

[305] Chen D, Xia D, Li X, Zhu Q, Yu H, Zhu C, Gan Y. Comparative study of Pluronic® F127-modified liposomes and chitosan-modified liposomes for mucus penetration and oral absorption of cyclosporine A in rats. Int J Pharm. 2013;449:1–9. 10.1016/j.ijpharm.2013.04.002. 23583840 10.1016/j.ijpharm.2013.04.002

[306] Pack CG. Confocal Laser Scanning Microscopy and Fluorescence Correlation Methods for the Evaluation of Molecular Interactions. In: Advanced Imaging and Bio Techniques for Convergence Science, 1st ed., Springer. 2021:1–30. 10.1007/978-981-33-6064-8_1. 10.1007/978-981-33-6064-8_1 33834430

[307] Li D, Yamamoto H, Takeuchi H, Kawashima Y. A novel method for modifying AFM probe to investigate the interaction between biomaterial polymers (Chitosan-coated PLGA) and mucin film. Eur J Pharm Biopharm. 2010;75:277–83. 10.1016/j.ejpb.2010.02.013. 20188826 10.1016/j.ejpb.2010.02.013

[308] Dunker K, de la Torre Canny SG, Nordgård CT, Dague E, Formosa-Dague C, Bakke I, Sletmoen M. Elucidating bacterial adhesion to mucosal surface by an original AFM approach. BMC Microbiol. 2021;21:244. 10.1186/s12866-021-02303-1. 34488629 10.1186/s12866-021-02303-1 PMC8422614

[309] da Silva Bassi J, de Ferreira SBS, de Freitas O, Bruschi ML. A critical review about methodologies for the analysis of mucoadhesive properties of drug delivery systems. Drug Dev Ind Pharm. 2017;43:1053–70. 10.1080/03639045.2017.1294600. 28276785 10.1080/03639045.2017.1294600

[310] Surendranath M, Ramesan RM, Nair P, Parameswaran R. Mucin incorporated electrospun fibrous matrix of zein and PVP: towards transmucosal propranolol hydrochloride delivery. J Drug Deliv Sci Technol. 2024;100:106016. 10.1016/j.jddst.2024.106016.

[311] Mirzaeei S, Taghe S, Asare-Addo K, Nokhodchi A. Polyvinyl alcohol/chitosan single-layered and polyvinyl alcohol/chitosan/eudragit RL100 Multi-layered electrospun nanofibers as an ocular matrix for the controlled release of ofloxacin: An in vitro and in vivo evaluation. AAPS PharmSciTech. 2021;22:170. 10.1208/s12249-021-02051-5. 34085150 10.1208/s12249-021-02051-5 PMC8175245

[312] Salama AH, AbouSamra MM, Awad GEA, Mansy SS. Promising bioadhesive ofloxacin-loaded polymeric nanoparticles for the treatment of ocular inflammation: formulation and in vivo evaluation. Drug Deliv Transl Res. 2021;11:1943–57. 10.1007/s13346-020-00856-8. 33006742 10.1007/s13346-020-00856-8

[313] Dey S, Ghosh B, Mukherjee K, Giri TK. Development and evaluation of locust bean gum based in situ gel for ocular delivery of ofloxacin for treatment of bacterial keratitis. Int J Biol Macromol. 2024;281:136374. 10.1016/j.ijbiomac.2024.136374. 39383900 10.1016/j.ijbiomac.2024.136374

[314] Çağlar EŞ, Yoltaş A, Özhan Y, Sipahi H, Aydın A, Üstündağ Okur N, Siafaka P. Mucoadhesive electrospun nanofibrous poly(ε-caprolactone)/poly(lactic acid) matrices for the ocular delivery of moxifloxacin: a novel application of hyaluronic acid and xanthan gum blend as mucoadhesive coating agent. Int J Polym Mater Polym Biomater. 2024;74(5):361–76. 10.1080/00914037.2024.2335180.

[315] Youssef AAA, Thakkar R, Senapati S, Joshi PH, Dudhipala N, Majumdar S. Design of topical moxifloxacin mucoadhesive nanoemulsion for the management of ocular bacterial infections. Pharmaceutics. 2022;14:1246. 10.3390/pharmaceutics14061246. 35745818 10.3390/pharmaceutics14061246 PMC9228176

[316] Gade SK, Nirmal J, Garg P, Venuganti VVK. Corneal delivery of moxifloxacin and dexamethasone combination using drug-eluting mucoadhesive contact lens to treat ocular infections. Int J Pharm. 2020;591:120023. 10.1016/j.ijpharm.2020.120023. 33127488 10.1016/j.ijpharm.2020.120023

[317] Mehrandish S, Mohammadi G, Mirzaeei S. Preparation and functional evaluation of electrospun polymeric nanofibers as a new system for sustained topical ocular delivery of itraconazole. Pharm Dev Technol. 2022;27:25–39. 10.1080/10837450.2021.2018609. 34895024 10.1080/10837450.2021.2018609

[318] Badran MM, Alsubaie A, Bekhit MMS, Alomrani AH, Almomen A. Layer-by-layer biopolymer-coated deformable liposomes–in situ gel: A hybrid strategy for enhanced ocular delivery of itraconazole in vitro and in vivo appraisal. Gels. 2024;11:19. 10.3390/gels11010019. 39851990 10.3390/gels11010019 PMC11765087

[319] Permana AD, Utami RN, Layadi P, Himawan A, Juniarti N, Anjani QK, Utomo E, Mardikasari SA, Arjuna A, Donnelly RF. Thermosensitive and mucoadhesive in situ ocular gel for effective local delivery and antifungal activity of itraconazole nanocrystal in the treatment of fungal keratitis. Int J Pharm. 2021;602:120623. 10.1016/j.ijpharm.2021.120623. 33892058 10.1016/j.ijpharm.2021.120623

[320] Cegielska O, Sierakowski M, Sajkiewicz P, Lorenz K, Kogermann K. Mucoadhesive brinzolamide-loaded nanofibers for alternative glaucoma treatment. Eur J Pharm Biopharm. 2022;180:48–62. 10.1016/j.ejpb.2022.09.008. 36167272 10.1016/j.ejpb.2022.09.008

[321] Huang D, Norat P, Qi L, Chernatynskaya A, Cole JD, Mani VJ, Xu L, Liu X, Yang H. Consistent Intraocular Pressure reduction by solid drug nanoparticles in fixed combinations for glaucoma therapy. Adv Sci. 2024;11:2401648. 10.1002/advs.202401648. 10.1002/advs.202401648 PMC11336906 38874068

[322] Lin J, Xue J, Xu Q, Liu Z, Zhao C, Tang J, Han J, Wang SAW, Zhuo Y, Li Y. In situ -crosslinked hydrogel-induced experimental glaucoma model with persistent ocular hypertension and neurodegeneration. Biomater Sci. 2022;10:5006–17. 10.1039/D2BM00552B. 35815806 10.1039/d2bm00552b

[323] Voronova A, Prieto C, Pardo-Figuerez M, Lagaron JM, Sanyal A, Demir B, Hubert T, Plaisance V, Pawlowski V, Vignoud-Despond S, Barras A, Abderrahmani A, Boukherroub R, Szunerits S. Photothermal activatable mucoadhesive fiber mats for on-demand delivery of insulin via buccal and corneal mucosa. ACS Appl Bio Mater. 2022;5:771–8. 10.1021/acsabm.1c01161. 35026943 10.1021/acsabm.1c01161

[324] Chen S, Li Y, Song W, Cheng Y, Gao Y, Xie L, Huang M, Yan X. Insulin eye drops improve corneal wound healing in STZ-induced diabetic mice by regulating corneal inflammation and neuropeptide release. BMC Ophthalmol. 2024;24:155. 10.1186/s12886-024-03436-3. 38594682 10.1186/s12886-024-03436-3 PMC11003036

[325] Cruz-Cazarim ELC, Cazarim MS, Ogunjimi AT, Petrilli R, Rocha EM, Lopez RFV. Prospective insulin-based ophthalmic delivery systems for the treatment of dry eye syndrome and corneal injuries. Eur J Pharm Biopharm. 2019;140:1–10. 10.1016/j.ejpb.2019.04.014. 31015020 10.1016/j.ejpb.2019.04.014

[326] Zhang S, Li X, Yang N, Ling L, Zhang M, Ye Q, Wang Y. Electrospun collagen nanofibers reduce inflammation inhibit fibrosis, and promote wound healing on the ocular surface. ACS Appl Nano Mater. 2024;7:20267–78. 10.1021/acsanm.4c03180.

[327] Li L, Lu C, Wang L, Chen M, White J, Hao X, McLean KM, Chen H, Hughes TC. Gelatin-Based Photocurable Hydrogels for Corneal Wound Repair. ACS Appl Mater Interfaces. 2018;10:13283–92. 10.1021/acsami.7b17054. 29620862 10.1021/acsami.7b17054

[328] Shirzaei Sani E, Kheirkhah A, Rana D, Sun Z, Foulsham W, Sheikhi A, Khademhosseini A, Dana R, Annabi N. Sutureless repair of corneal injuries using naturally derived bioadhesive hydrogels. Sci Adv. 2019;5:eaav1281. 10.1126/sciadv.aav1281. 30906864 10.1126/sciadv.aav1281 PMC6426459

[329] Jawadi Z, Yang C, Haidar ZS, Santa Maria PL, Massa S. Bio-inspired muco-adhesive polymers for drug delivery applications. Polymers (Basel). 2022;14:5459. 10.3390/polym14245459. 36559825 10.3390/polym14245459 PMC9785024

[330] Benítez-Martínez JA, Garnica-Palafox IM, Rodríguez-Hernández A, Pérez-Calixto D, Vázquez-Victorio G, Hernádez-Gordillo A, Sánchez-Arévalo FM. Physicochemical characterization and biological response of PDMS/CS/PVA/GEN semi-interpenetrating networks as a function of CS/PVA/GEN ratio for tissue engineering. Appl Phys A. 2023;129:558. 10.1007/s00339-023-06821-9.

[331] Joyce K, Fabra GT, Bozkurt Y, Pandit A. Bioactive potential of natural biomaterials: identification, retention and assessment of biological properties. Signal Transduct Target Ther. 2021;6:122. 10.1038/s41392-021-00512-8. 33737507 10.1038/s41392-021-00512-8 PMC7973744

[332] Satchanska G, Davidova S, Petrov PD. Natural and synthetic polymers for biomedical and environmental applications. Polymers (Basel). 2024;16:1159. 10.3390/polym16081159. 38675078 10.3390/polym16081159 PMC11055061

[333] Liu S, Yu J-M, Gan Y-C, Qiu X-Z, Gao Z-C, Wang H, Chen S-X, Xiong Y, Liu G-H, Lin S-E, McCarthy A, John JV, Wei D-X, Hou H-H. Biomimetic natural biomaterials for tissue engineering and regenerative medicine: new biosynthesis methods, recent advances, and emerging applications. Mil Med Res. 2023;10:16. 10.1186/s40779-023-00448-w. 36978167 10.1186/s40779-023-00448-w PMC10047482

[334] Ho TT, Tran HA, Doan VK, Maitz J, Li Z, Wise SG, Lim KS, Rnjak-Kovacina J. Natural polymer-based materials for wound healing applications. Adv Nanobiomed Res. 2024;4:2300131. 10.1002/anbr.202300131.

[335] Rahmati M, Silva EA, Reseland JE, Heyward CA, Haugen HJ. Biological responses to physicochemical properties of biomaterial surface. Chem Soc Rev. 2020;49:5178–224. 10.1039/D0CS00103A. 32642749 10.1039/d0cs00103a

[336] Li C, Guo C, Fitzpatrick V, Ibrahim A, Zwierstra MJ, Hanna P, Lechtig A, Nazarian A, Lin SJ, Kaplan DL. Design of biodegradable, implantable devices towards clinical translation. Nat Rev Mater. 2019;5:61–81. 10.1038/s41578-019-0150-z.

[337] Vigani B, Rossi S, Sandri G, Bonferoni MC, Caramella CM. Mucoadhesive polymers in substance-based medical devices: functional ingredients or what else? Front Drug Saf Regul. 2023;3:1227763. 10.3389/fdsfr.2023.1227763. 40980116 10.3389/fdsfr.2023.1227763 PMC12443092

[338] Hutcheon AEK, Zieske JD, Guo X. 3D in vitro model for human corneal endothelial cell maturation. Exp Eye Res. 2019;184:183–91. 10.1016/j.exer.2019.04.003. 30980816 10.1016/j.exer.2019.04.003 PMC6684281

[339] Shiju TM, Carlos de Oliveira R, Wilson SE. 3D in vitro corneal models: A review of current technologies. Exp Eye Res. 2020;200:108213. 10.1016/j.exer.2020.108213. 32890484 10.1016/j.exer.2020.108213 PMC7655665

[340] Peng Z, Zhou L, Wong JKW, Chan YK. Eye-on-a-chip (EOC) models and their role in the future of ophthalmic drug discovery. Expert Rev Ophthalmol. 2020;15:259–61. 10.1080/17469899.2020.1788388.

[341] Madhurima P, Tripathi S, Mishra P, Choudhury K, Kumar P, Kumar S, Banoth E. Advances in nondestructive optical characterization techniques for engineered eye-on-a-chip devices: A comprehensive review. Opt Laser Technol. 2024;175:110750. 10.1016/j.optlastec.2024.110750.

[342] Kim M, Choi K, Lin A, Kim J. Current and future cornea chip models for advancing ophthalmic research and therapeutics. Adv Biol. 2025. 10.1002/adbi.202400571. 10.1002/adbi.202400571 PMC12264468 39962012

[343] Gai X, Liu C, Wang G, Qin Y, Fan C, Liu J, Shi Y. A novel method for evaluating the dynamic biocompatibility of degradable biomaterials based on real-time cell analysis. Regen Biomater. 2020;7:321–9. 10.1093/rb/rbaa017. 32523733 10.1093/rb/rbaa017 PMC7266667

[344] Karakullukcu AB, Taban E, Ojo OO. Biocompatibility of biomaterials and test methods: a review. Mater Test. 2023;65:545–59. 10.1515/mt-2022-0195.

[345] Zamboni WC, Szebeni J, Kozlov SV, Lucas AT, Piscitelli JA, Dobrovolskaia MA. Animal models for analysis of immunological responses to nanomaterials: Challenges and considerations. Adv Drug Deliv Rev. 2018;136–137:82–96. 10.1016/j.addr.2018.09.012. 30273617 10.1016/j.addr.2018.09.012

[346] De Jong WH, Carraway JW, Geertsma RE. In vivo and in vitro testing for the biological safety evaluation of biomaterials and medical devices. In: Biocompatibility and Performance of Medical Devices, Elsevier. 2020:123–166. 10.1016/B978-0-08-102643-4.00007-0.

[347] Xing J, Zhang M, Liu X, Wang C, Xu N, Xing D. Multi-material electrospinning: from methods to biomedical applications. Mater Today Bio. 2023;21:100710. 10.1016/j.mtbio.2023.100710. 37545561 10.1016/j.mtbio.2023.100710 PMC10401296

[348] Al-Abduljabbar A, Farooq I. Electrospun polymer nanofibers: processing, properties, and applications. Polymers (Basel). 2022;15:65. 10.3390/polym15010065. 36616414 10.3390/polym15010065 PMC9823865

[349] Kraisit P, Limmatvapirat S, Nunthanid J, Sriamornsak P, Luangtana-Anan M. Preparation and characterization of hydroxypropyl methylcellulose/polycarbophil mucoadhesive blend films using a mixture design approach. Chem Pharm Bull (Tokyo). 2017;65:284–94. 10.1248/cpb.c16-00849. 27980251 10.1248/cpb.c16-00849

[350] Bakhrushina E, Anurova M, Demina N, Kashperko A, Rastopchina O, Bardakov A, Krasnyuk I. Comparative study of the mucoadhesive properties of polymers for pharmaceutical use. Open Access Maced J Med Sci. 2020;8:639–45. 10.3889/oamjms.2020.4930.

[351] Borrotti M, Lanzarone E, Manganini F, Ortelli S, Pievatolo A, Tonetti C. Defect minimization and feature control in electrospinning through design of experiments. J Appl Polym Sci. 2017:134. 10.1002/app.44740.

[352] Zhang H-T, Wang Q, Chen Z, Wei F-L. Dynamics and Feedback Control of Electrospinning Processes. IEEE Trans Control Syst Technol. 2017;25:611–8. 10.1109/TCST.2016.2557224.

[353] Foulkes R, Man E, Thind J, Yeung S, Joy A, Hoskins C. The regulation of nanomaterials and nanomedicines for clinical application: current and future perspectives. Biomater Sci. 2020;8:4653–64. 10.1039/D0BM00558D. 32672255 10.1039/d0bm00558d

[354] Desai N, Pande S, Gholap AD, Rana D, Salave S, Vora LK. Regulatory processes involved in clinical trials and intellectual property rights around vaccine development. In: Advanced Vaccination Technologies for Infectious and Chronic Diseases, Elsevier. 2024:279–309. 10.1016/B978-0-443-18564-9.00008-4.

[355] Cuellar-Gaona CG, Ibarra-Alonso MC, Reyna-Martínez R, Narro-Céspedes RI, Martínez-Luévanos A, Dávila-Medina MD, Castañeda-Facio AO, Reyes-Acosta YK, Ávalos-Belmontes F, Saucedo-Salazar EM. Review and Analysis of biological tests on nanomaterials to be applied in biological areas. In: Green-Based Nanocomposite Materials and Applications, 1st ed., Springer Cham. 2023:339–363. 10.1007/978-3-031-18428-4_17.

[356] Rajani C, Borisa P, Bagul S, Shukla K, Tambe V, Desai N, Tekade RK. Developmental toxicity of nanomaterials used in drug delivery: understanding molecular biomechanics and potential remedial measures. In: Pharmacokinetics and Toxicokinetic Considerations, Elsevier. 2022:685–725. 10.1016/B978-0-323-98367-9.00017-2.

[357] Pognan F, Beilmann M, Boonen HCM, Czich A, Dear G, Hewitt P, Mow T, Oinonen T, Roth A, Steger-Hartmann T, Valentin J-P, Van Goethem F, Weaver RJ, Newham P. The evolving role of investigative toxicology in the pharmaceutical industry. Nat Rev Drug Discov. 2023;22:317–35. 10.1038/s41573-022-00633-x. 36781957 10.1038/s41573-022-00633-x PMC9924869

[358] Kuperkar K, Atanase L, Bahadur A, Crivei I, Bahadur P. Degradable polymeric Bio(nano)materials and their biomedical applications: A comprehensive overview and recent updates. Polymers (Basel). 2024;16:206. 10.3390/polym16020206. 38257005 10.3390/polym16020206 PMC10818796

[359] Csóka I, Ismail R, Jójárt-Laczkovich O, Pallagi E. Regulatory considerations, challenges and risk-based approach in nanomedicine development. Curr Med Chem. 2021;28:7461–76. 10.2174/0929867328666210406115529. 33823761 10.2174/0929867328666210406115529

[360] Halamoda-Kenzaoui B, Holzwarth U, Roebben G, Bogni A, Bremer-Hoffmann S. Mapping of the available standards against the regulatory needs for nanomedicines. WIREs Nanomed and Nanobiotechnol. 2019;11:e1531. 10.1002/wnan.1531. 10.1002/wnan.1531 PMC6585614 29923692

[361] Sainz V, Conniot J, Matos AI, Peres C, Zupanǒiǒ E, Moura L, Silva LC, Florindo HF, Gaspar RS. Regulatory aspects on nanomedicines. Biochem Biophys Res Commun. 2015;468:504–10. 10.1016/j.bbrc.2015.08.023. 26260323 10.1016/j.bbrc.2015.08.023

[362] Younis MA, Tawfeek HM, Abdellatif AAH, Abdel-Aleem JA, Harashima H. Clinical translation of nanomedicines: Challenges, opportunities, and keys. Adv Drug Deliv Rev. 2022;181:114083. 10.1016/j.addr.2021.114083. 34929251 10.1016/j.addr.2021.114083

